# Supplementary material for: Anti-commensal IgG Drives Intestinal Inflammation and Type 17 Immunity in Ulcerative Colitis
Source: Immunity. 2019 Apr 16;50(4):1099–1114.e10. doi: 10.1016/j.immuni.2019.02.006 (PMC6477154; doi:10.1016/j.immuni.2019.02.006)
Supplement: Document S1. Figures S1–S6 and Tables S1 and S2 [file mmc1.pdf]

Immunity, Volume *50*

## Supplemental Information

# Anti-commensal IgG Drives Intestinal Inflammation and Type 17 Immunity in Ulcerative Colitis

**Tomas Castro-Dopico, Thomas W. Dennison, John R. Ferdinand, Rebeccah J. Mathews, Aaron Fleming, Dean Clift, Benjamin J. Stewart, Chenzhi Jing, Konstantina Strongili, Larisa I. Labzin, Edward J.M. Monk, Kourosh Saeb-Parsy, Clare E. Bryant, Simon Clare, Miles Parkes, and Menna R. Clatworthy**

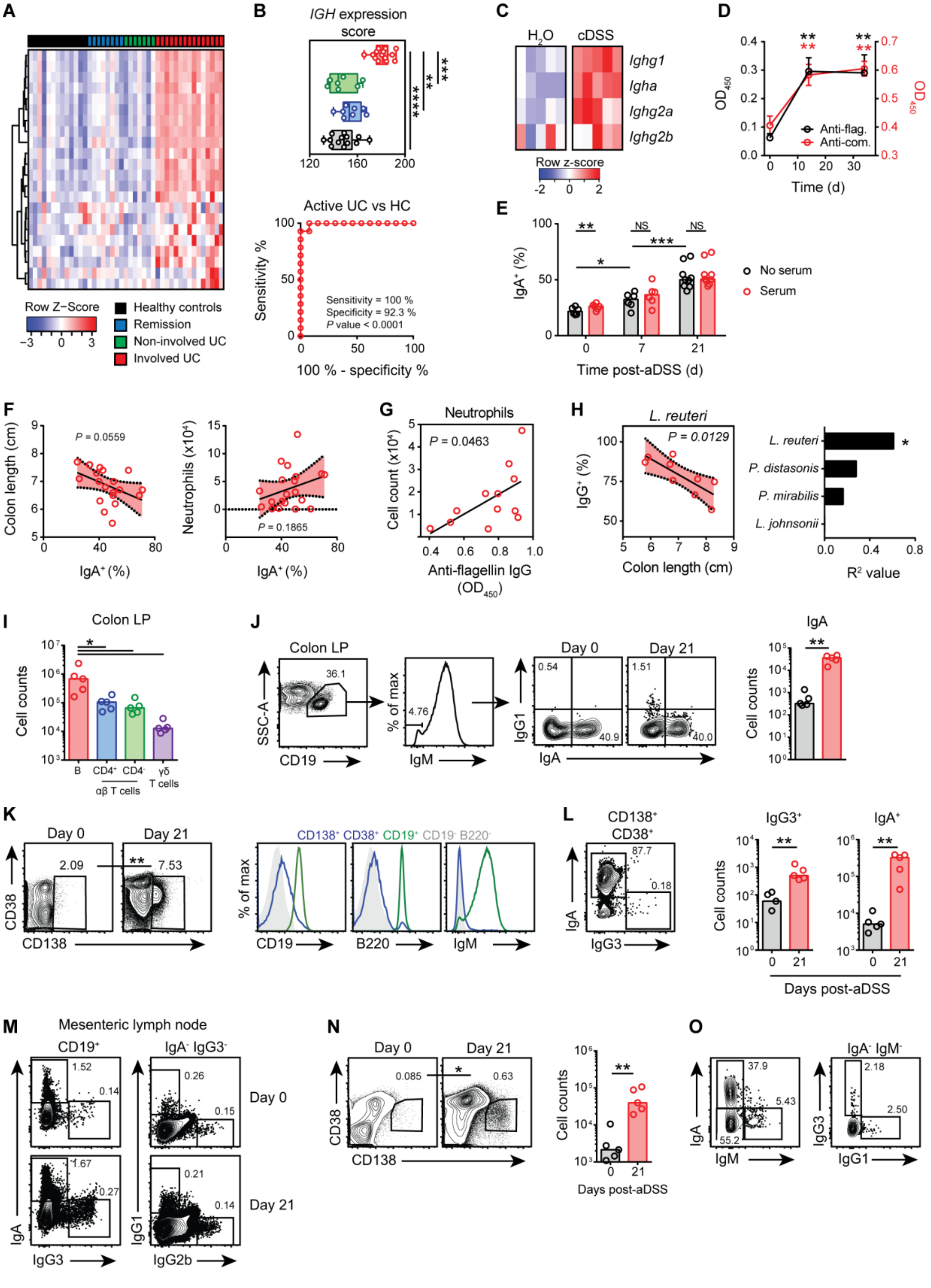
A
Row Z-Score
−3
0
3
Healthy controls
Remission
Non-involved UC
Involved UC
B
IGH expression score
120
160
200
****
**
***
Active UC vs HC
Sensitivity %
100 % - specificity %
Sensitivity = 100 %
Specificity = 92.3 %
P value < 0.0001
C
H2O
cDSS
Ighg1
Igha
Ighg2a
Ighg2b
Row z-score
-2
0
2
D
OD450
Anti-flag.
Anti-com.
Time (d)
E
IgA+ (%)
No serum
Serum
NS
Time post-aDSS (d)
F
Colon length (cm)
P = 0.0559
Neutrophils (x10^4)
P = 0.1865
IgA+ (%)
G
Neutrophils
P = 0.0463
Cell count (x10^4)
Anti-flagellin IgG (OD450)
H
L. reuteri
P = 0.0129
IgG+ (%)
Colon length (cm)
L. reuteri
P. distasonis
P. mirabilis
L. johnsonii
R2 value
I
Colon LP
Cell counts
B
CD4+
CD4-
αβ T cells
γδ T cells
J
Colon LP
SSC-A
CD19
36.1
% of max
IgM
4.76
Day 0
Day 21
IgG1
IgA
0.54
40.9
1.51
40.0
IgA
Cell counts
K
Day 0
Day 21
CD38
CD138
2.09
7.53
CD138+ CD38+
CD19+
CD19- B220-
% of max
CD19
B220
IgM
L
CD138+ CD38+
87.7
0.18
IgA
IgG3
IgG3+
IgA+
Cell counts
Days post-aDSS
M
Mesenteric lymph node
CD19+
IgA- IgG3-
1.52
0.14
0.26
0.15
Day 0
1.67
0.27
0.21
0.14
Day 21
IgA
IgG3
IgG1
IgG2b
N
Day 0
Day 21
0.085
0.63
CD38
CD138
Cell counts
Days post-aDSS
O
IgA- IgM-
37.9
5.43
55.2
2.18
2.50
IgA
IgM
IgG3
IgG1

**Figure S1. Anti-commensal Ig and B cell infiltration in intestinal inflammation, related to Figure 1.** (A) Heatmap of *IGH* gene probes from transcriptomic analysis of inflamed active UC mucosal biopsies ($n$ = 15) compared to mucosal biopsies from healthy controls ($n$ = 13), remission ($n$ = 8), and non-inflamed biopsies ($n$ = 7). Data were derived from the GEO dataset GSE38713. (B) Cumulative expression score of *IGH* genes shown in A (top) and AUROC analysis of computed scores between inflamed UC biopsies and healthy control biopsies (bottom). Min. to max. box-and-whisker plots are shown for patient scoring. (C) Heatmap of *Igh* genes from transcriptomic analysis of a previously published dataset of murine DSS-inflamed colons versus healthy controls ($n$ = 5 per group). Data were derived from the GEO dataset GSE42768. (D) Anti-mouse IgG ELISA showing *de novo* anti-commensal (red) and anti-flagellin (black) IgG generation post-aDSS ($n$ = 5 per group). Means ± s.e.m. are indicated. Data are representative of three independent experiments. (E) Microbial flow cytometric quantification of IgA-bound SYBR green$^{hi}$ luminal bacteria post-aDSS or in healthy controls, with (red) and without (black) paired serum pre-incubation ($n$ = 6-9 per group). Medians are indicated. Data are pooled from two independent experiments. (F) Correlation analysis of luminal IgA-opsonized commensal microbes from the day 21 timepoint (no serum) with large intestine length (left) and neutrophil infiltration (right) ($n$ = 20). Data are pooled from two independent experiments. (G) Correlation analysis of colonic neutrophil infiltration with systemic serum anti-flagellin IgG titers at day 28 post-aDSS ($n$ = 12). Data are representative of two independent experiments. (H) Correlation of commensal-serum IgG opsonization levels with colon length, pooled from healthy controls and colitic mice at day 21 post-aDSS ($n$ = 9). (I, J) Flow cytometric quantification of absolute counts of colonic lymphocyte subsets (I) and colonic IgM$^-$ class-switched CD19$^+$ B cell subsets (J) at day 21 post-aDSS administration or in healthy controls ($n$ = 5 per group). Medians are indicated. Data are representative of two or three independent experiments. (K, L) Identification (K) and class-switch analysis (L) of colonic lamina propria CD138$^+$ CD38$^+$ plasma cell subsets in colitic mice at day 21 post-aDSS or healthy control mice (day 0) ($n$ = 5 per group). Medians are indicated for the absolute counts of IgG3$^+$ and IgA$^+$ plasma cells in L. Data are representative of three independent experiments. (M) Profiling of MLN IgM$^-$ class-switched CD19$^+$ B cell subsets, as in J. (N, O) Identification (N) and class-switch analysis (O) of MLN CD138$^+$ CD38$^+$ plasma cells, as in K and L. Medians are indicated for absolute counts of MLN plasma cells in N. Data are representative of two or three independent experiments. $P$ values were calculated using a Kruskal-Wallis test with Dunn's multiple comparisons test (B, top), AUROC analysis (B bottom), the nonparametric Mann-Whitney $U$ test (D, E, J-O), linear regression analysis (F-H), or a one-way ANOVA with Tukey's multiple comparisons test (I). * $P < 0.05$; ** $P < 0.01$; *** $P < 0.001$; **** $P < 0.0001$.

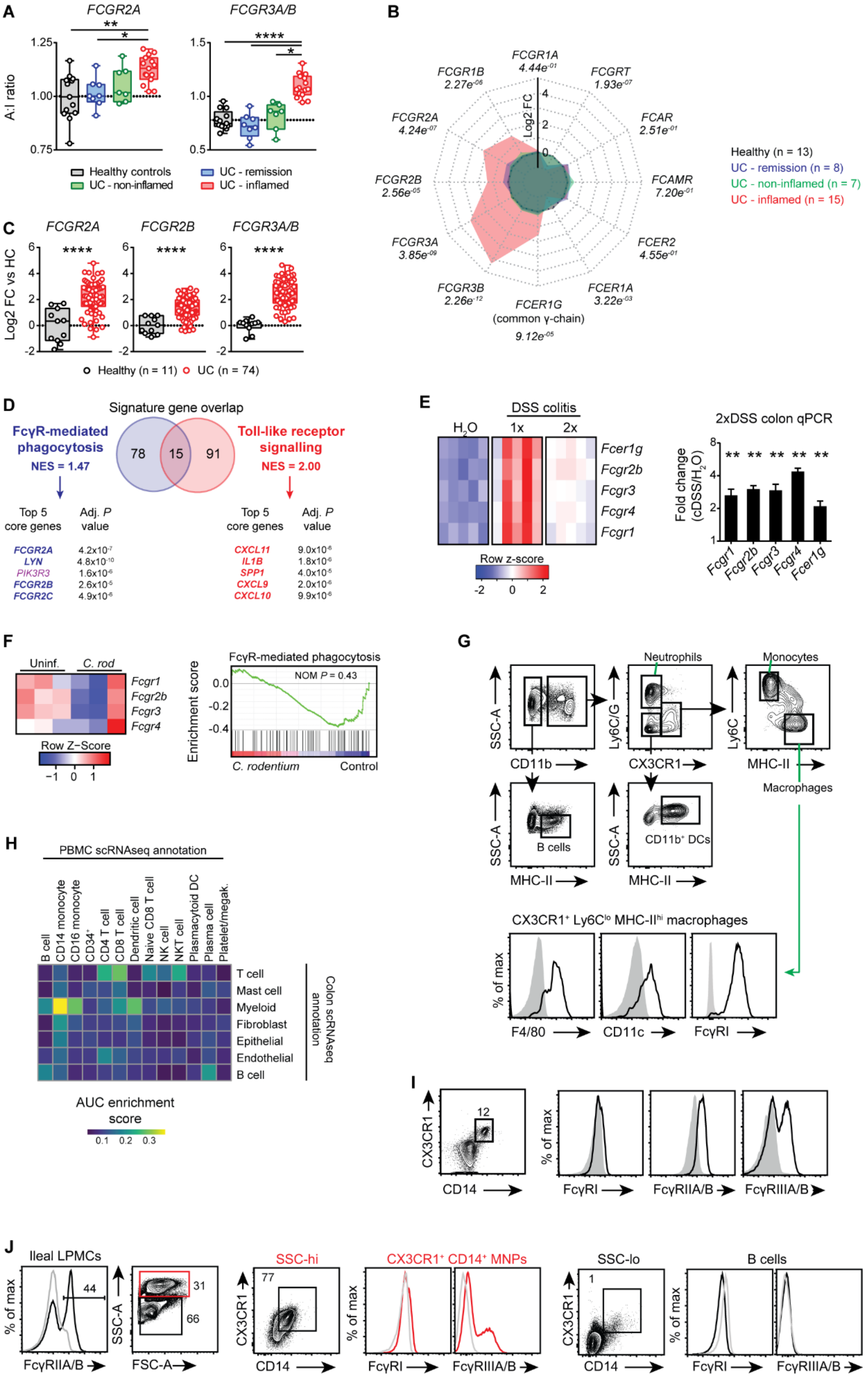
A
FCGR2A
FCGR3A/B
A:I ratio
Healthy controls
UC - remission
UC - non-inflamed
UC - inflamed
B
FCGR1A 4.44e-01
FCGRT 1.93e-07
FCAR 2.51e-01
FCAMR 7.20e-01
FCER2 4.55e-01
FCER1A 3.22e-03
FCER1G (common γ-chain) 9.12e-05
FCGR3B 2.26e-12
FCGR3A 3.85e-09
FCGR2B 2.56e-05
FCGR2A 4.24e-07
FCGR1B 2.27e-06
Log2 FC
Healthy (n = 13)
UC - remission (n = 8)
UC - non-inflamed (n = 7)
UC - inflamed (n = 15)
C
FCGR2A
FCGR2B
FCGR3A/B
Log2 FC vs HC
Healthy (n = 11)
UC (n = 74)
D
Signature gene overlap
FcγR-mediated phagocytosis
NES = 1.47
Toll-like receptor signalling
NES = 2.00
78
15
91
Top 5 core genes
Adj. P value
FCGR2A 4.2x10-7
LYN 4.8x10-10
PIK3R3 1.6x10-6
FCGR2B 2.6x10-5
FCGR2C 4.9x10-5
CXCL11 9.0x10-6
IL1B 1.8x10-6
SPP1 4.0x10-5
CXCL9 2.0x10-6
CXCL10 9.9x10-6
E
DSS colitis
H2O
1x
2x
Fcer1g
Fcgr2b
Fcgr3
Fcgr4
Fcgr1
Row z-score
2xDSS colon qPCR
Fold change (cDSS/H2O)
Fcgr1
Fcgr2b
Fcgr3
Fcgr4
Fcer1g
F
Uninf.
C. rod
Fcgr1
Fcgr2b
Fcgr3
Fcgr4
Row Z-Score
FcγR-mediated phagocytosis
NOM P = 0.43
Enrichment score
C. rodentium
Control
G
Neutrophils
Monocytes
SSC-A
CD11b
Ly6C/G
CX3CR1
Ly6C
MHC-II
Macrophages
B cells
CD11b+ DCs
CX3CR1+ Ly6Clo MHC-IIhi macrophages
% of max
F4/80
CD11c
FcγRI
H
PBMC scRNAseq annotation
B cell
CD14 monocyte
CD16 monocyte
CD34+
CD4 T cell
CD8 T cell
Dendritic cell
Naive CD8 T cell
NK cell
NKT cell
Plasmacytoid DC
Plasma cell
Platelet/megak.
T cell
Mast cell
Myeloid
Fibroblast
Epithelial
Endothelial
B cell
Colon scRNAseq annotation
AUC enrichment score
I
12
CX3CR1
CD14
% of max
FcγRI
FcγRIIA/B
FcγRIIIA/B
J
Ileal LPMCs
44
31
66
% of max
FcγRIIA/B
SSC-A
FSC-A
SSC-hi
77
CX3CR1
CD14
CX3CR1+ CD14+ MNPs
FcγRI
FcγRIIIA/B
SSC-lo
1
B cells

**Figure S2. FcγR profiling in intestinal inflammation, related to Figure 2.** (A) A:I ratio of *FCGR* genes in transcriptomics analysis of mucosal biopsies from inflamed active UC mucosal biopsies ($n$ = 15) compared to mucosal biopsies from healthy controls ($n$ = 13), remission ($n$ = 8), and non-inflamed biopsies ($n$ = 7). Data were derived from the GEO dataset GSE38713. Min. to max. box-and-whisker plots are shown. (B) Comparison of Fc receptor gene enrichment in IBD biopsies from dataset in A. $P$ values listed below gene names. (C) Transcriptomics analysis of *FCGR* gene transcripts in intestinal mucosal biopsies from an independent previously published UC cohort. Data were derived from GEO dataset GSE59071 (UC = 74; HC = 11). Max. to min. boxplots are shown. (D) Overlap of KEGG *FcγR-mediated phagocytosis* and *Toll-like receptor signaling* pathway genes, with the top 5 core enrichment genes within inflamed mucosal biopsies from dataset in A. (E) Heatmap showing transcriptomic analysis of murine *Fcgr* genes in uninflamed ($H_2O$), aDSS, and cDSS colon tissue (left, $n$ = 5 per group) and qPCR of *Fcgr* expression in the inflamed colons of mice exposed to cDSS over healthy colons by qPCR (right, $n$ = 5 per group). Transcriptomics data was generated from the GEO dataset GSE42768. Mean ± s.e.m. are indicated. Data are representative of two independent experiments. (F) FcγR gene enrichment and GSEA of KEGG *FcγR-mediated phagocytosis* in murine colonic tissue following *C. rodentium* infection ($n$ = 3 per group). Data was derived from the GEO dataset GSE49109. (G) Flow cytometric gating strategy for FcγR-expressing cells within the inflamed murine colonic lamina propria. Grey = isotype control. (H) Immune cell subset identification in colonic single cell RNAseq through alignment with reference PBMC single cell RNAseq dataset. Colonic data was derived from GSE81861 and PBMC data was acquired from the 10X genomics data portal. (I) Flow cytometry of FcγR expression by human ileal intestinal $CD14^+$ $CX3CR1^+$ macrophages from a healthy donor. Data are representative of two independent experiments. (J) Flow cytometric analysis of total FcγRII-expressing cells within the human ileum. Data are representative of two independent experiments. $P$ values were calculated using a Kruskal-Wallis test with Dunn's multiple comparison test (A), limma with multiple correction using BH (B-D), or the non-parametric Mann-Whitney $U$ test (E). * $P < 0.05$; ** $P < 0.01$; *** $P < 0.001$; **** $P < 0.0001$.

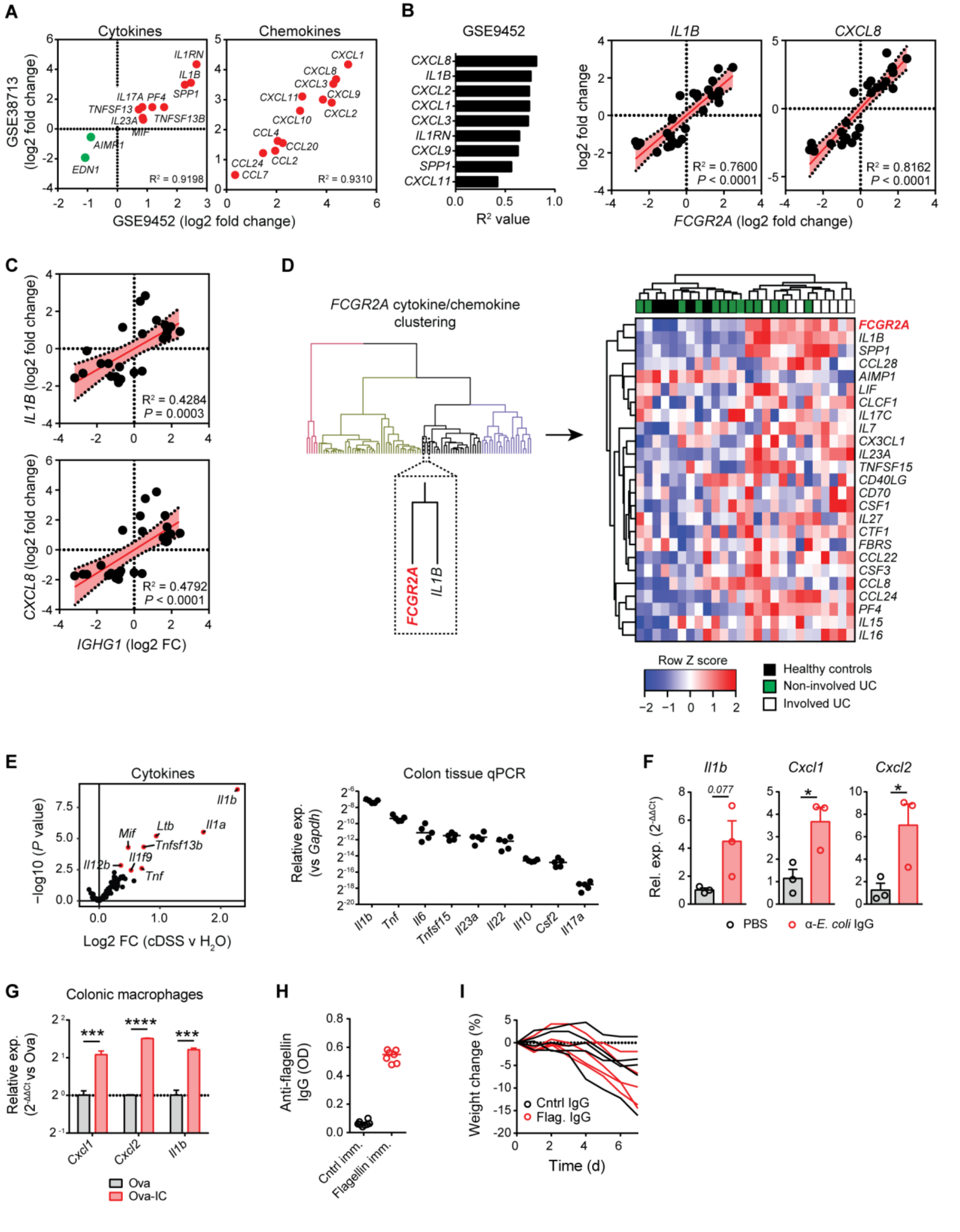


**Figure S3. Mucosal FcγR expression analysis and murine DSS inflammatory networks, related to Figure 3.** (A) Log2 FC comparison of significantly differentially expressed (adj. *P* val. < 0.05) cytokine and chemokine genes across two independent UC cohorts. Data derived from GEO datasets GSE38713 (UC = 15, HC = 13) and GSE9452 (UC = 8, HC = 5). (B) Correlation of *FCGR2A* expression with UC-associated cytokine/chemokine gene transcripts within

pooled mucosal biopsies from data derived from GEO dataset GSE9452. (C) Correlation of *IGHG1* expression with *IL1B* and *CXCL8* gene transcripts in colonic biopsies pooled from healthy controls ($n$ = 5) and UC patients ($n$ = 8). Data were derived from the GEO dataset GSE9452. (D) Hierarchical clustering of chemokine and cytokine genes with *FCGR2A* expression levels in a replication pooled dataset of healthy controls ($n$ = 5), non-inflamed ($n$ = 13) and inflamed UC patients ($n$ = 8) (GEO dataset GSE9452). (E) Volcano plot showing enrichment of murine cytokine gene transcripts in inflamed colonic tissue from cDSS treated mice compared to control tissue (left, $n$ = 5 per group) and qPCR of basal cytokine expression in cDSS colonic tissue (right, $n$ = 5). Transcriptomics data was derived from the GEO dataset GSE42768. Mean ± s.e.m. are indicated. Data are representative of three independent experiments. (F) *Il1b*, *Cxcl1*, and *Cxcl2* expression levels in colonic tissue following 7-day aDSS and 0.5mg anti-*Escherichia coli/Enterobacteriaceae* IgG injection. Data are normalized to PBS control. Means ± s.e.m. are indicated. Data are representative of two independent experiments. (G) qPCR of flow-sorted colonic CX3CR1$^{+}$ Ly6C$^{lo}$ MHC-II$^{+}$ macrophages stimulated for 4h with Ova or Ova-IC ($n$ = 2 per condition). Means ± s.e.m. are indicated. Data are representative of three independent experiments. (H) Serum anti-flagellin IgG titers in mice hyperimmunized with flagellin or PBS in IFA ($n$ = 7 per group). Means ± s.e.m. are indicated. (I) Weight change in *Rag2*$^{-/-}$ mice receiving 0.5 mg anti-flagellin IgG or control IgG and subjected to 7 days of aDSS ($n$ = 5 per group). $P$ values were calculated using limma with multiple correction using BH (A, E), linear regression analysis (B, C), parametric Student's $t$ test (F), or two-way ANOVA with Tukey's multiple comparisons test (G). * $P < 0.05$; *** $P < 0.001$; **** $P < 0.0001$.

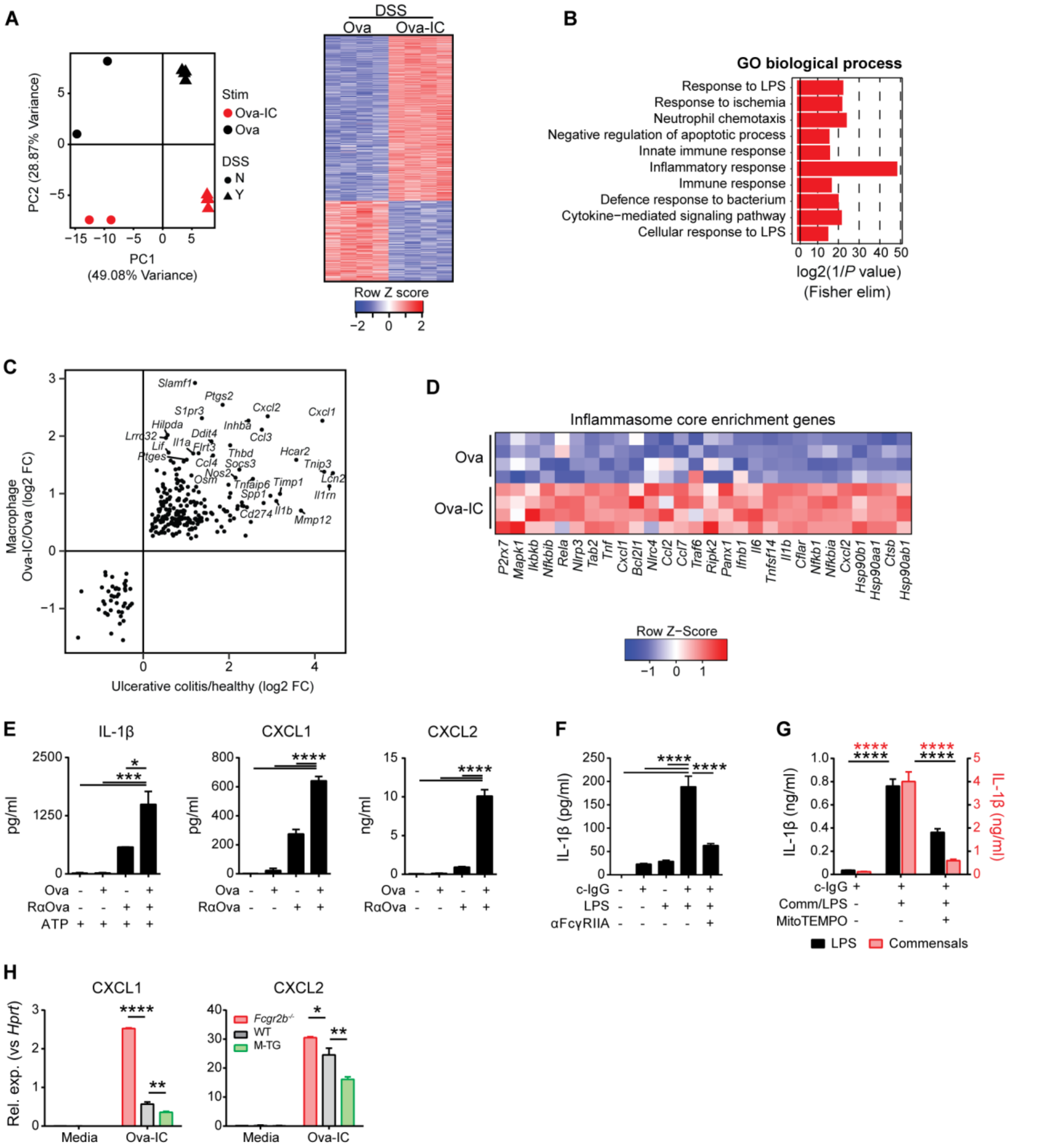

**Figure S4. FcγR signaling analysis on intestinal macrophages, related to Figure 4.** (A) Principal component plot of intestinal macrophages extracted from aDSS or healthy colons stimulated for Ova or Ova-IC for 4 h (left) and heatmap of differential gene expression in aDSS-macrophages (right) ($n$ = 2-4 per condition). (B) Gene ontology analysis of co-regulated genes between UC and Ova-IC-stimulated macrophages using TopGo. (C) Plot of significant co-regulated genes between UC and Ova-IC-stimulated macrophages, as determined by sdef. (D) Core enrichment genes of inflammasome pathways in intestinal macrophages induced by Ova-IC stimulation ($n$ = 4 per condition). (E) Cytokine induction by murine BMDMs stimulated with anti-Ova immune serum alone compared to Ova or Ova-IC for 4h ($n$ = 3 per condition). Mean ± s.e.m. are indicated. Data are representative of three independent experiments. (F) IL-1β production by human MDMs stimulated with LPS and c-IgG for 18h ± anti-FcγRIIA IgG ($n$ = 3 per condition). Mean ± s.e.m. are indicated. Data are representative of three independent experiments. (G) The effect of

mitochondrial ROS inhibitor mitoTEMPO on commensal- and LPS-induced IL-1β production on MDMs stimulated as in F. Mean ± s.e.m. are indicated. Data are representative of two independent experiments. (H) *Cxcl1* and *Cxcl2* induction in murine BMDMs stimulated for 4 h with Ova or Ova-IC ($n$ = 3 per group). Data are representative of two independent experiments. $P$ values were calculated using the standard DESeq 2 method with multiple correction using BH (A-D), one-way ANOVA with Tukey's multiple comparisons test (E-G), or a two-way ANOVA with Tukey's multiple comparisons test (H).

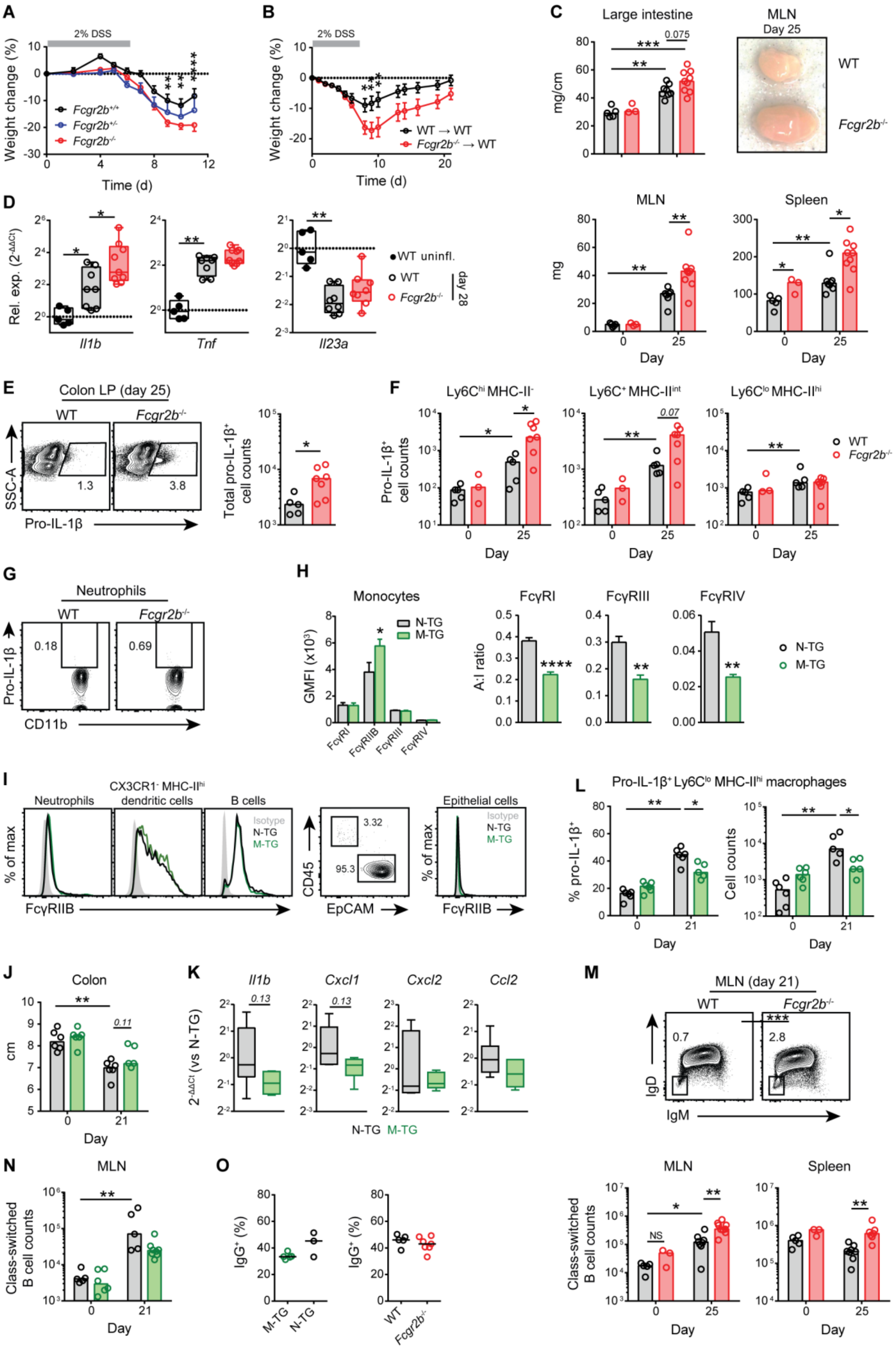
A
2% DSS
Weight change (%)
Time (d)
Fcgr2b+/+
Fcgr2b+/-
Fcgr2b-/-
B
2% DSS
WT → WT
Fcgr2b-/- → WT
C
Large intestine
mg/cm
0.075
MLN
Day 25
WT
Fcgr2b-/-
MLN
Spleen
mg
Day
D
Rel. exp. (2-ΔΔCt)
Il1b
Tnf
Il23a
WT uninfl.
WT
Fcgr2b-/-
day 28
E
Colon LP (day 25)
WT
Fcgr2b-/-
SSC-A
Pro-IL-1β
1.3
3.8
Total pro-IL-1β+ cell counts
F
Ly6Chi MHC-II-
Ly6C+ MHC-IIint
Ly6Clo MHC-IIhi
Pro-IL-1β+ cell counts
0.07
G
Neutrophils
Pro-IL-1β
CD11b
0.18
0.69
H
Monocytes
GMFI (x10³)
N-TG
M-TG
FcγRI
FcγRIIB
FcγRIII
FcγRIV
A:I ratio
I
Neutrophils
CX3CR1- MHC-IIhi dendritic cells
B cells
Isotype
% of max
FcγRIIB
3.32
95.3
CD45
EpCAM
Epithelial cells
L
Pro-IL-1β+ Ly6Clo MHC-IIhi macrophages
% pro-IL-1β+
Cell counts
J
Colon
cm
0.11
K
2-ΔΔCt (vs N-TG)
Cxcl1
Cxcl2
Ccl2
0.13
M
MLN (day 21)
0.7
2.8
IgD
IgM
N
Class-switched B cell counts
O
IgG+ (%)
NS

**Figure S5. MNP FcγR A:I ratio modulates intestinal inflammation, related to Figure 5.** (A, B) Weight loss in *Fcgr2b*$^{+/+}$, *Fcgr2b*$^{+/-}$, and *Fcgr2b*$^{-/-}$ littermate control mice (*n* = 4-6 per group) (A) and WT C57BL/6 mice reconstituted with WT or *Fcgr2b*$^{-/-}$ bone marrow (*n* = 8 per group) (B) following aDSS. Mean ± s.e.m. are indicated. (C) Colon and lymphoid organ weights in WT and *Fcgr2b*-deficient mice at day 25 post-aDSS (*n* = 5-7 per group) or healthy controls (day 0, *n* = 3-5 per group). Medians are indicated. Data are representative of three independent experiments. (D) qPCR of cytokines in whole colonic tissue of WT and *Fcgr2b*$^{-/-}$ mice at day 28 post-aDSS and healthy control colons (*n* = 5-9 per group). Data are normalized to WT uninflamed mRNA levels using the $2^{-\Delta\Delta Ct}$ method and *Gapdh*. Min. to max. box-and-whisker plots are indicated. Data are representative of two independent experiments. (E) Analysis of frequency (left) and absolute cell counts (right) of total pro-IL-1β-expressing cells in WT and *Fcgr2b*$^{-/-}$ mice treated with DSS as in C. For absolute cell counts, medians are indicated. Data are representative of three independent experiments. (F) Quantification of absolute cell counts of colonic pro-IL-1β-expressing waterfall MNP subsets in WT and *Fcgr2b*-deficient mice treated as in C. Medians are indicated. (G) Pro-IL-1β expression by colonic neutrophils in colitic mice treated as in C. (H) FcγR expression analysis and A:I ratio calculation for of colonic monocytes in M-TG and N-TG colons (*n* = 3 per group). Mean ± s.e.m. are indicated. Data are representative of two independent experiments. (I) FcγRIIB expression by colonic leukocytes and intestinal epithelial cells in M-TG and N-TG mice. Data are representative of three independent experiments. (J) Colon length in M-TG and N-TG at day 21 post-aDSS (*n* = 5-6 per group) or in healthy controls (day 0, *n* = 6 per group)). Medians are indicated. Data are representative of three independent experiments. (K) qPCR of cytokines and chemokines in whole colonic tissue from N-TG and M-TG mice treated as in J (*n* = 5 per group). Data are normalized to N-TG mRNA levels using the $2^{-\Delta\Delta Ct}$ method and *Gapdh*. Min. to max. box-and-whisker plots are shown. (L) Frequency of pro-IL-1β expression by colonic Ly6C$^{lo}$ MHC-II$^{hi}$ macrophages (left) and absolute counts of pro-IL-1β-expressing macrophages (right) in M-TG and N-TG mice treated as in J. Medians are indicated. (M) Analysis of the frequency (top) and absolute cell counts (bottom) of class-switched IgM$^{-}$ IgD$^{-}$ B cells in the MLN and spleen of WT and *Fcgr2b*$^{-/-}$ mice at day 21 post-aDSS. Medians are indicated. Data are representative of three independent experiments. (N) Absolute cell counts of class-switched B cells in the MLN of M-TG and N-TG mice treated as in J. Medians are indicated. Data are representative of two independent experiments. (O) IgG binding of day 21 aDSS serum from N-TG/M-TG (left, *n* = 3-5 per group) and WT/*Fcgr2b*$^{-/-}$ mice (right, *n* = 5-7 per group) to *Rag2*-deficient fecal commensals. Medians are indicated. Data are representative of two independent experiments. *P* values were calculated using a two-way ANOVA with Bonferroni's multiple comparisons test (A, B), the nonparametric Mann-Whitney *U* test (C-F, K-O), or Student's two-tailed *t* test (H). * $P < 0.05$; ** $P < 0.01$; *** $P < 0.001$; **** $P < 0.0001$.

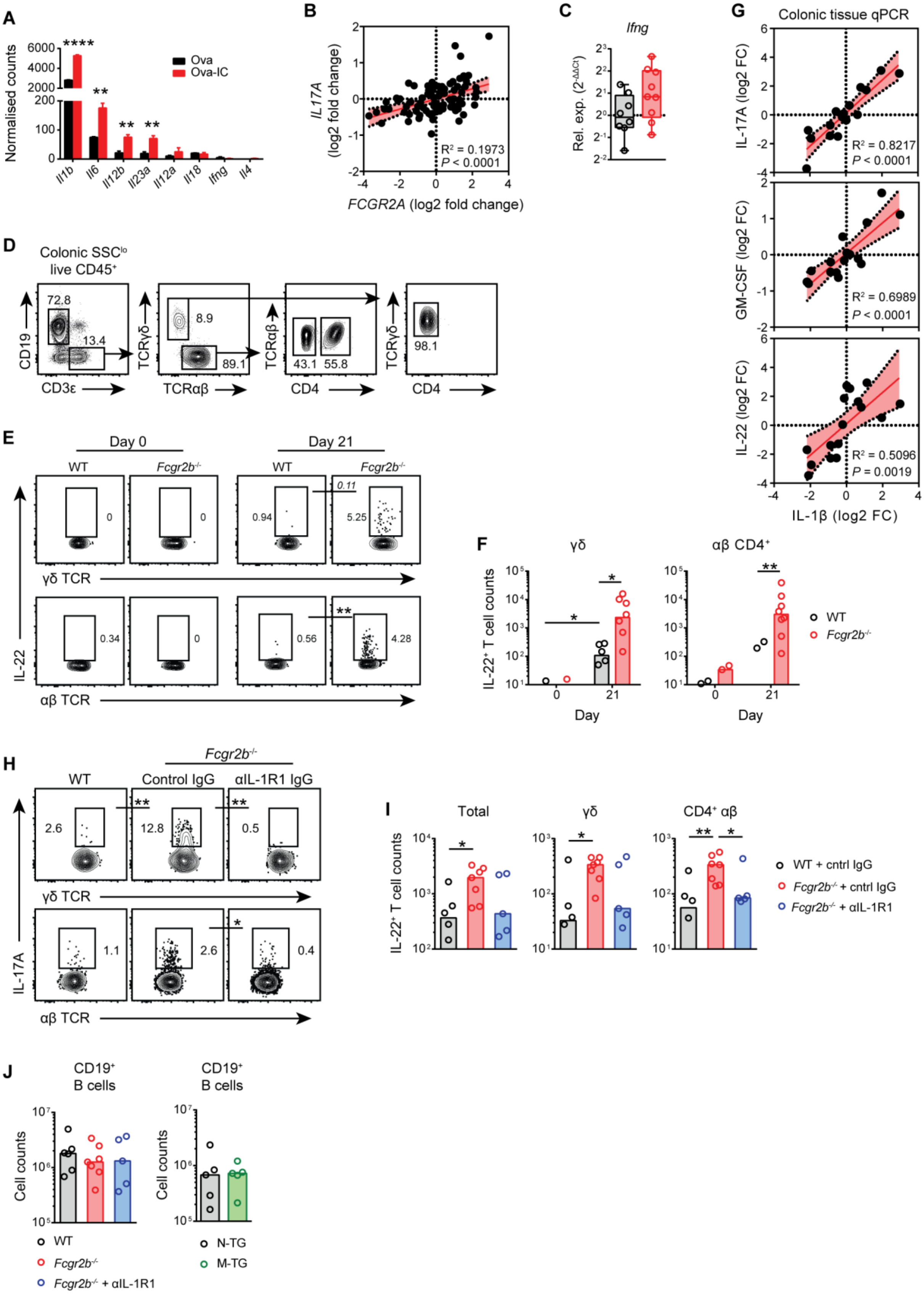
A
Normalised counts
Ova
Ova-IC
Il1b
Il6
Il12b
Il23a
Il12a
Il18
Ifng
Il4
B
IL17A (log2 fold change)
FCGR2A (log2 fold change)
$R^2$ = 0.1973
$P$ < 0.0001
C
Ifng
Rel. exp. ($2^{-\Delta\Delta Ct}$)
G
Colonic tissue qPCR
IL-17A (log2 FC)
$R^2$ = 0.8217
$P$ < 0.0001
GM-CSF (log2 FC)
$R^2$ = 0.6989
$P$ < 0.0001
IL-22 (log2 FC)
$R^2$ = 0.5096
$P$ = 0.0019
IL-1β (log2 FC)
D
Colonic SSC$^{lo}$ live CD45$^+$
CD19
CD3ε
TCRγδ
TCRαβ
CD4
E
Day 0
Day 21
WT
Fcgr2b$^{-/-}$
γδ TCR
αβ TCR
IL-22
F
γδ
αβ CD4$^+$
IL-22$^+$ T cell counts
Day
WT
Fcgr2b$^{-/-}$
H
Fcgr2b$^{-/-}$
WT
Control IgG
αIL-1R1 IgG
γδ TCR
αβ TCR
IL-17A
I
Total
γδ
CD4$^+$ αβ
IL-22$^+$ T cell counts
WT + cntrl IgG
Fcgr2b$^{-/-}$ + cntrl IgG
Fcgr2b$^{-/-}$ + αIL-1R1
J
CD19$^+$ B cells
Cell counts
WT
Fcgr2b$^{-/-}$
Fcgr2b$^{-/-}$ + αIL-1R1
N-TG
M-TG

**Figure S6. MNP FcγR A:I ratio modulates intestinal type 17 immunity, related to Figure 6.** (A) Expression of T cell-inducing cytokines in flow-sorted colonic Ly6C$^{lo}$ MHC-II$^{hi}$ macrophages from colitic mice stimulated with Ova or Ova-IC for 4h ($n$ = 4 per condition). Mean ± s.e.m. are indicated. Data are representative of three independent experiments. (B) Correlation of *FCGR2A* with *IL17A* gene transcripts in colonic biopsies pooled from healthy controls and UC patients ($n$ = 85). Data were derived from GEO dataset GSE59071. (C) qPCR of *Ifng* in whole colonic tissue from WT and *Fcgr2b*$^{-/-}$ mice at day 28 post-aDSS ($n$ = 8-9 per group). Medians are indicated. (D) Flow cytometry gating strategy for quantification of colonic lymphocytes (left). (E, F) Quantification of the frequency (E) and absolute cell counts (F) of colonic IL-22 T cell subsets in WT and *Fcgr2b*$^{-/-}$ mice at day 21 post-aDSS ($n$ = 6-7 per group) or healthy controls (day 0, $n$ = 3-5 per group). For absolute cell counts, medians are indicated. Data are representative of three independent experiments. (G) Correlation of type 17-associated cytokines and IL-1β mRNA levels in whole colonic tissue pooled from WT and *Fcgr2b*$^{-/-}$ colitic mice by qPCR ($n$ = 16). Data are representative of two independent experiments. (H) Frequency of colonic IL-17A-producing T cell subsets in WT and *Fcgr2b*$^{-/-}$ mice at day 15 post-aDSS and treated with control IgG or anti-IL-1R1 IgG blocking antibody ($n$ = 5-7 per group). Data are representative of two independent experiments. (I) Absolute counts of IL-22-producing T cell subsets in inflamed colons of mice treated as in H. (J) Colonic CD19$^{+}$ B cell counts in WT and *Fcgr2b*$^{-/-}$ mice at day 15 post-aDSS plus anti-IL-1R1 or control IgG administration as in H (left, $n$ = 5-7 per group) and N-TG and M-TG mice at day 21 post-aDSS (right, $n$ = 5 per group). Medians are indicated. Data are representative of two or three independent experiments. $P$ values were calculated using the standard DESeq 2 method with multiple correction using BH (A), linear regression analysis (B, G), or nonparametric Mann-Whitney $U$ test (E-J). * $P < 0.05$; ** $P < 0.01$.

**Table S1. Patient characteristics of Cambridge cohort used for microbial flow cytometry, related to Figure 1.**

| Pair # | Sex | Age | Status | Walmsley CAI score | CRP (mg/l) | Medication at sampling |
|---|---|---|---|---|---|---|
| 1 | F | 28 | UC | 12 | 72 | None |
| | M | 28 | HHC | - | - | - |
| 2 | M | 42 | UC | 1 | <4 | Prednisolone<br>Salofalk |
| | F | 37 | HHC | - | - | - |
| 3 | M | 20 | UC | 6 | N/A | Inpatient<br>Co-trimoxazole<br>Cyclosporine<br>Hydrocortisone<br>Ciprofloxacin<br>Metronidazole |
| | F | 55 | HHC | - | - | - |
| 4 | M | 33 | UC | 13 | <4 | Inpatient<br>Methylprednisolone<br>Hydrocortisone |
| | F | 26 | HHC | - | - | - |
| 5 | M | 65 | UC | 5 | N/A | Adcal<br>Asacol<br>Prednisolone |
| | F | 51 | HHC | - | - | - |
| 6 | F | 28 | UC | 5 | <4 | Azathioprine<br>Methylprednisolone |
| | M | 29 | HHC | - | - | |

UC = ulcerative colitis.
HHC = Household healthy control.

**Table S2. Baseline characteristics of infliximab-refractory UC dataset (GSE16879) (Arijs et al., 2009a; Arijs et al., 2009b), related to Figure 2.**

| **Characteristic** | **Responders (*n* = 8)** | **Non-responders (*n* = 16)** |
|---|---|---|
| Male/female (%) | 4/4 (50/50) | 10/6 (62.5/37.5) |
| Median (IQR) age at first IFX (years) | 28.4 (24.3-41.8) | 45.8 (36.5-62.3) |
| Median (IQR) weight at first IFX (kg) | 72 (57.8-78.5) | 73.3 (68.5-80.3) |
| Median (IQR) duration of disease prior to first IFX (years) | 10.3 (4.1-17.3) | 7.3 (2.6-13.3) |
| Median (IQR) C-reactive protein at first IFX (mg/dl) | 1.65 (1-9.6) | 6.5 (2.9-19.1) |
| Median (IQR) Mayo score before first IFX | 10 (8.8-10) | 9.5 (8.8-10.3) |
| Concomitant medication at first IFX (%) | | |
| 5-Aminosalicylates | 5 (62.5) | 13 (81.3) |
| Corticosteroids | 2 (25) | 5 (31.3) |
| Azathioprine/6-mercaptopurine | 7 (87.5) | 8 (50) |
| Corticosteroids + immunosuppressants | 2 (25) | 1 (6.3) |
| Active smoking at first IFX (%) | 1 (12.5) | 1 (6.3) |
