# Supplementary material for: Anti-commensal IgG Drives Intestinal Inflammation and Type 17 Immunity in Ulcerative Colitis
Source: Immunity. 2019 Apr 16;50(4):1099–1114.e10. doi: 10.1016/j.immuni.2019.02.006 (PMC6477154; doi:10.1016/j.immuni.2019.02.006)
Supplement: Document S2. Article plus Supplemental Information [file mmc2.pdf]

Immunity

Article

Anti-commensal IgG Drives Intestinal Inflammation and Type 17 Immunity in Ulcerative Colitis

Graphical Abstract

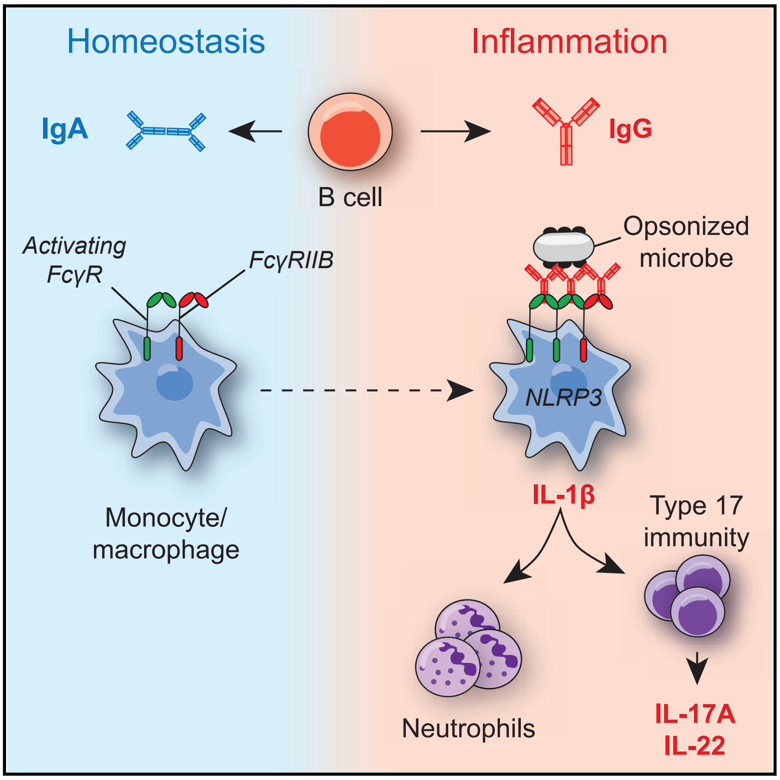

Highlights

- Intestinal inflammation in UC is associated with increased anti-commensal IgG
- Commensal-IgG cross-link FcγR on colonic MNPs, inducing IL-1β production
- MNP FcγR A:I ratio determines magnitude of type 17 immunity and local inflammation
- Identifies cellular mechanisms by which FcγRIIA H/R131 confers UC susceptibility

Authors

Tomas Castro-Dopico, Thomas W. Dennison, John R. Ferdinand, ..., Simon Clare, Miles Parkes, Menna R. Clatworthy

Correspondence

mrc38@cam.ac.uk

In Brief

Castro-Dopico et al. find a profound induction of anti-commensal IgG in the colonic mucosa of UC patients and outline a pathway whereby FcγR receptor activation by IgG leads to IL-1β production, type 17 immunity, and the exacerbation of inflammation. Their findings reveal an important contribution of IgG-mediated inflammation in an IgA-dominated organ.

Castro-Dopico et al., 2019, Immunity *50*, 1099–1114
April 16, 2019 © 2019 The Authors. Published by Elsevier Inc.
https://doi.org/10.1016/j.immuni.2019.02.006

CellPress

Anti-commensal IgG Drives Intestinal Inflammation and Type 17 Immunity in Ulcerative Colitis

Tomas Castro-Dopico,[1] Thomas W. Dennison,[1] John R. Ferdinand,[1] Rebeccah J. Mathews,[1] Aaron Fleming,[1] Dean Clift,[2] Benjamin J. Stewart,[1] Chenzhi Jing,[1] Konstantina Strongili,[3] Larisa I. Labzin,[2] Edward J.M. Monk,[1] Kourosh Saeb-Parsy,[4] Clare E. Bryant,[5] Simon Clare,[6] Miles Parkes,[3] and Menna R. Clatworthy[1,7,8,*]
[1]Molecular Immunity Unit, University of Cambridge Department of Medicine, Cambridge CB2 0QH, UK
[2]Medical Research Council, Laboratory of Molecular Biology, Cambridge CB2 0QH, UK
[3]Division of Gastroenterology, Cambridge Universities NHS Foundation Trust, Cambridge CB2 0QQ, UK
[4]University of Cambridge Department of Surgery, Cambridge CB2 0QQ, UK
[5]Department of Veterinary Medicine, University of Cambridge, Cambridge CB3 0ES, UK
[6]Wellcome Trust Sanger Institute, Wellcome Trust Genome Campus, Hinxton CB10 1SA, UK
[7]Cellular Genetics, Wellcome Trust Sanger Institute, Wellcome Trust Genome Campus, Hinton CB10 1SA, UK
[8]Lead Contact
*Correspondence: mrc38@cam.ac.uk
https://doi.org/10.1016/j.immuni.2019.02.006

SUMMARY

Inflammatory bowel disease is a chronic, relapsing condition with two subtypes, Crohn's disease (CD) and ulcerative colitis (UC). Genome-wide association studies (GWASs) in UC implicate a *FCGR2A* variant that alters the binding affinity of the antibody receptor it encodes, FcγRIIA, for immunoglobulin G (IgG). Here, we aimed to understand the mechanisms whereby changes in FcγRIIA affinity would affect inflammation in an IgA-dominated organ. We found a profound induction of anti-commensal IgG and a concomitant increase in activating FcγR signaling in the colonic mucosa of UC patients. Commensal-IgG immune complexes engaged gut-resident FcγR-expressing macrophages, inducing NLRP3- and reactive-oxygen-species-dependent production of interleukin-1β (IL-1β) and neutrophil-recruiting chemokines. These responses were modulated by the *FCGR2A* genotype. *In vivo* manipulation of macrophage FcγR signal strength in a mouse model of UC determined the magnitude of intestinal inflammation and IL-1β-dependent type 17 immunity. The identification of an important contribution of IgG-FcγR-dependent inflammation to UC has therapeutic implications.

INTRODUCTION

Inflammatory bowel disease (IBD) is a chronic, relapsing condition with two main clinicopathological subtypes, Crohn's disease (CD) and ulcerative colitis (UC) (Kaser et al., 2010). Susceptibility to IBD is driven by a genetic predisposition to aberrant mucosal responses to commensals (Jostins et al., 2012; McGovern et al., 2015; Neurath, 2014) and characterized by the inappropriate production of a number of pro-inflammatory cytokines and chemokines (Neurath, 2014). Genome-wide association studies (GWASs) have provided critical insights into disease pathogenesis and implicate a *FCGR2A* variant that alters the binding affinity of the antibody receptor it encodes (Jostins et al., 2012). Fc gamma receptors (FcγRs) bind to the Fc portion of immunoglobulin G (IgG), are expressed by many immune cells (including macrophages), and mediate the cellular effector functions of IgG antibodies. These cell-surface glycoproteins include activating receptors (in humans FcγRIIA, IIIA, and IIIB) and a single inhibitory receptor FcγRIIB (Nimmerjahn and Ravetch, 2008; Smith and Clatworthy, 2010). The extent to which IgG immune complexes (ICs) activate immune cells is dependent on the relative engagement of activating or inhibitory FcγRs (the A:I ratio).

Genetic variation in FcγRs can alter the A:I ratio and influence susceptibility to a number of autoimmune diseases (Smith and Clatworthy, 2010). An *FCGR2A* single-nucleotide polymorphism (SNP) (dbSNP: rs1801274) leading to an amino acid substitution (histidine to arginine at position 131) results in a lower binding affinity for IgG, reducing the A:I ratio (Willcocks et al., 2009). FcγRIIA-R131 is protective in UC (Jostins et al., 2012), suggesting that IgG might play a pathogenic role in intestinal inflammation. Although *in vitro* studies have identified a generic increase in the production of a number of pro-inflammatory cytokines by immune cells in response to IgG (Uo et al., 2013), there is limited insight into the cellular pathways underpinning this genetic association. Furthermore, IgG antibodies are considered less important in intestinal immunity because of the dominance of IgA at mucosal surfaces (Fagarasan, 2008).

We found a profound induction of anti-commensal IgG and of activating FcγR signaling in the colonic mucosa in UC patients. The resulting commensal-IgG immune complexes engaged gut-resident FcγR-expressing macrophages, inducing NLRP3- and reactive oxygen species (ROS)-dependent production of IL-1β and neutrophil-recruiting chemokines, and this was modulated by *FCGR2A* genotype. In a murine model of intestinal inflammation, manipulation of macrophage FcγR signal strength determined the magnitude of intestinal inflammation and of IL-1β-dependent induction of type 17 immunity *in vivo*. Our findings provide insight into the mechanisms mediating IgG-FcγR-associated inflammation in UC, which might present therapeutic targets for the treatment of UC.

This is an open access article under the CC BY license (http://creativecommons.org/licenses/by/4.0/).

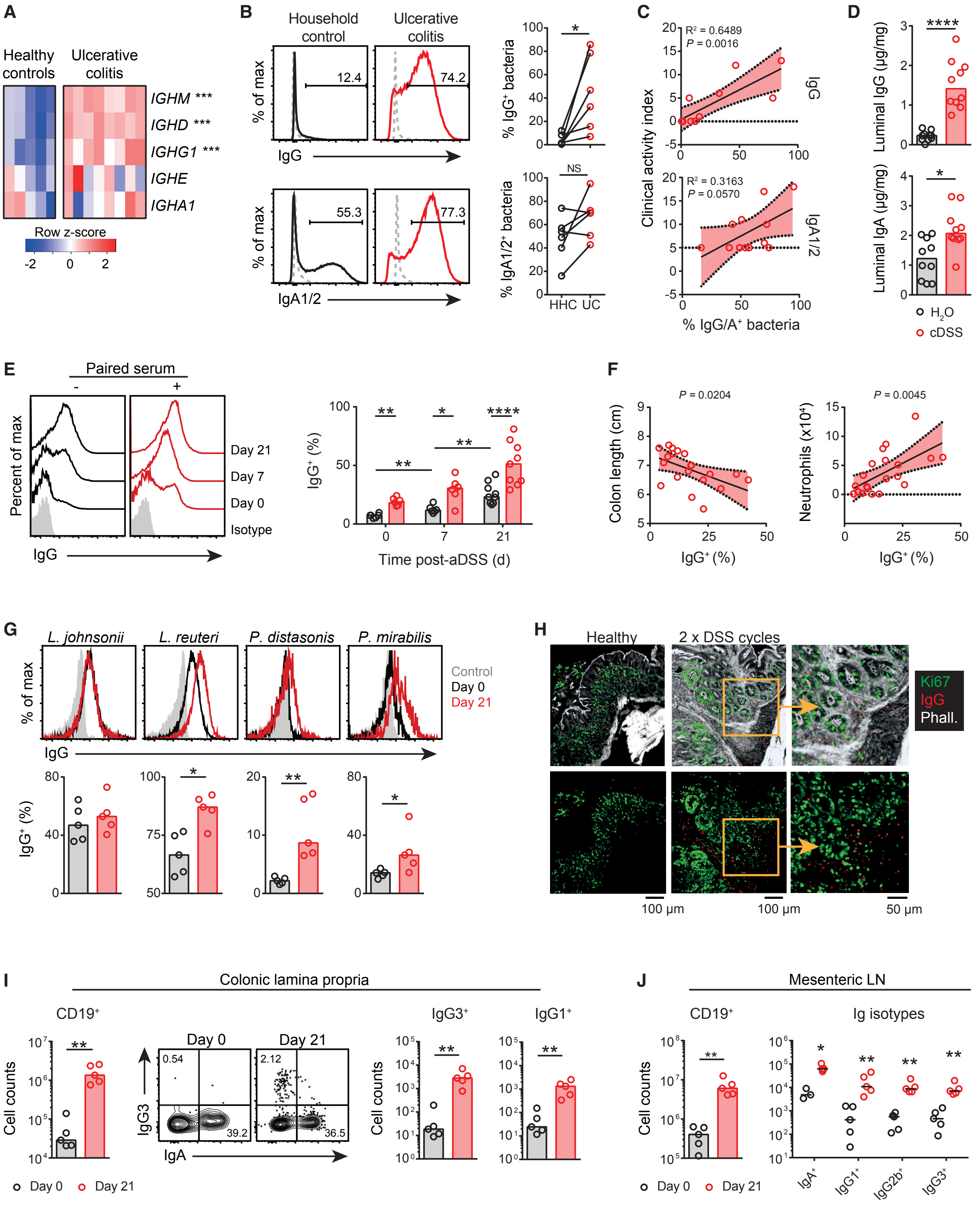

Figure 1. Anti-commensal IgG Is Associated with the Magnitude of Intestinal Inflammation

(A) Analysis of human Ig heavy-chain gene transcripts in healthy and UC colonic biopsies. Data were derived from Gene Expression Omnibus (GEO) dataset GEO: GSE9452.

(legend continued on next page)

RESULTS

Anti-commensal IgG Is Associated with the Magnitude of Intestinal Inflammation

We first sought to interrogate whether there was an increase in local IgG within the gastrointestinal (GI) tract during intestinal inflammation, in line with previous reports (Baklien and Brandtzaeg, 1975; Macpherson et al., 1996; Shafir et al., 1986; Uo et al., 2013). Analysis of published transcriptomic data of intestinal biopsies from a cohort of patients with clinically active UC demonstrated a significant enrichment of IgM and IgG heavy-chain transcripts within inflamed mucosa (Figures 1A and S1A). Indeed, upregulation of an *IGH* signature (a cumulative measure of *IGH* expression) was specifically associated with diseased tissue in UC compared with non-diseased UC and healthy control colonic tissue (Figure S1B), implicating humoral responses in disease. Consistent with an increase in local commensal-specific IgG, we observed a significantly higher proportion of luminal commensals bound by IgG in UC stool samples than in household controls, in contrast to IgA-bound microbes (Figure 1B and Table S1). Notably, samples with higher levels of IgG-bound commensals were found in patients with the highest disease severity scores (Figure 1C).

To probe this phenomenon further, we used dextran sodium sulfate (DSS), a colitogen that leads to osmotic epithelial damage, to induce barrier-breach-associated inflammation. Exposure of mice to two repeated cycles of DSS (referred to here as chronic DSS [cDSS]) induced an upregulation of IgG transcripts within the colonic mucosa (Figure S1C), an increase in luminal IgG (Figure 1D), and a modest increase in IgA, mirroring observations in human UC. Indeed, a single acute 6-day course of 2% DSS (referred to here as acute DSS [aDSS]) was sufficient to induce an increase in *de novo* circulating anti-commensal and anti-flagellin IgG, consistent with previous reports (Kobayashi et al., 2009) (Figure S1D). We also observed significantly more luminal microbes bound by IgG at day 7 and day 21 after aDSS exposure than at day 0, and the addition of paired DSS serum further increased bacterial binding by IgG (Figure 1E), whereas there was no significant increase in IgA-bound bacteria (Figure S1E). The number of luminal IgG-bound microbes correlated with colon length and colonic neutrophil infiltration (Figure 1F), which are measures of disease severity. Indeed, the level of IgG-opsonized microbes better reflected colitis severity than that of IgA opsonization (Figure S1F), implicating anti-commensal IgG as a determinant of the intestinal inflammatory response. Circulating anti-commensal IgG titers in serum correlated with colonic neutrophil infiltration at day 21 after aDSS exposure (Figure S1G). This serum IgG response was not specific to a single commensal or pathobiont but exhibited broad microbial specificity, including the probiotic *Lactobacillus reuteri* and the pathobiont *Proteus mirabilis* (Figure 1G), and *L. reuteri* IgG binding correlated most strongly with disease activity of those species tested (Figure S1H). Consistent with an expansion of local IgG production, we observed an increase in IgG-expressing cells within the inflamed submucosa of the colon of cDSS-treated mice (Figure 1H) and that B cells represented the major lymphocyte population in this setting (Figure S1I). By day 21 after aDSS exposure, there was a pronounced expansion of $IgG^{+}CD19^{+}$ B cells within the inflamed colonic lamina propria (LP) (Figures 1I and S1J) and colon-draining mesenteric lymph nodes (MLNs) (Figures 1J and S1M). We also observed an increase in $CD138^{+}$ plasma cells within the inflamed colon (Figure S1K) and MLNs (Figure S1N), as well as an increase in the absolute numbers of IgG-expressing plasma cell at both sites (Figures S1L and S1O), indicating that although the transudation of systemic IgG might make a substantial contribution to the luminal commensal-reactive IgG, there is also *de novo* IgG generation locally within the GI tract. Together, these data suggest that in UC there is an increase in intestinal commensal-specific IgG, positioned to activate FcγR-expressing mucosal immune cells, and that DSS colitis provides a reasonable model for interrogating the effects of local IgG in intestinal inflammation.

Activating FcγR Signaling in Intestinal Inflammation

To address the role of FcγR signaling in intestinal inflammation, we analyzed FcγR mRNA expression in mucosal biopsies in published cohorts of UC patients. Activating FcγR gene transcripts, including *FCGR2A* and *FCGR3A/B*, were among the most differentially expressed genes in inflamed UC biopsies compared with UC in remission and non-inflamed UC biopsies and healthy control biopsies (log2 fold change [FC] > 2; Figures 2A and 2B). In contrast, the inhibitory receptor *FCGR2B* was more modestly induced (Figure 2B), resulting in an increase in the mucosal FcγR A:I ratio (Figure S2A). UC-associated

(B) IgG- and IgA1- and IgA2-bound SYBR green^{hi} microbes in UC and household healthy control (HHC) stool samples were analyzed as household pairs (n = 6 per group).

(C) Correlation of pooled IgG- and IgA1- and IgA2-bound bacterial levels with clinical activity index (CAI) (n = 12).

(D) Murine colon luminal IgG and IgA levels following two cycles of DSS administration (cDSS) and normalized to total protein content (n = 10 per group). Medians are indicated.

(E) Quantification of IgG-bound bacteria in stool after a single acute course of 6-day 2% DSS administration (aDSS) or H_2O with (red) and without (black) paired serum pre-incubation (n = 6–9 per group). Medians are indicated.

(F) Correlation of IgG-bound commensals (no serum) from pooled control and colitic mice at day 21 after aDSS with markers of colonic inflammation; length and neutrophil count (n = 20) are shown. In (D)–(F), data are pooled from two independent experiments.

(G) Opsonization of commensal bacterial species with day 21 aDSS serum or healthy control serum (day 0) (n = 5 per group). Medians are indicated. Data are representative of two independent experiments.

(H) Confocal image of control or inflamed colons from cDSS-treated mice (red, IgG; green, Ki67; white, phalloidin). Data representative of three independent experiments.

(I and J) Quantification of IgG- and IgA-expressing IgM^{-} B cell subsets in murine colons (I) and colon-draining MLN (J) at day 21 after aDSS administration versus controls (day 0) (n = 5 per group). For absolute cell-count quantification plots, medians are indicated. Data are representative of three independent experiments.

p values were calculated via limma with multiple correction using the Benjamini-Hochberg (BH) procedure (A), ratio paired t test (B), linear regression analysis (C and F), or the nonparametric Mann-Whitney U test (D, E, G, I, and J). *p < 0.05, **p < 0.01, ***p < 0.001, ****p < 0.0001. See also Figure S1 and Table S1.

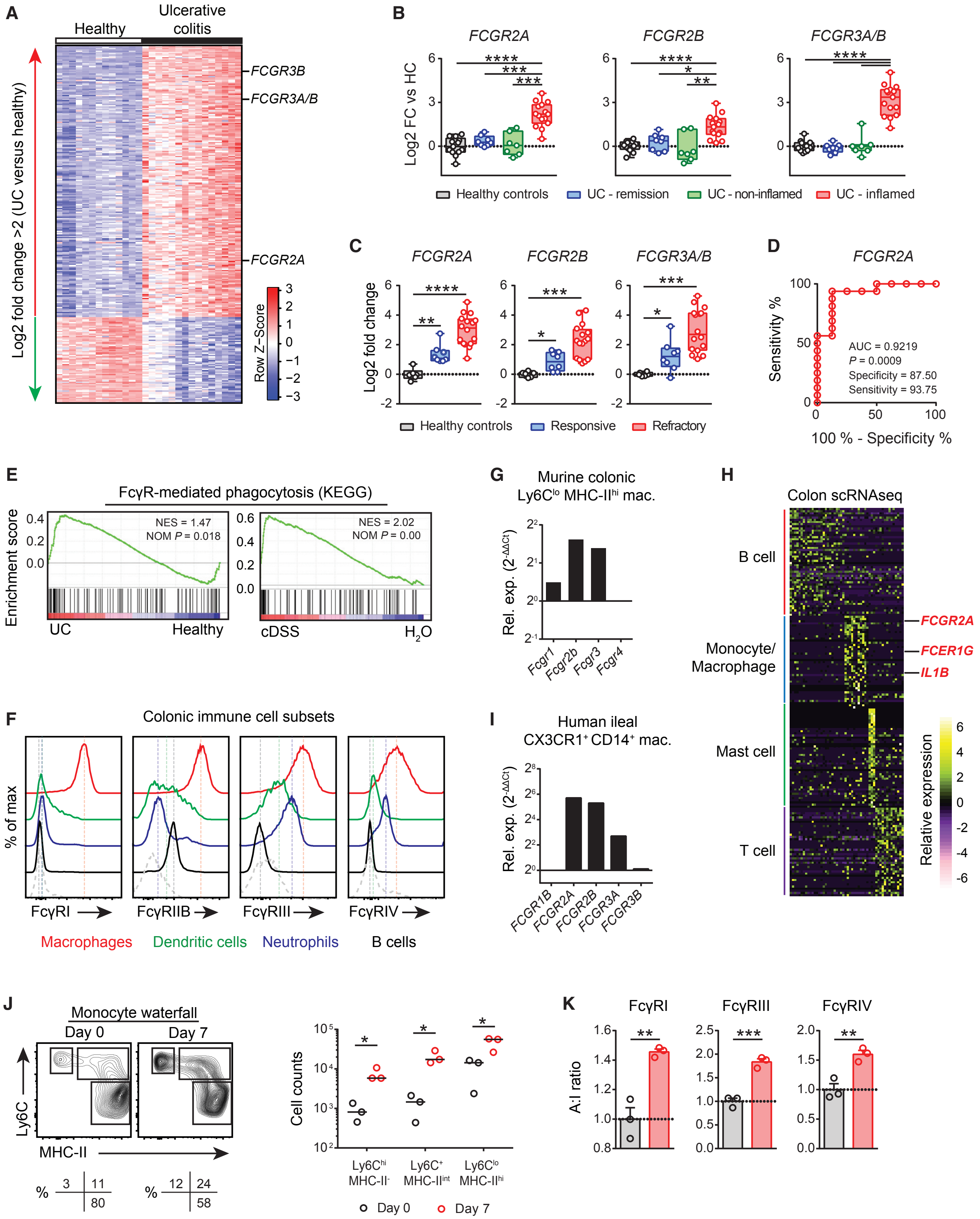

Figure 2. Activating FcγR Signaling in Intestinal Inflammation

(A) Analysis of the top significantly differentially expressed genes (log2 FC > 2) in human active UC mucosal biopsies compared with controls. Data are from GEO: GSE38713.

(legend continued on next page)

enrichment was specific to FcγRs and was not observed for other Fc receptors (Figure S2B), and *FCGR* enrichment was confirmed in a further independent UC dataset (Figure S2C). Furthermore, in mucosal UC biopsies taken prior to infliximab intervention (Table S2), elevated *FCGR2A* expression was associated with treatment-refractory disease (Figure 2C) and was predictive of subsequent resistance to tumor necrosis factor-α (TNF-α) blockade (Arijs et al., 2009a; Arijs et al., 2009b) (Figure 2D).

The functional effect of a higher A:I ratio at a cellular level is to reduce the activation threshold, increasing the likelihood of activation upon IgG IC encounter. In keeping with this, we found an enrichment of genes associated with FcγR-mediated phagocytosis in UC biopsies compared with control biopsies (Figure 2E), a unique pathway distinct from TLR-signaling genes (Figure S2D). Using both a published transcriptomics dataset and confirmatory qPCR, analysis of colonic FcγR expression in cDSS-treated mice demonstrated a similar increase in FcγR transcripts (Figure S2E) and enrichment of FcγR activation pathway genes (Figure 2E). In contrast, a consistent enrichment of FcγR genes or FcγR signaling pathways in *Citrobacter rodentium* infection was not observed (Figure S2F), suggesting that our observations might be specific to DSS-induced colitis.

A number of immune cell subsets, including macrophages, dendritic cells (DCs), and neutrophils, express FcγRs. During aDSS-induced inflammation, when compared with other colonic-resident immune cells (as identified in Figure S2G), intestinal macrophages ($CD11b^+CX3CR1^+Ly6C^{lo}MHC\text{-}II^+F4/80^+$) expressed the highest levels of both activating and inhibitory FcγRs (Figure 2F). In particular, FcγRIII (the murine ortholog of FcγRIIA) was highly expressed at both the transcript and protein levels (Figures 2F and 2G). To investigate FcγR expression in the human intestine, we made use of a recently published single-cell RNA sequencing (scRNA-seq) dataset generated from human colon. Immune cell subsets were identified through canonical-marker gene expression and alignment with human peripheral-blood mononuclear cell (PBMC) single-cell transcriptomic data (Figure S2H). Analysis of colonic single-cell transcriptomes indicated that *FCGR2A* and *FCER1G* were highly specific to the mononuclear phagocyte (MNP) cluster (Figure 2H), transcriptionally similar to circulating $CD14^+$ classical monocytes (Figure S2H). Indeed, FcγRIIA was confirmed as the dominant FcγR expressed by $CD14^+CX3CR1^+$ macrophages flow sorted from the human intestine (Figures 2I, S2I, and S2J). The increase in activating *FCGR2A* transcripts observed in colonic biopsies in UC might be due to an increase in the number of FcγR-expressing macrophages during inflammation or the upregulation of activating FcγR expression on resident macrophages. The adult intestinal macrophage pool is largely derived from circulating monocytes in homeostasis and inflammation (Bain et al., 2014). Analysis of the $CD11b^+CX3CR1^+$ "monocyte waterfall" (Tamoutounour et al., 2012) demonstrated an influx of colonic monocytes and an increase in newly differentiated inflammatory $Ly6C^+MHC\text{-}II^{int}$ macrophages and mature $Ly6C^{lo}MHC\text{-}II^{hi}$ macrophages at day 7 after aDSS (Figure 2J). Furthermore, activating FcγR expression was augmented on these cells, increasing their A:I ratio (Figure 2K) and rendering them more susceptible to IgG-mediated activation. Together, these data show that the intestinal MNP system is primed to respond to the emergence of local IgG after the onset of intestinal inflammation.

Mucosal FcγR Expression Correlates with IL-1β and CXCL8

To understand the specific molecular pathways that mediate FcγR-induced intestinal inflammation in UC, we first sought to identify common inflammatory networks present in colonic biopsies across multiple UC cohorts and then to interrogate correlations with *FCGR2A*. Analysis of significantly enriched cytokines and chemokines (adjusted p value < 0.05) within inflamed UC mucosal biopsies revealed that *IL1B* and neutrophil-recruiting chemokines, including *CXCL1* and *CXCL8*, were among the most significantly upregulated genes compared with those within controls (Figures 3A and S3A). Of these genes, *IL1B* and *CXCL8* correlated most strongly with *FCGR2A* (Figures 3B, 3C, and S3B). We also observed a correlation between these genes and *IGHG1* expression (Figure S3C). To further examine gene expression associations in an unbiased manner, we performed

(B) *FCGR* enrichment in inflamed active UC (n = 15) compared with healthy control (n = 13), remission (n = 8), and non-inflamed mucosal biopsies (n = 7). Data are from GEO: GSE38713.

(C) *FCGR* expression in colonic biopsies from UC patients refractory (n = 16) or responsive (n = 8) to infliximab treatment and healthy controls prior to treatment initiation (n = 6). Data are from GEO: GSE16879. Minimum to maximum box-and-whisker plots are shown in (B) and (C).

(D) Area under the receiver operator curve (AUROC) analysis for *FCGR2A* expression in infliximab-refractory mucosal biopsies compared with infliximab-responsive biopsies. Data were derived as in (C).

(E) Gene-set enrichment analysis (GSEA) of the KEGG FcγR-mediated phagocytosis pathway in UC and healthy colonic biopsies (left) and cDSS-inflamed and healthy murine colons (bottom). Data are from GEO: GSE38713 (UC = 15, HC = 13) and GEO: GSE42768 (cDSS = 5, H_2O = 5).

(F) FcγR expression by murine colonic leukocytes at day 7 after aDSS. Data are representative of 3 independent experiments.

(G) qPCR of FcγR mRNA expression in flow-sorted murine colonic $CX3CR1^+Ly6C^{lo}MHC\text{-}II^{hi}$ macrophages, as identified in (F). Data are representative of 2 independent experiments.

(H) Single-cell RNA-seq of immune cell subsets in healthy human colon. Data are from GEO: GSE81861. The heatmap illustrates the top 50 cell-type-specific markers ranked by AUROC score.

(I) qPCR of FcγR mRNA expression in flow-sorted human ileal $CD14^+CX3CR1^+SSC^{int}$ macrophages.

(J) Colonic "monocyte waterfall" subset quantification by flow cytometry at day 7 after aDSS versus controls (n = 3 per group). Flow plots of $CD11b^+CX3CR1^+$ waterfall subsets (left) and quantification of absolute cell counts for the indicated subsets (right) are shown. For absolute cell counts, medians are indicated. Data are representative of 3 independent experiments.

(K) FcγR quantification of $CX3CR1^+Ly6C^{lo}MHC\text{-}II^{hi}$ macrophages, as shown in (J), by flow cytometry. Means ± SEM are indicated and normalized to the A:I ratio of control-treated mice.

Data are representative of two independent experiments. p values were calculated via limma with multiple correction using the BH procedure (A–C), AUROC analysis (D and H), or Student's two-tailed t test (J and K). *p < 0.05, **p < 0.01, ***p < 0.001, ****p < 0.0001. See also Figure S2 and Table S2.

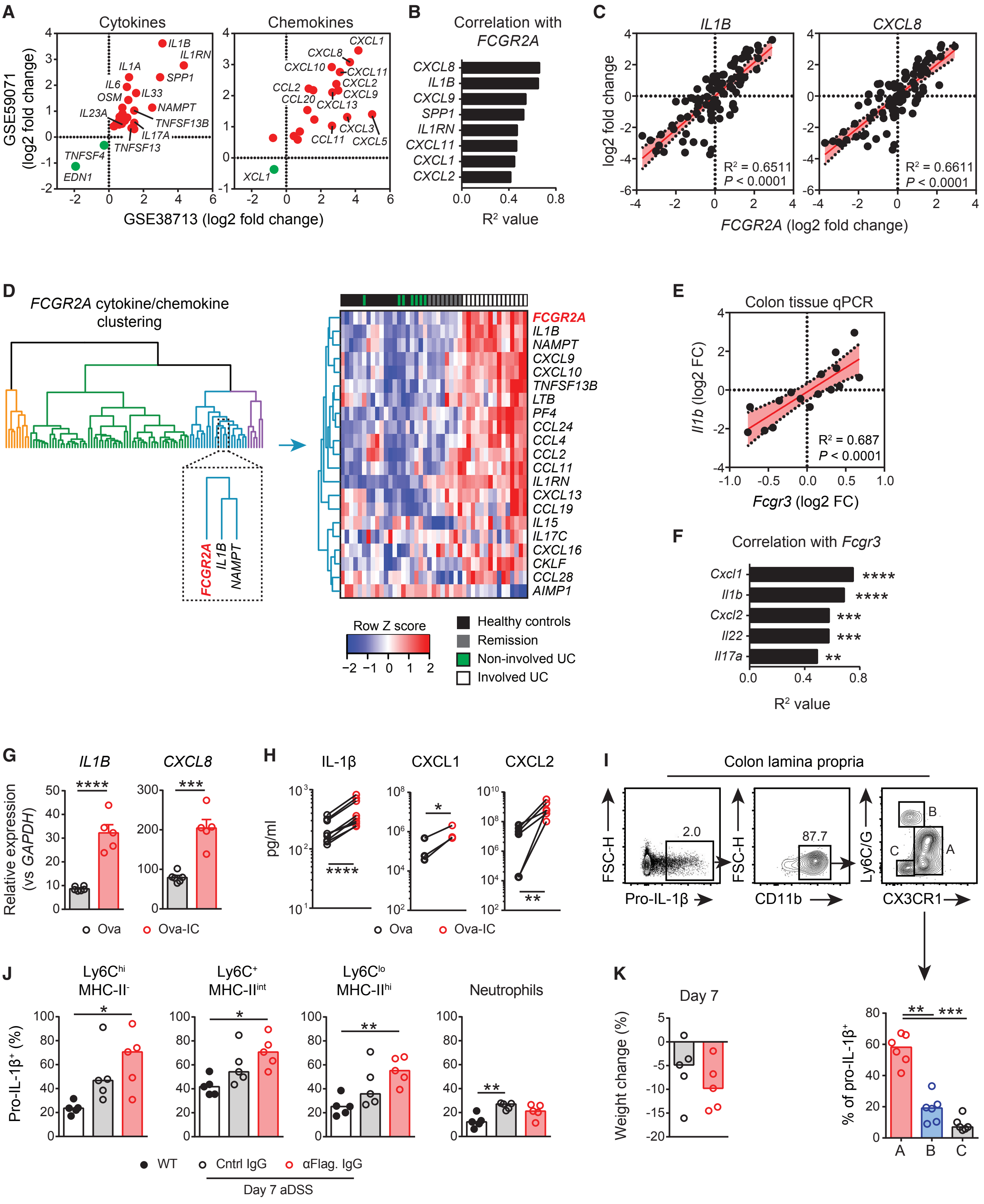

Figure 3. Mucosal FcγR Expression Correlates with IL-1β and CXCL8

(A) Log2 fold-change comparison of differentially expressed cytokine and chemokine genes across two independent UC cohorts. Data are from GEO: GSE38713 (UC = 15, HC = 13) and GEO: GSE59071 (UC = 74, HC = 11).

(legend continued on next page)

hierarchical clustering of all chemokine and cytokine gene transcripts and *FCGR2A* within these same cohorts. Strikingly, this placed *IL1B* in a cluster with, and adjacent to, *FCGR2A* (Figures 3D and S3D). In cDSS colitis, as in UC biopsies, *Il1b* was the most highly induced cytokine gene within the inflamed colon, as assessed by transcriptomic analysis, and displayed higher total expression than did other inflammatory mediators, as shown by qPCR, suggesting a dominant role for this cytokine in colitis (Figure S3E). Furthermore, its expression closely correlated with *Fcgr3* transcript expression within whole colonic tissue by qPCR (Figure 3E), as did *Cxcl1* and *Cxcl2* (murine neutrophil-recruiting chemokines; Figure 3F). Together, these data demonstrate that the expression of FcγRIIA and IL-1β are closely associated during colitis and raise the possibility that anti-commensal IgG might contribute to intestinal inflammation via FcγR-dependent induction of IL-1β, a Th17-polarizing cytokine (Chung et al., 2009; Shaw et al., 2012), and neutrophil-recruiting chemokines.

To determine whether IgG and activating FcγR signaling and these key cytokines and chemokines might be causatively linked, we isolated human gut LP mononuclear cells (LPMCs) and stimulated them with IgG IC *ex vivo* to directly cross-link FcγRs. This resulted in a significant induction of *IL1B* and *CXCL8* (Figure 3G). Similarly, *ex vivo* stimulation of murine LPMCs from aDSS-treated mice with IgG ICs resulted in an increase in IL-1β, CXCL1, and CXCL2 production (Figure 3H). *In vivo*, passive transfer of anti-commensal IgG further enhanced *Il1b*, *Cxcl1*, and *Cxcl2* transcript levels within the colon after 7-day aDSS (Figure S3F). Together, these data support the hypothesis that commensal IgG directly drives intestinal inflammation via induction of pro-inflammatory cytokines and chemokines on resident intestinal immune cells. Intracellular cytokine staining of murine LPMCs demonstrated that $CD11b^{+}CX3CR1^{+}$ cells (population A), comprising intestinal mononuclear phagocytes (MNPs), were the dominant source of IL-1β during colitis, with a minor contribution from neutrophils (Figure 3I), consistent with our analysis of human colonic scRNA-seq data, showing that *IL1B* expression is specific to the MNP cluster (Figure 2H). Indeed, flow-sorted murine colonic macrophages stimulated with IgG ICs *ex vivo* demonstrated substantial induction of IL-1β, CXCL1, and CXCL2 expression (Figure S3G). Finally, we immunized and boosted C57BL/6 mice to generate high titers of circulating anti-flagellin IgG (Figure S3H). Transfer of this anti-flagellin-enriched serum IgG into naive IgG-deficient *Rag2*$^{-/-}$ mice prior to aDSS significantly augmented pro-IL-1β expression by colonic $CX3CR1^{+}$ MNP subsets, particularly $Ly6C^{hi}$ monocytes and $Ly6C^{+}MHC\text{-}II^{int}$ inflammatory macrophages (Figure 3J) and was associated with increased weight loss (Figures 3K and S3I). In contrast, no difference in colonic neutrophil pro-IL-1β expression was observed, suggesting that FcγR signaling drives pro-IL-1β expression by colonic MNPs and is sufficient to induce inflammation in the absence of adaptive immune cells.

IgG-Induced IL-1β Production by Human Macrophages

We next sought to further interrogate the role of FcγR signaling in intestinal macrophage function. A cell-intrinsic effect of IgG ICs on macrophage, cytokine, and chemokine production was evident as FcγR cross-linking on flow-sorted murine $CX3CR1^{+}Ly6C^{lo}MHC\text{-}II^{hi}$ colonic macrophages induced widespread changes in gene expression (Figure S4A), including 264 genes that were significantly differentially expressed in UC biopsies (Figure 4A). IC-induced genes were enriched in UC-relevant immune pathways, such as neutrophil chemotaxis (Figure S4B), and included key UC-associated cytokines such as *Il1b*, *Il23a*, and the recently characterized *Osm* (West et al., 2017) (Figures 4A and S4C). Given our previous analyses demonstrating the predominance of *IL1B* expression in inflamed UC biopsies, that its expression is closely linked to that of *FCGR2A*, and that *IL1B* is induced in lamina propria MNP after FcγR engagement (Figure 3), we sought to determine the molecular pathway linking FcγR cross-linking and IL-1β production. FcγR signaling resulted in the induction of genes involved in NLRP3 inflammasome priming and activation in murine colonic macrophages, including *Nlrp3* (Figures 4B and S4D), suggesting that IgG-associated IL-1β production by intestinal macrophages might be dependent on the NLRP3 inflammasome. Inflammasome assembly is a two-step process; ‘‘signal 1’’ is required for NLRP3 transcription, and ‘‘signal 2’’, including stimuli such as adenosine trisphosphate (ATP) and ROS, for the generation of a multimeric-complex-containing NLRP3, ASC, and pro-caspase-1 (Schroder and Tschopp, 2010). Priming of murine bone-marrow-derived macrophages (BMDMs) with IgG ICs, followed by ATP, resulted

(B and C) Correlation summary of *FCGR2A* expression with UC-associated cytokine and chemokine gene transcripts (B) and correlation with *IL1B* and *CXCL8* (C) in n = 85 mucosal biopsies. Data are from GEO: GSE59071.

(D) Hierarchical clustering of cytokine and chemokine and *FCGR2A* expression in healthy controls and non-inflamed and inflamed UC patients. Data are derived from GEO: GSE38713.

(E and F) Correlation of *Fcgr3* expression with *Il1b* (E) and candidate-gene (F) expression in whole inflamed colonic tissue by qPCR (n = 16). Data are representative of two independent experiments.

(G) *IL1B* and *CXCL8* expression in healthy human LPMC stimulated with Ova or Ova-IC for 16 h (n = 5 per condition). Means ± SEM are indicated. Data are representative of two independent experiments.

(H) IL-1β, CXCL1, and CXCL2 in supernatants of inflamed murine LPMCs stimulated with Ova or Ova-IC for 16 h (n = 3-9). Paired samples represent LPMCs from a single mouse. Data are pooled from two independent experiments.

(I) Flow-cytometry profiling of pro-IL-1β-expressing cells at day 14 after aDSS (n = 6). Medians are indicated. Data are representative of three independent experiments.

(J) Pro-IL-1β expression by colonic $CX3CR1^{+}$ MNP subsets and neutrophils in *Rag2*$^{-/-}$ mice treated with control or anti-flagellin-enriched serum IgG and 7-day aDSS or uninflamed H_2O-treated controls (n = 5 per group). Medians are indicated.

(K) Day 7 weight loss for aDSS-treated mice shown in (J) (n = 5 per group). Medians are indicated.

p values were calculated via limma with multiple correction using the BH procedure (A), linear regression analysis (B, C, E, and F), Student’s two-tailed t test (G), ratio paired t test (H and I), or the Kruskal-Wallis test with Dunn’s multiple-comparisons test (J). *p < 0.05, **p < 0.01, ***p < 0.001, ****p < 0.0001. See also Figure S3.

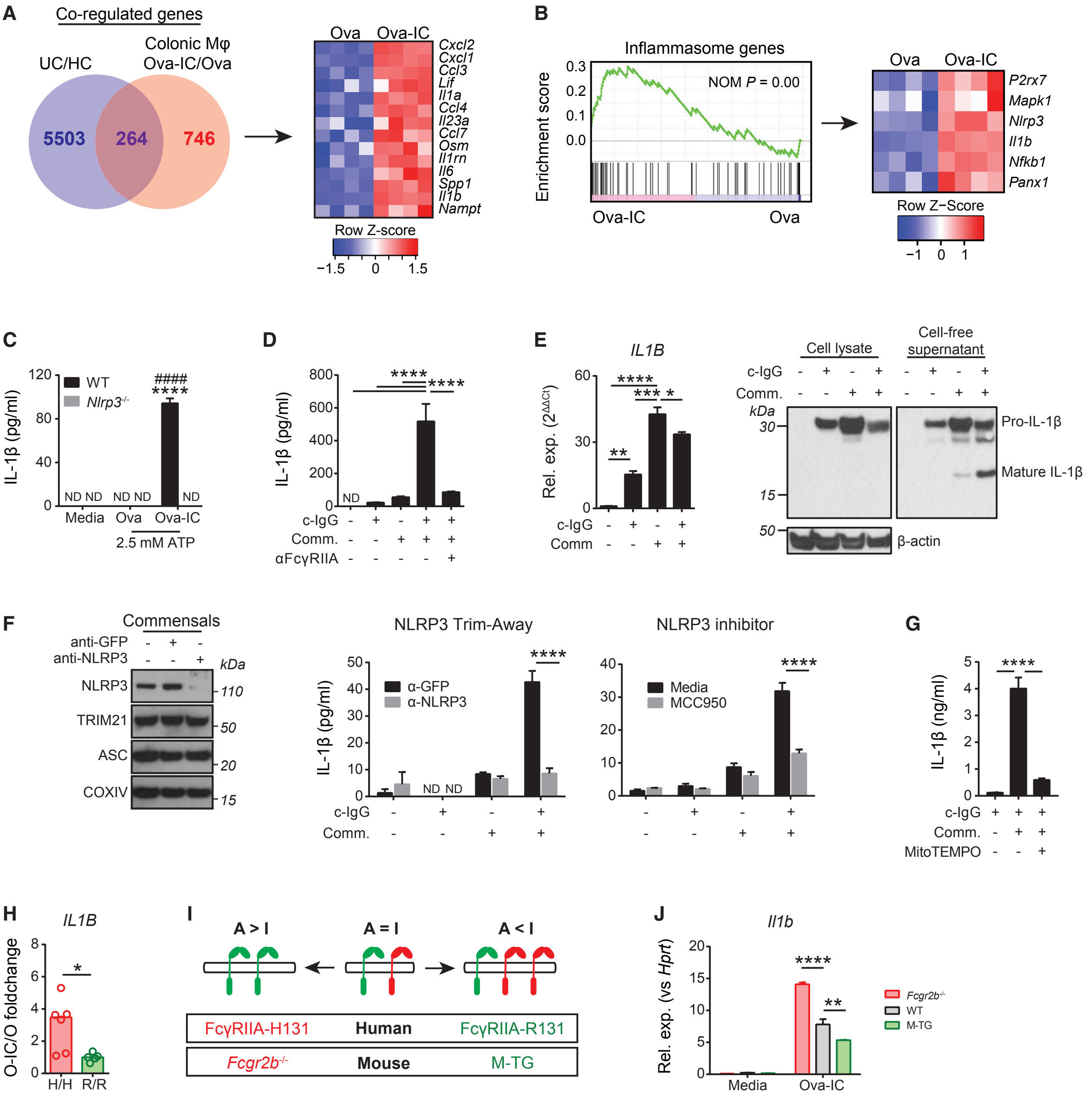

Figure 4. IgG-Induced IL-1β Production by Human Macrophages

(A) Venn diagram of significant co-expressed genes between UC and Ova-IC-stimulated colonic macrophages (left) and IC-induced UC-associated cytokines and chemokines (right), as determined by sdef. Human UC data are from GEO: GSE38713 (UC = 15, HC = 13). For macrophage stimulation, n = 4 per condition.

(B) GSEA of inflammasome genes (left) and heatmap of selected core enrichment genes (right) in flow-sorted murine intestinal CX3CR1^{+}Ly6C^{lo}MHC-II^{hi} macrophages stimulated with Ova and Ova-IC for 4 h (n = 4 per condition).

(C) IL-1β production by WT and *Nlrp3*$^{-/-}$ BMDMs primed with Ova and Ova-IC for 4 h followed by 30 min of ATP stimulation (****, WT versus *Nlrp3*$^{-/-}$ Ova-IC; ####, WT Ova versus Ova-IC) (n = 3 per condition). Means ± SEM are indicated. Data are representative of three independent experiments. ND, not detected.

(D) IL-1β ELISA of human MDMs stimulated with plate-coated IgG (c-IgG) and intestinal commensals (Comm.) for 24 h ± anti-FcγRIIA IgG-blocking antibody (n = 3 per condition). Means ± SEM are indicated. Data are representative of two independent experiments.

(E) qPCR of *IL1B* mRNA (left, n = 3 per condition) and western blot of IL-1β in cell lysates and cell-free supernatants (right) from human MDMs as stimulated in (D). For qPCR (left), means ± SEM are indicated. Data are representative of two independent experiments.

(F) The effect of NLRP3 inhibition on IgG-induced MDM IL-1β production. Western blot (left) and IL-1β production (left, ELISA) by human MDMs stimulated as in (D) after NLRP3 "Trim-Away" or ± NLRP3 inhibitor MCC950 (right, ELISA) (n = 3 per condition). Means ± SEM are indicated.

(legend continued on next page)

in IL-1β secretion in wild-type (WT) but not *Nlrp3*-deficient macrophages (Figure 4C). This induction was greater than either model antigen (Ova) or anti-Ova immune serum stimulation alone (Figure S4E). IgG IC stimulation of human monocyte-derived macrophages (MDMs) in the presence of a fecal commensal suspension or lipopolysaccharide (LPS) was sufficient to induce mature IL-1β secretion, an effect almost entirely abrogated by blockade of the UC-associated receptor FcγRIIA (Figures 4D and S4F). Both IgG-IC and fecal commensals were effective in inducing *IL1B* transcript and pro-IL-1β protein expression (Figure 4E). IgG IC alone was insufficient to generate cleaved IL-1β, and although stimulation with fecal commensals resulted in the production of some mature IL-1β, only the combined stimulation of IgG ICs and fecal commensals was effective in driving substantial cleavage of pro-IL-1β to mature IL-1β (Figure 4E). This demonstrates that FcγR cross-linking provides a robust priming "signal 1" for NLRP3 inflammasome assembly. To test the extent to which IL-1β production was dependent on NLRP3 in human macrophages, we performed NLRP3 protein knockdown via the "Trim-Away" technique (Figure 4F) (Clift et al., 2017). This substantially abrogated IgG-induced macrophage IL-1β production, as did the addition of an NLRP3 inhibitor MCC950 (Figure 4F). IL-1β production by human MDMs after combined stimulation of IgG ICs and commensals or LPS was also reduced by the addition of MitoTEMPO, a mitochondrial ROS inhibitor (Figures 4G and S4G). Together, these data suggest that in UC, IgG-commensal ICs cross-link intestinal macrophage FcγRIIA and thus result in NLRP3-dependent IL-1β production.

To link these observations to the genetic variants associated with UC, we investigated the impact of the FcγRIIA-H/R131 SNP on macrophage IL-1β production. After stimulation with IgG ICs, we observed higher *IL1B* expression in MDMs obtained from subjects with the FcγRIIA-H/H131 genotype than in FcγRIIA-R/R131 macrophages (Figure 4H), demonstrating that this SNP, and the functional effect it confers on the FcγR A:I ratio, determines the magnitude of IL-1β induction in this context. Modeling FcγRIIA function *in vivo* is challenging because mice do not express FcγRIIA, and mice deficient in the orthologous receptor *Fcgr3* have a complete loss of activating signaling from this receptor, which does not reflect the graded functional effect of the FcγRIIA-H/R131 SNP. Our analysis of intestinal macrophages demonstrated that FcγR expression is dominated by the single inhibitory FcγRIIB in both mice and humans and one functionally homologous low-affinity activating FcγR (FcγRIII in mice and FcγRIIA in humans) (Figures 2G and 2I). Therefore, we used mice with intact activating FcγR signaling but a variable FcγR A:I ratio due to absent, WT, or high-inhibitory-receptor expression, allowing interrogation of the effect of graded activating FcγR signaling strength on intestinal inflammation (Figure 4I). *In vitro*, IgG IC stimulation of BMDM from mice with a low FcγR A:I ratio due to macrophage-specific over-expression of FcγRIIB (Brownlie et al., 2008) (macrophage-transgenic [M-TG]) resulted in lower *Il1b* induction than in BMDM from WT mice, in contrast to BMDM from mice with a high A:I ratio due to FcγRIIB deficiency, in which *Il1b* induction was significantly higher than in WT mice (Figure 4J), in a manner analogous to the FcγRIIA-H/R131 variant (Figures 4H and 4I). Similar results were observed for *Cxcl1* and *Cxcl2* (Figure S4H). We concluded, therefore, that these mice represent a useful model for studying the effects of differing FcγR A:I ratios *in vivo*.

MNP FcγR A:I Ratio Modulates Intestinal Inflammation

Compared with co-housed WT controls, *Fcgr2b*$^{-/-}$ mice subjected to aDSS had a more severe disease course, including impaired weight recovery from day 7 onward (Figure 5A), a time point at which there is significant induction of anti-commensal IgG (Figure 1E). Similarly, in an independent experiment, *Fcgr2b*$^{-/-}$ mice had more severe disease than co-housed, littermate WT controls, with *Fcgr2b*$^{+/-}$ demonstrating an intermediate phenotype (Figure S5A). Compared with mice reconstituted with WT bone marrow, WT mice reconstituted with *Fcgr2b*$^{-/-}$ bone marrow also showed increased susceptibly to more severe disease after exposure to aDSS (Figure S5B). After aDSS treatment, *Fcgr2b*$^{-/-}$ mice had increased colon and spleen weights and lymph node and spleen enlargement (Figure S5C), as well as marked infiltration of CD45^{+} cells into the colonic mucosa and submucosa in comparison with those of co-housed WT controls (Figure 5B). This infiltration included neutrophils (Figure 5C), indicative of on-going inflammation 3 weeks after exposure to aDSS. Inflamed *Fcgr2b*$^{-/-}$ colons had higher levels of *Il1b* transcripts (Figure S5D) and increased numbers of pro-IL-1β-expressing cells than WT controls (Figure S5E). We observed significantly higher proportions of pro-IL-1β^{+} colonic CX3CR1^{+} MNPs in *Fcgr2b*$^{-/-}$ mice than in WT counterparts, particularly in newly recruited Ly6C^{hi} monocytes and Ly6C^{+}MHC-II^{+} macrophages (Figures 5D, 5E, and S5F), as well as minimal pro-IL-1β expression in colonic neutrophils (Figure S5G). Furthermore, flow-sorted colonic MHC-II^{+} MNPs isolated from *Fcgrb*$^{-/-}$ mice with aDSS had increased *Cxcl1* and *Cxcl2* transcripts compared with those obtained from co-housed WT mice (Figure 5F), consistent with our observations *in vitro*.

Next, we investigated intestinal pathology in FcγRIIB-M-TG mice and confirmed increased FcγRIIB expression on colonic MNPs and that there was no difference in activating FcγRs (Figure S5H) or in FcγRIIB expression on colonic neutrophils, DCs, B cells, or epithelial cells in FcγRIIB-M-TG mice compared with non-transgenic (N-TG) controls (Figure S5I). After exposure

(G) IL-1β production by human MDMs stimulated as in (D) ± mitochondrial ROS inhibitor mitoTEMPO (n = 3 per condition). Means ± SEM are indicated. Data are representative of 2 independent experiments.

(H) Fold change in *IL1B* expression in Ova-IC-stimulated MDMs, normalized to R/R fold change (n = 6 per group). Data are pooled from 3 independent experiments. Medians are indicated.

(I) Schematic of FcγR A:I ratios with *FCGR2A* SNP (dbSNP: rs1801274) and murine transgenic models.

(J) *Il1b* induction in WT, *Fcgr2b*$^{-/-}$, and M-TG BMDMs stimulated with Ova and Ova-IC for 3 h (n = 3 per condition). Means ± SEM are indicated. Data are representative of 3 independent experiments.

p values were calculated via the standard DESeq2 method with multiple correction using the BH procedure (A and B), two-way ANOVA with Tukey's multiple comparisons test (C, F, and J), one-way ANOVA with Tukey's multiple-comparisons test (D, E, and G), or parametric Student's t test (H). *$p < 0.05$, **$p < 0.01$, ***$p < 0.001$, ****$p < 0.0001$. See also Figure S4.

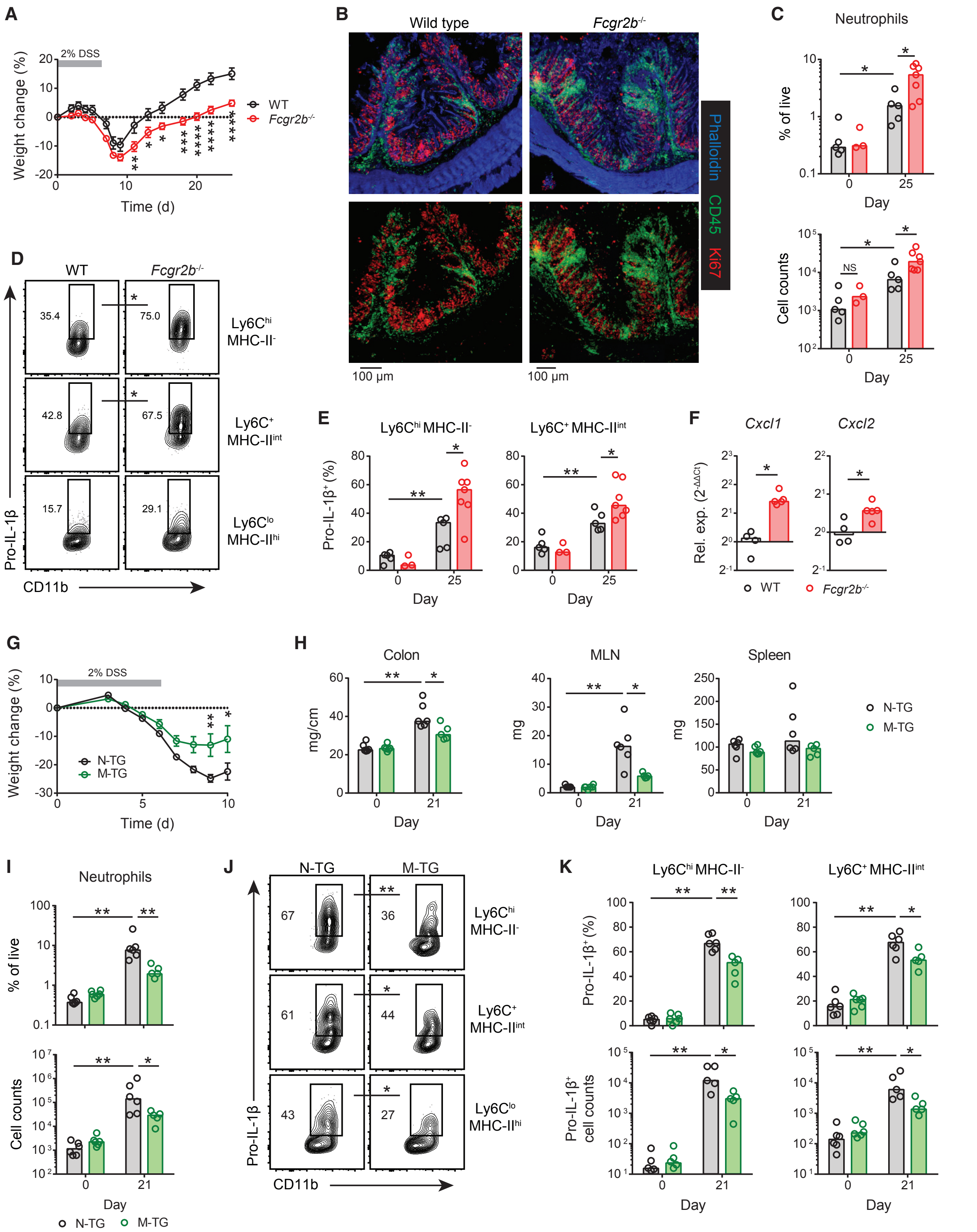

(legend on next page)

to aDSS, in contrast to *Fcgr2b*$^{-/-}$ mice, FcγRIIB-M-TG mice had a less severe clinical disease course than did N-TG controls, as evidenced by lower weight loss (Figure 5G), lower colonic and MLN weight (Figure 5H), and a trend toward increased colon length (Figure S5J), but no difference in spleen size (Figure 5H). Colonic neutrophil infiltration was also significantly reduced in M-TG mice, consistent with improved disease resolution (Figure 5I). There was a trend toward reduced global *Il1b*, *Cxcl1*, *Cxcl2*, and *Ccl2* transcripts within the colons of these mice (Figure S5K), whereas pro-IL-1β protein was significantly reduced in intestinal CX3CR1^{+} MNPs in FcγRIIB-M-TG mice (Figures 5J, 5K, and S5L).

Given that FcγRIIB modulates the B cell activation threshold, we profiled B cell class switching and commensal-reactive IgG responses in our mouse models. We observed an increase in frequency and count of class-switched B cells in the MLN and spleen of *Fcgr2b*$^{-/-}$ mice but no difference in total anti-commensal IgG titer after aDSS (Figures S5M and S5O). We observed no significant difference in class-switched B cells between the MLN of FcγRIIB-M-TG animals at day 21 after aDSS and the MLN of N-TG mice and found similar levels of anti-commensal IgG titers between these two strains (Figures S5N and S5O). Together, these data demonstrate that the differences observed in macrophage IL-1β and disease severity in mice with differing FcγR A:I ratio are not related to differences in the titers of anti-commensal IgG.

MNP FcγR A:I Ratio Modulates Intestinal Type 17 Immunity

GWASs have implicated the IL-23-IL-17A cytokine axis in both CD and UC (Cătană et al., 2015). IL-17A is expressed in healthy mucosa and contributes to intestinal homeostasis, but in excess might promote inflammation (Leppkes et al., 2009). Th17 cells are key producers of IL-17A within the intestine, and inhibition of these cells can reduce intestinal inflammation (Withers et al., 2016). As well as T-cell-independent effects, a key biological effect of IL-1β is to induce the differentiation and maintenance of Th17 cells (Chung et al., 2009; Shaw et al., 2012). Given that our data demonstrated that IgG ICs potently induce IL-1β production in intestinal macrophages in mouse and human and that IgG-IC-stimulated colonic macrophages exhibit a Th17-polarizing phenotype (Figure S6A), we sought to determine whether macrophage FcγR A:I ratio was sufficient to affect intestinal type 17 immunity. Analysis of UC biopsies identified a significant positive correlation between *IL17A* and *FCGR2A* (Figure S6B). Furthermore, IgG IC stimulation of inflamed colonic LPMCs resulted in the production of type-17-associated cytokines, including IL-17, granulocyte-macrophage colony-stimulating factor (GM-CSF), and IL-22 (Figure 6A). *In vivo*, the magnitude of intestinal type 17 responses was determined by the FcγR A:I ratio; in *Fcgr2b*$^{-/-}$ mice, we observed an increase in colonic *Il17a*, *Csf2*, and *Il22* transcripts after aDSS (Figure 6B), whereas there was little change in global *Ifng* levels (Figure S6C). Intracellular cytokine staining of colonic CD3ε^{+} T cell subsets (Figure S6D) demonstrated a significant increase in the frequency (Figure 6C) and absolute cell count (Figure 6D) of both IL-17A^{+} γδ and αβ CD4^{+} T cell subsets in the recovery phase of aDSS colitis. We also observed a significant increase in IL-22 production by these T cell subsets (Figures S6E and S6F), although the magnitude of this response was less than for IL-17A. Globally, there was a significant correlation between *Il1b* and type 17 cytokine gene transcript levels in inflamed colons (Figure S6G), suggesting that FcγR-induced type 17 responses might be IL-1β dependent.

In support of this, augmented IL-17A production by T cells in *Fcgr2b*$^{-/-}$ mice was completely abrogated by treatment with an anti-IL-1R1-blocking antibody (Figures 6E and S6H), as was the increase in IL-22 (Figure S6I). Conversely, there were specifically fewer mucosal IL-17A-producing CD4^{+} αβ T cells and γδ T cells after aDSS in FcγRIIB-M-TG mice than in N-TG controls (Figures 6F and 6G). Little change in colonic B cells was observed after IL-1β blockade in *Fcgr2b*$^{-/-}$ mice (Figure S6J); however, there was a reduction in the severity of colitis (Figure 6H) and colonic neutrophil infiltration (Figure 6I), directly implicating this pathway in detrimental immune responses driven by dysregulated FcγR signaling.

Figure 5. MNP FcγR A:I Ratio Modulates Intestinal Inflammation

(A) Weight loss in co-housed sex-matched WT (black circles, n = 8) and *Fcgr2b*$^{-/-}$ (red circles, n = 9) mice after a single 6-day course of 2% DSS. Means ± SEM are indicated. Data are representative of 3 independent experiments.

(B) Confocal microscopy of colonic CD45^{+} leukocytes in mice treated as in (A). Data are representative of 2 independent experiments.

(C) Flow-cytometric quantification of colonic neutrophil frequency (top) and absolute neutrophil counts (bottom) in WT and *Fcgr2b*$^{-/-}$ colons at day 25 after aDSS (n = 5–7 per group) or in healthy controls (day 0) (n = 3–5 per group). Medians are indicated. Data are representative of 3 independent experiments.

(D) Pro-IL-1β expression by colonic CD11b^{+}CX3CR1^{+} MNPs at day 25 after aDSS (n = 5–7 per group).

(E) Frequency of pro-IL-1β expression within the Ly6C^{hi}MHC-II^{-} monocyte and Ly6C^{+}MHC-II^{int} macrophage populations as shown in aDSS (n = 5–7 per group) versus controls (day 0) (n = 3–5 per group). Medians are indicated. Data are representative of 3 independent experiments.

(F) Chemokine mRNA levels in flow-sorted Ly6C^{lo}MHC-II^{hi} colonic macrophages at day 21 after aDSS (n = 4–5 per group). Data normalized to WT mRNA levels. Medians are indicated.

(G and H) Weight loss (G) and clinical colonic and lymphoid organ features (H) of co-housed sex-matched M-TG (green circles, n = 5 or 6) and N-TG littermate controls (black circles, n = 6) after aDSS or in healthy controls (day 0 in H). Means ± SEM (weight loss) and medians (colon, MLN, and spleen weight) are indicated. Data are representative of 3 independent experiments.

(I) Flow-cytometric quantification of colonic neutrophil frequency (top) and absolute neutrophil count (bottom) in N-TG and M-TG mice at day 21 after aDSS (n = 6 per group) versus controls (day 0) (n = 5–6 per group). Medians are indicated.

(J) Pro-IL-1β expression by colonic CX3CR1^{+} MNP subsets in N-TG and M-TG mice treated as in (I) (n = 5 or 6 per group).

(K) Frequency (top) and absolute cell count (bottom) of pro-IL-1β-expressing MNP subsets in N-TG and M-TG mice treated as in (I). Medians are indicated. Data are representative of two independent experiments.

p values calculated using a two-way ANOVA with Bonferroni's multiple comparisons test (A and G), or the nonparametric Mann-Whitney U test (C-F, H-K). *$p < 0.05$, **$p < 0.01$, ***$p < 0.001$, ****$p < 0.0001$. See also Figure S5.

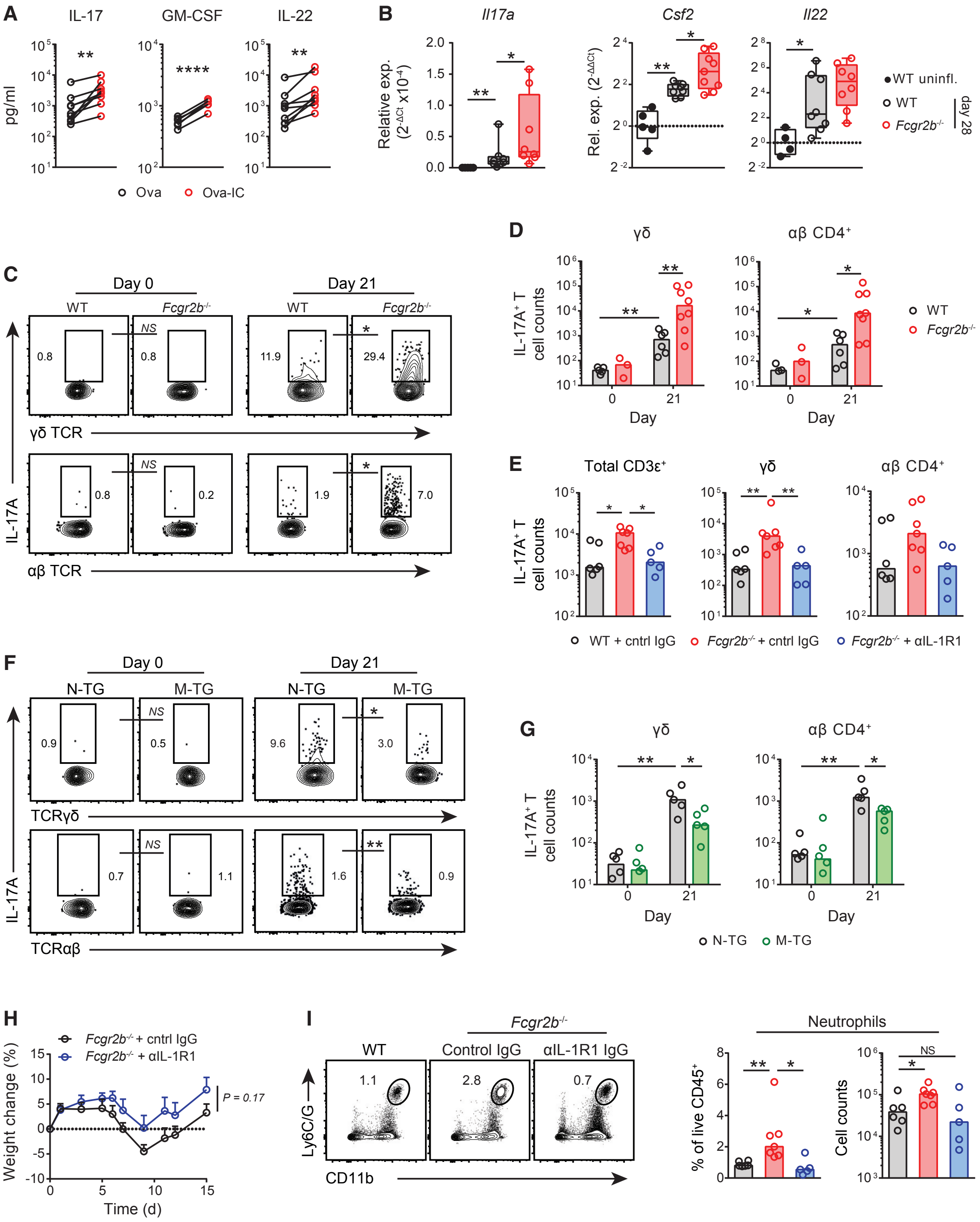

Figure 6. MNP FcγR A:I Ratio Modulates Intestinal Type 17 Immunity

(A) Type-17-associated cytokine production by day-14 LPMCs stimulated with Ova and Ova-IC for 16 h (n = 5–9). Paired samples represent cells isolated from the same mouse. Data are pooled from 2 independent experiments.

(B) qPCR of type 17 cytokines in WT and *Fcgr2b*$^{-/-}$ whole colonic tissue after cDSS versus controls (n = 4–9 per group). Data are normalized to uninflamed healthy colon. Medians are indicated. Data are representative of 2 independent experiments.

(C) Flow-cytometry plots of colonic IL-17A-expressing T cell subsets in co-housed sex-matched WT and *Fcgr2b*$^{-/-}$ mice at day 21 after aDSS (n = 6–8 per group) versus controls (day 0) (n = 3–5 per group).

(D) Quantification of absolute numbers of colonic IL-17A-producing T cells shown in (C). Medians are indicated. Data are representative of 3 independent experiments.

(legend continued on next page)

DISCUSSION

IgG-positive cells were first described in colonic biopsies from IBD patients more than 40 years ago by Baklien and Brandtzaeg (1975). Despite subsequent confirmation of their commensal specificity (Macpherson et al., 1996), there has been little widespread acceptance that commensal-IgG immune complexes play a pathogenic role in UC. The subsequent identification of *FCGR2A*A519G* (dnSNP: rs1801274) as the most significant non-HLA genetic variant associated with UC in a Japanese GWAS (Asano et al., 2009) further supports this concept. This non-synonymous SNP results in an amino acid substitution that alters IgG binding affinity, the low-affinity variant (FcγRIIA-R131) is protective in both candidate gene studies (odds ratio 0.70–0.84) (Weersma et al., 2010; Yang et al., 2011) and a meta-analysis of IBD GWASs (Jostins et al., 2012). Our study sheds light on the mechanisms underpinning these observations, demonstrating an increase in anti-commensal IgG and in the FcγR A:I ratio of mucosal immune cells in UC. The net functional effect of this is to lower the cellular activation threshold, rendering intestinal macrophages more readily activated by local IgG ICs—an effect significantly offset by the low-affinity FcγRIIA-R131.

The generic effects of cross-linking FcγR on monocyte and macrophage cytokine production have been widely studied over many years by ourselves and others (Clatworthy and Smith, 2004; Clynes et al., 1999), including in intestinal macrophages (Uo et al., 2013), demonstrating increases in TNFα, IL-6, IL-1β, IL-10, and TL1A, to name but a few. Of these many potentially pathogenic cytokines, our study specifically identifies IL-1β as a key driver of IgG-associated inflammation in UC and reveals the molecular mechanisms underpinning this.

IL-1β correlates with disease severity in patients with IBD (Ligumsky et al., 1990; Reinecker et al., 1993), but its role in intestinal immunity is complex. Mice deficient in IL-1β exhibit a severe non-healing disease after DSS colitis, suggesting involvement in intestinal repair (Bersudsky et al., 2014). In contrast, IL-1β can promote chronic intestinal inflammation by inducing IL-17A-secreting innate lymphoid cells and Th17 cells (Coccia et al., 2012). Our data identify IL-1β as a key mediator of IgG- and FcγR-associated intestinal inflammation and show that it might act to induce type 17 immunity via effects on Th17 cells, γδ T cells and, potentially, group 3 innate lymphoid cells (although we did not specifically examine the latter here). We also found that IL-1β could have Th17-cell-independent effects, consistent with previous reports, for example, by promoting neutrophil and monocyte recruitment (Dinarello, 2009). Notably, we observed IL-1β-dependent IL-17A and IL-22 production in both γδ and $CD4^+$ αβ T cells, implicating both innate and adaptive T cell subsets in FcγR-driven mucosal inflammation. It will be of interest in future studies to determine the contribution of IgG-induced FcγR signaling to type 17 immunity in other models of intestinal inflammation, particularly those with a dominant role for adaptive T cells, for example, after T cell transfer into $Rag^{-/-}$ mice. A careful description of Th17 cytokines in this model will be of clinical relevance, given reports that isolated IL-17A blockade can exacerbate intestinal inflammation (Hueber et al., 2012).

Although anti-commensal IgG, particularly anti-flagellin antibodies are present in CD, the association of FcγR variants with disease susceptibility is less robust than in UC (Jostins et al., 2012). Differential effects of genetic susceptibility loci in CD and UC are well recognized. For example, genetic variants that promote intracellular pathogen recognition and clearance are associated with CD and not with UC (Abraham and Cho, 2006). In this context, it is notable that the FcγRIIA-R131 polymorphism increases susceptibility to a number of infections (Clatworthy, 2014) and that IgG and FcγRs promote defense against mycobacterial infection (Lu et al., 2016). Indeed, in mice, IgG and activating FcγRs are protective against the systemic spread of *Citrobacter rodentium*, an enteropathic bacteria (Masuda et al., 2008). Therefore, the deleterious effects of FcγRIIA-R131 on bacterial clearance might outweigh its beneficial, anti-inflammatory effects in CD.

Our data also have therapeutic implications, identifying IgG and activating FcγR signaling as potential therapeutic targets in UC. The only randomized controlled trial to investigate the use of B cell depletion in (treatment-resistant) UC has shown no benefit but is substantially under-powered with only n = 24 subjects (Leiper et al., 2011). Given that randomized trials of drugs licensed for IBD treatment typically require the inclusion of hundreds of patients to demonstrate efficacy (Feagan et al., 2013; Sandborn et al., 2012), the question of whether B cell manipulation might be of benefit in UC remains to be addressed. Agents to target activating FcγR signaling might be of utility and include Syk (spleen tyrosine kinase) inhibitors, which are currently being assessed in rheumatoid arthritis (Weinblatt et al., 2010) and glomerulonephritis (Ma et al., 2017). Alternatively, application of an FcγRIIB agonist (Bosques and Manning, 2016) in UC would potentially decrease the FcγR A:I ratio, an effect functionally analogous to the presence of the low affinity FcγRIIA-R131. Our data suggest that targeting FcγR signaling in UC would have a potential advantage over single-cytokine blockade because of its impact on the production of multiple pro-inflammatory cytokines and chemokines. Finally, it is notable that a trial of IL-1R1 blockade using anakinra in acute severe UC (ISRCTN43717130) is underway in the UK (Thomas et al., 2019).

(E) Quantification of absolute numbers of colonic IL-17A-producing T cell subsets in co-housed WT and *Fcgr2b*$^{-/-}$ mice at day 15 after aDSS and weekly treatment with anti-IL-1R1 IgG-blocking antibody or control IgG (n = 5–7 per group). Medians are indicated. Data are representative of two independent experiments.

(F) Colonic IL-17A-expressing T cell subsets in M-TG and N-TG littermate controls at day 21 after aDSS (n = 5 or 6 per group) versus controls (n = 5 or 6 per group). Data are representative of two independent experiments.

(G) Quantification of absolute cell counts of colonic IL-17A-producing T cell subsets as shown in (F). Medians are indicated.

(H) Weight loss of *Fcgr2b*$^{-/-}$ mice treated with control or anti-IL-1R1 IgG antibodies after aDSS treatment (n = 5–7 per group). Mean ± SEM are indicated. Data are representative of two independent experiments.

(I) Colonic neutrophil infiltration in WT and *Fcgr2b*$^{-/-}$ mice treated as in (H) (n = 5–7 per group). Medians are indicated.

p values were calculated with a ratio paired t test (A), the nonparametric Mann-Whitney U test (B–G and I), or a two-way ANOVA (H). *$p < 0.05$, **$p < 0.01$, ***$p < 0.001$, ****$p < 0.0001$. See also Figure S6.

In summary, our study sheds light on the observed genetic association of FcγRIIA polymorphisms with UC, revealing the specific molecular mechanisms by which anti-commensal IgG augment inflammation and identifying novel therapeutic targets.

STAR★METHODS

Detailed methods are provided in the online version of this paper and include the following:

- KEY RESOURCES TABLE
- CONTACT FOR REAGENT AND RESOURCE SHARING
- EXPERIMENTAL MODEL AND SUBJECT DETAILS
  - Mouse strains
  - Human samples
  - Commensal strains
- METHOD DETAILS
  - DSS-induced experimental colitis
  - Flagellin immunization
  - Murine primary cell isolation
  - Human primary cell isolation
  - Flow cytometry
  - Flow sorting
  - Microbial flow cytometry
  - Serum anti-commensal IgG ELISA
  - Luminal IgG ELISA
  - Macrophage and intestinal immune cell culture
  - IgG stimulation
  - Trim-Away
  - Immunoblotting
  - *FCGR2A* genotyping
  - RNA extraction and reverse transcription
  - Quantitative polymerase chain reaction
  - Cytokine/chemokine ELISA
  - Immunofluorescence
  - RNA-seq sample preparation
  - RNA sequencing and analysis
  - Microarray
  - Hierarchical clustering
  - Single cell RNA-seq
- QUANTIFICATION AND STATISTICAL ANALYSIS
- DATA AND SOFTWARE AVAILABILITY

SUPPLEMENTAL INFORMATION

Supplemental Information can be found with the article online at https://doi.org/10.1016/j.immuni.2019.02.006.

ACKNOWLEDGMENTS

The authors thank Arthur Kaser, Ken Smith, and Leo James for helpful discussions; the NIHR Cambridge BCR Cell Phenotyping Hub; and all donors and their families. The Molecular Immunity Unit is within the MRC Laboratory of Molecular Biology, and the authors are grateful for the use of the core facilities. T.C.D. was supported by a Wellcome Trust Infection, Immunity, and Inflammation Programme PhD fellowship. B.J.S. was supported by a Wellcome Trust PhD Fellowship (216366/Z/19/Z). C.J. was funded by the China Scholarship Council (201408060241). D.C. is supported by the Medical Research Council (UK; U105181010). L.I.L. is supported by an EMBO Long-Term Fellowship (1487-2015) and an Australian National Health Medical Research Council CJ Martin Fellowship (GNT1124612). J.R.F., R.J.M., and M.R.C. were supported by the National Institute of Health Research (NIHR), the Cambridge Biomedical Research Centre, and the NIHR Blood and Transplant Research Unit (NIHR BTRU-2014-10027). M.R.C. and T.W.D. were supported by a Medical Research Council New Investigator Research Grant (MR/N024907/1), and M.R.C. is also supported by an NIHR Research Professorship (RP-2017-08-ST2-002).

AUTHOR CONTRIBUTIONS

T.C.D. designed and performed experiments, analyzed data, and co-wrote the manuscript. T.W.D. and J.R.F performed experiments and analyzed data. R.J.M, D.C, A.F., B.J.S., C.J., L.I.L, S.C., and E.J.M.M. performed experiments. K.S., K.S.-P., and M.P. provided human tissue samples. M.R.C. conceived the study, designed experiments, analyzed data, and co-wrote the manuscript.

DECLARATION OF INTERESTS

The authors declare no competing interests.

Received: April 27, 2018
Revised: December 17, 2018
Accepted: February 11, 2019
Published: March 12, 2019

REFERENCES

Abraham, C., and Cho, J.H. (2006). Functional consequences of NOD2 (CARD15) mutations. Inflamm. Bowel Dis. *12*, 641–650.

Aibar, S., González-Blas, C.B., Moerman, T., Huynh-Thu, V.A., Imrichova, H., Hulselmans, G., Rambow, F., Marine, J.C., Geurts, P., Aerts, J., et al. (2017). SCENIC: single-cell regulatory network inference and clustering. Nat. Methods *14*, 1083–1086.

Arijs, I., De Hertogh, G., Lemaire, K., Quintens, R., Van Lommel, L., Van Steen, K., Leemans, P., Cleynen, I., Van Assche, G., Vermeire, S., et al. (2009a). Mucosal gene expression of antimicrobial peptides in inflammatory bowel disease before and after first infliximab treatment. PLoS ONE *4*, e7984.

Arijs, I., Li, K., Toedter, G., Quintens, R., Van Lommel, L., Van Steen, K., Leemans, P., De Hertogh, G., Lemaire, K., Ferrante, M., et al. (2009b). Mucosal gene signatures to predict response to infliximab in patients with ulcerative colitis. Gut *58*, 1612–1619.

Asano, K., Matsushita, T., Umeno, J., Hosono, N., Takahashi, A., Kawaguchi, T., Matsumoto, T., Matsui, T., Kakuta, Y., Kinouchi, Y., et al. (2009). A genome-wide association study identifies three new susceptibility loci for ulcerative colitis in the Japanese population. Nat. Genet. *41*, 1325–1329.

Bain, C.C., Bravo-Blas, A., Scott, C.L., Perdiguero, E.G., Geissmann, F., Henri, S., Malissen, B., Osborne, L.C., Artis, D., and Mowat, A.M. (2014). Constant replenishment from circulating monocytes maintains the macrophage pool in the intestine of adult mice. Nat. Immunol. *15*, 929–937.

Baklien, K., and Brandtzaeg, P. (1975). Comparative mapping of the local distribution of immunoglobulin-containing cells in ulcerative colitis and Crohn's disease of the colon. Clin. Exp. Immunol. *22*, 197–209.

Bersudsky, M., Luski, L., Fishman, D., White, R.M., Ziv-Sokolovskaya, N., Dotan, S., Rider, P., Kaplanov, I., Aychek, T., Dinarello, C.A., et al. (2014). Non-redundant properties of IL-1α and IL-1β during acute colon inflammation in mice. Gut *63*, 598–609.

Bolland, S., and Ravetch, J.V. (2000). Spontaneous autoimmune disease in Fc(gamma)RIIB-deficient mice results from strain-specific epistasis. Immunity *13*, 277–285.

Bosques, C.J., and Manning, A.M. (2016). Fc-gamma receptors: Attractive targets for autoimmune drug discovery searching for intelligent therapeutic designs. Autoimmun. Rev. *15*, 1081–1088.

Breynaert, C., Dresselaers, T., Perrier, C., Arijs, I., Cremer, J., Van Lommel, L., Van Steen, K., Ferrante, M., Schuit, F., Vermeire, S., et al. (2013). Unique gene expression and MR T2 relaxometry patterns define chronic murine dextran

sodium sulphate colitis as a model for connective tissue changes in human Crohn's disease. PLoS ONE *8*, e68876.

Brownlie, R.J., Lawlor, K.E., Niederer, H.A., Cutler, A.J., Xiang, Z., Clatworthy, M.R., Floto, R.A., Greaves, D.R., Lyons, P.A., and Smith, K.G. (2008). Distinct cell-specific control of autoimmunity and infection by FcgammaRIIb. J. Exp. Med. *205*, 883–895.

Cătană, C.S., Berindan Neagoe, I., Cozma, V., Magdaş, C., Tăbăran, F., and Dumitraşcu, D.L. (2015). Contribution of the IL-17/IL-23 axis to the pathogenesis of inflammatory bowel disease. World J. Gastroenterol. *21*, 5823–5830.

Chung, Y., Chang, S.H., Martinez, G.J., Yang, X.O., Nurieva, R., Kang, H.S., Ma, L., Watowich, S.S., Jetten, A.M., Tian, Q., and Dong, C. (2009). Critical regulation of early Th17 cell differentiation by interleukin-1 signaling. Immunity *30*, 576–587.

Clatworthy, M.R. (2014). Fcγ Receptor polymorphisms and susceptibility to infection. In Antibody Fc: Linking Adaptive and Innate Immunity,, M. Ackerman and F. Nimmerjahn, eds. (Academic Press), pp. 217–238.

Clatworthy, M.R., and Smith, K.G. (2004). FcgammaRIIb balances efficient pathogen clearance and the cytokine-mediated consequences of sepsis. J. Exp. Med. *199*, 717–723.

Clift, D., McEwan, W.A., Labzin, L.I., Konieczny, V., Mogessie, B., James, L.C., and Schuh, M. (2017). A method for the acute and rapid degradation of endogenous proteins. Cell *171*, 1692–1706.e18.

Clynes, R., Maizes, J.S., Guinamard, R., Ono, M., Takai, T., and Ravetch, J.V. (1999). Modulation of immune complex-induced inflammation in vivo by the coordinate expression of activation and inhibitory Fc receptors. J. Exp. Med. *189*, 179–185.

Coccia, M., Harrison, O.J., Schiering, C., Asquith, M.J., Becher, B., Powrie, F., and Maloy, K.J. (2012). IL-1β mediates chronic intestinal inflammation by promoting the accumulation of IL-17A secreting innate lymphoid cells and CD4(+) Th17 cells. J. Exp. Med. *209*, 1595–1609.

Dinarello, C.A. (2009). Immunological and inflammatory functions of the interleukin-1 family. Annu. Rev. Immunol. *27*, 519–550.

Fagarasan, S. (2008). Evolution, development, mechanism and function of IgA in the gut. Curr. Opin. Immunol. *20*, 170–177.

Feagan, B.G., Rutgeerts, P., Sands, B.E., Hanauer, S., Colombel, J.F., Sandborn, W.J., Van Assche, G., Axler, J., Kim, H.J., Danese, S., et al.; GEMINI 1 Study Group (2013). Vedolizumab as induction and maintenance therapy for ulcerative colitis. N. Engl. J. Med. *369*, 699–710.

Hueber, W., Sands, B.E., Lewitzky, S., Vandemeulebroecke, M., Reinisch, W., Higgins, P.D.R., Wehkamp, J., Feagan, B.G., Yao, M.D., Karczewski, M., et al.; Secukinumab in Crohn's Disease Study Group (2012). Secukinumab, a human anti-IL-17A monoclonal antibody, for moderate to severe Crohn's disease: unexpected results of a randomised, double-blind placebo-controlled trial. Gut *61*, 1693–1700.

Jostins, L., Ripke, S., Weersma, R.K., Duerr, R.H., McGovern, D.P., Hui, K.Y., Lee, J.C., Schumm, L.P., Sharma, Y., Anderson, C.A., et al.; International IBD Genetics Consortium (IIBDGC) (2012). Host-microbe interactions have shaped the genetic architecture of inflammatory bowel disease. Nature *491*, 119–124.

Kaser, A., Zeissig, S., and Blumberg, R.S. (2010). Inflammatory bowel disease. Annu. Rev. Immunol. *28*, 573–621.

Kobayashi, K., Qiao, S.W., Yoshida, M., Baker, K., Lencer, W.I., and Blumberg, R.S. (2009). An FcRn-dependent role for anti-flagellin immunoglobulin G in pathogenesis of colitis in mice. Gastroenterology *137*, 1746–17456.e1.

Leiper, K., Martin, K., Ellis, A., Subramanian, S., Watson, A.J., Christmas, S.E., Howarth, D., Campbell, F., and Rhodes, J.M. (2011). Randomised placebo-controlled trial of rituximab (anti-CD20) in active ulcerative colitis. Gut *60*, 1520–1526.

Leppkes, M., Becker, C., Ivanov, I.I., Hirth, S., Wirtz, S., Neufert, C., Pouly, S., Murphy, A.J., Valenzuela, D.M., Yancopoulos, G.D., et al. (2009). RORgamma-expressing Th17 cells induce murine chronic intestinal inflammation via redundant effects of IL-17A and IL-17F. Gastroenterology *136*, 257–267.

Li, H., Courtois, E.T., Sengupta, D., Tan, Y., Chen, K.H., Goh, J.J.L., Kong, S.L., Chua, C., Hon, L.K., Tan, W.S., et al. (2017). Reference component analysis of single-cell transcriptomes elucidates cellular heterogeneity in human colorectal tumors. Nat. Genet. *49*, 708–718.

Ligumsky, M., Simon, P.L., Karmeli, F., and Rachmilewitz, D. (1990). Role of interleukin 1 in inflammatory bowel disease–enhanced production during active disease. Gut *31*, 686–689.

Lu, L.L., Chung, A.W., Rosebrock, T.R., Ghebremichael, M., Yu, W.H., Grace, P.S., Schoen, M.K., Tafesse, F., Martin, C., Leung, V., et al. (2016). A functional role for antibodies in tuberculosis. Cell *167*, 433–443.e14.

Ma, T.K., McAdoo, S.P., and Tam, F.W. (2017). Targeting the tyrosine kinase signalling pathways for treatment of immune-mediated glomerulonephritis: from bench to bedside and beyond. Nephrol. Dial. Transplant. *32* (suppl_1), i129–i138.

Macpherson, A., Khoo, U.Y., Forgacs, I., Philpott-Howard, J., and Bjarnason, I. (1996). Mucosal antibodies in inflammatory bowel disease are directed against intestinal bacteria. Gut *38*, 365–375.

Marchiando, A.M., Ramanan, D., Ding, Y., Gomez, L.E., Hubbard-Lucey, V.M., Maurer, K., Wang, C., Ziel, J.W., van Rooijen, N., Nuñez, G., et al. (2013). A deficiency in the autophagy gene Atg16L1 enhances resistance to enteric bacterial infection. Cell Host Microbe *14*, 216–224.

Masuda, A., Yoshida, M., Shiomi, H., Ikezawa, S., Takagawa, T., Tanaka, H., Chinzei, R., Ishida, T., Morita, Y., Kutsumi, H., et al. (2008). Fcgamma receptor regulation of Citrobacter rodentium infection. Infect. Immun. *76*, 1728–1737.

McGovern, D.P., Kugathasan, S., and Cho, J.H. (2015). Genetics of inflammatory bowel diseases. Gastroenterology *149*, 1163–1176.e2.

Neurath, M.F. (2014). Cytokines in inflammatory bowel disease. Nat. Rev. Immunol. *14*, 329–342.

Nimmerjahn, F., and Ravetch, J.V. (2008). Fcgamma receptors as regulators of immune responses. Nat. Rev. Immunol. *8*, 34–47.

Olsen, J., Gerds, T.A., Seidelin, J.B., Csillag, C., Bjerrum, J.T., Troelsen, J.T., and Nielsen, O.H. (2009). Diagnosis of ulcerative colitis before onset of inflammation by multivariate modeling of genome-wide gene expression data. Inflamm. Bowel Dis. *15*, 1032–1038.

Planell, N., Lozano, J.J., Mora-Buch, R., Masamunt, M.C., Jimeno, M., Ordás, I., Esteller, M., Ricart, E., Piqué, J.M., Panés, J., and Salas, A. (2013). Transcriptional analysis of the intestinal mucosa of patients with ulcerative colitis in remission reveals lasting epithelial cell alterations. Gut *62*, 967–976.

Reinecker, H.C., Steffen, M., Witthoeft, T., Pflueger, I., Schreiber, S., MacDermott, R.P., and Raedler, A. (1993). Enhanced secretion of tumour necrosis factor-alpha, IL-6, and IL-1 beta by isolated lamina propria mononuclear cells from patients with ulcerative colitis and Crohn's disease. Clin. Exp. Immunol. *94*, 174–181.

Sandborn, W.J., Gasink, C., Gao, L.L., Blank, M.A., Johanns, J., Guzzo, C., Sands, B.E., Hanauer, S.B., Targan, S., Rutgeerts, P., et al.; CERTIFI Study Group (2012). Ustekinumab induction and maintenance therapy in refractory Crohn's disease. N. Engl. J. Med. *367*, 1519–1528.

Schroder, K., and Tschopp, J. (2010). The inflammasomes. Cell *140*, 821–832.

Seo, S.-U., Kamada, N., Muñoz-Planillo, R., Kim, Y.-G., Kim, D., Koizumi, Y., Hasegawa, M., Himpsl, S.D., Browne, H.P., Lawley, T.D., et al. (2015). Distinct commensals induce interleukin-1β via NLRP3 inflammasome in inflammatory monocytes to promote intestinal inflammation in response to injury. Immunity *42*, 744–755.

Shafir, M., Martinelli, G., Bekesi, J.G., Jaramillo, S., Heimann, T., and Aufses, A.H., Jr. (1986). Immunoglobulins (Ig) in circulating immune complexes (CIC) in cancer and inflammatory bowel disease (IBD). Eur. J. Surg. Oncol. *12*, 351–357.

Shaw, M.H., Kamada, N., Kim, Y.G., and Núñez, G. (2012). Microbiota-induced IL-1β, but not IL-6, is critical for the development of steady-state TH17 cells in the intestine. J. Exp. Med. *209*, 251–258.

Smith, K.G., and Clatworthy, M.R. (2010). FcgammaRIIB in autoimmunity and infection: evolutionary and therapeutic implications. Nat. Rev. Immunol. *10*, 328–343.

Tamoutounour, S., Henri, S., Lelouard, H., de Bovis, B., de Haar, C., van der Woude, C.J., Woltman, A.M., Reyal, Y., Bonnet, D., Sichien, D., et al. (2012). CD64 distinguishes macrophages from dendritic cells in the gut and reveals

the Th1-inducing role of mesenteric lymph node macrophages during colitis. Eur. J. Immunol. *42*, 3150–3166.

Thomas, M.G., Bayliss, C., Bond, S., Dowling, F., Galea, J., Jairath, V., Lamb, C., Probert, C., Timperley-Preece, E., Watson, A., et al. (2019). Trial summary and protocol for a phase II randomised placebo-controlled double-blinded trial of Interleukin 1 blockade in Acute Severe Colitis: the IASO trial. BMJ Open *9*, e023765.

Uo, M., Hisamatsu, T., Miyoshi, J., Kaito, D., Yoneno, K., Kitazume, M.T., Mori, M., Sugita, A., Koganei, K., Matsuoka, K., et al. (2013). Mucosal CXCR4+ IgG plasma cells contribute to the pathogenesis of human ulcerative colitis through FcγR-mediated CD14 macrophage activation. Gut *62*, 1734–1744.

Vanhove, W., Peeters, P.M., Staelens, D., Schraenen, A., Van der Goten, J., Cleynen, I., De Schepper, S., Van Lommel, L., Reynaert, N.L., Schuit, F., et al. (2015). Strong upregulation of AIM2 and IFI16 inflammasomes in the mucosa of patients with active inflammatory bowel disease. Inflamm. Bowel Dis. *21*, 2673–2682.

Weersma, R.K., Crusius, J.B., Roberts, R.L., Koeleman, B.P., Palomino-Morales, R., Wolfkamp, S., Hollis-Moffatt, J.E., Festen, E.A., Meisneris, S., Heijmans, R., et al. (2010). Association of FcgR2a, but not FcgR3a, with inflammatory bowel diseases across three Caucasian populations. Inflamm. Bowel Dis. *16*, 2080–2089.

Weinblatt, M.E., Kavanaugh, A., Genovese, M.C., Musser, T.K., Grossbard, E.B., and Magilavy, D.B. (2010). An oral spleen tyrosine kinase (Syk) inhibitor for rheumatoid arthritis. N. Engl. J. Med. *363*, 1303–1312.

West, N.R., Hegazy, A.N., Owens, B.M.J., Bullers, S.J., Linggi, B., Buonocore, S., Coccia, M., Görtz, D., This, S., Stockenhuber, K., et al.; Oxford IBD Cohort Investigators (2017). Oncostatin M drives intestinal inflammation and predicts response to tumor necrosis factor-neutralizing therapy in patients with inflammatory bowel disease. Nat. Med. *23*, 579–589.

Willcocks, L.C., Smith, K.G., and Clatworthy, M.R. (2009). Low-affinity Fcgamma receptors, autoimmunity and infection. Expert Rev. Mol. Med. *11*, e24.

Withers, D.R., Hepworth, M.R., Wang, X., Mackley, E.C., Halford, E.E., Dutton, E.E., Marriott, C.L., Brucklacher-Waldert, V., Veldhoen, M., Kelsen, J., et al. (2016). Transient inhibition of ROR-γt therapeutically limits intestinal inflammation by reducing TH17 cells and preserving group 3 innate lymphoid cells. Nat. Med. *22*, 319–323.

Yang, S.K., Jung, Y., Kim, H., Hong, M., Ye, B.D., and Song, K. (2011). Association of FCGR2A, JAK2 or HNF4A variants with ulcerative colitis in Koreans. Dig. Liver Dis. *43*, 856–861.

Zheng, G.X.Y., Terry, J.M., Belgrader, P., Ryvkin, P., Bent, Z.W., Wilson, R., Ziraldo, S.B., Wheeler, T.D., McDermott, G.P., Zhu, J., et al. (2017). Massively parallel digital transcriptional profiling of single cells. Nat. Commun. *8*, 14049.

STAR★METHODS

KEY RESOURCES TABLE

REAGENT or RESOURCE	SOURCE	IDENTIFIER
Antibodies		
Anti-mouse B220 antibody (RA3-6B2)	Thermo Fisher Scientific	Cat#25-0452-82; RRID: AB_469627
Anti-mouse CD3 antibody (145-2C11)	Thermo Fisher Scientific	Cat#11-0031-82; RRID: AB_464882
Anti-mouse CD4 antibody (GK1.5)	Thermo Fisher Scientific	Cat#47-0041-82; RRID: AB_11218896
Anti-mouse CD11b antibody (M1/70)	Thermo Fisher Scientific	Cat#11-0112-82; RRID: AB_464935
Anti-mouse CD11c antibody (N418)	Thermo Fisher Scientific	Cat#25-0114-82; RRID: AB_469590
Anti-mouse CD138 antibody (DL-101)	Thermo Fisher Scientific	Cat#12-1389-42; RRID: AB_10548508
Anti-mouse CD19 antibody (6D5)	Biolegend	Cat#115543; RRID: AB_11218994
Anti-mouse CD38 antibody (90)	Thermo Fisher Scientific	Cat#17-0381-82; RRID: AB_469382
Anti-mouse CD45.2 antibody (104)	Thermo Fisher Scientific	Cat#A14736; RRID: AB_2534252
Anti-mouse CX3CR1 antibody (SA011F11)	Biolegend	Cat#149005; RRID: AB_2564314
Anti-mouse EpCAM antibody (G8.8)	Thermo Fisher Scientific	Cat#17-5791-82; RRID: AB_2716944
Anti-mouse F4/80 antibody (BM8)	Thermo Fisher Scientific	Cat#11-4801-82; RRID: AB_2637191
Anti-mouse FcγRI antibody (X54-5/7)	Biolegend	Cat#139309; RRID: AB_2562694
Anti-mouse FcγRIIB antibody (AT130-2)	Thermo Fisher Scientific	Cat#12-0321-82; RRID: AB_2572557
Anti-mouse FcγRIII antibody (275003)	R&D systems	Cat#FAB19601A-025; RRID: AB_664075
Anti-mouse FcγRIV antibody (9E9)	Biolegend	Cat#149503; RRID: AB_2565810
Anti-mouse IgA antibody	SouthernBiotech	Cat#1040-01; RRID: AB_2314669
Anti-mouse IgA-HRP secondary antibody	SouthernBiotech	Cat#1040-05; RRID: AB_2714213
Anti-mouse IgD antibody (11-26)	Thermo Fisher Scientific	Cat#46-5993-82; RRID: AB_2573821
Anti-mouse IgG antibody	SouthernBiotech	Cat#1037-01
Anti-mouse IgG-HRP secondary antibody	SouthernBiotech	Cat#1037-05
Anti-mouse IgG-HRP secondary antibody	Dako	Cat#P0260; RRID: AB_2636929
Anti-mouse IgG antibody	SouthernBiotech	Cat#1030-02
Anti-mouse IgG1 antibody (A85-1)	BD Biosciences	Cat#562026; RRID: AB_10926376
Anti-mouse IgG2b antibody (R12-3)	BD Biosciences	Cat#553393; RRID: AB_394831
Anti-mouse IgG3 antibody (R40-82)	BD Biosciences	Cat#565808; RRID: AB_2739364
Anti-mouse IgM antibody (II/41)	Thermo Fisher Scientific	Cat#46-5790-82; RRID: AB_1834435
Anti-mouse IL-17A antibody (TC11-18H10.1)	Biolegend	Cat#506903; RRID: AB_315463
Anti-mouse IL-22 antibody (IL22JOP)	Thermo Fisher Scientific	Cat#17-7222-82; RRID: AB_10597583
Anti-mouse Ki67 antibody (SolA15)	Thermo Fisher Scientific	Cat#11-5698-82; RRID: AB_11151330
Anti-mouse Ly6C antibody (HK1.4)	Thermo Fisher Scientific	Cat#45-5932-82; RRID: AB_2723343
Anti-mouse Ly6C/G antibody (RB6-8C5)	Thermo Fisher Scientific	Cat#47-5931-82; RRID: AB_1518804
Anti-mouse MHC-II (I-A/I-E) antibody (M5/114.15.2)	Thermo Fisher Scientific	Cat#62-5321-82; RRID: AB_2688070
Anti-mouse pro-IL-1β antibody (NJTEN3)	Thermo Fisher Scientific	Cat#17-7114-80; RRID: AB_10670739
Anti-mouse TCR beta antibody (H57-597)	Thermo Fisher Scientific	Cat#48-5961-82; RRID: AB_11039532
Anti-mouse TCR gamma/delta antibody (GL3)	Biolegend	Cat#118101; RRID: AB_313826
Anti-human CD14 antibody (61D3)	Thermo Fisher Scientific	Cat#11-0149-42; RRID: AB_10597597
Anti-human CX3CR1 antibody (2A9-1)	Thermo Fisher Scientific	Cat#12-6099-42; RRID: AB_10852707
Anti-human FcγRI antibody (10.1)	Thermo Fisher Scientific	Cat#CD6405; RRID: AB_2536514
Anti-human FcγRIIA antibody	R&D systems	Cat#AF1875; RRID: AB_2103591
Anti-human FcγRIIA/B antibody (CD32)	Thermo Fisher Scientific	Cat#46-0329-42; RRID: AB_11218874
Anti-human FcγRIIIA/B antibody (3BioCB16)	Thermo Fisher Scientific	Cat#25-0168-42; RRID: AB_10714839
Anti-human IgA1/2 antibody (G20-359)	BD Biosciences	Cat#555884; RRID: AB_396196
Anti-human IgG antibody (HP6017)	Biolegend	Cat#409304; RRID: AB_10895907

(*Continued on next page*)

Continued		
REAGENT or RESOURCE	SOURCE	IDENTIFIER
Anti-GFP antibody	Abcam	Cat#ab1218; RRID: AB_298911
Anti-goat IgG-HRP secondary antibody	Santa Cruz Biotechnology	Cat#sc-2056; RRID: AB_631730
Anti-human ASC antibody	Adipogen	Cat#AG-25B-0006; RRID: AB_2490440
Anti-human β-actin HRP-conjugated antibody	Santa Cruz Biotechnology	Cat#sc-47778 HRP; RRID: AB_2714189
Anti-human COXIV antibody	LI-COR	Cat#926-42214; RRID: AB_2783000
Anti-human IL-1β antibody	R&D systems	Cat#BAF201; RRID: AB_356214
Anti-human NLRP3 antibody	Adipogen	Cat#AG-20B-0014; RRID: AB_2490202
Anti-human TRIM21 antibody	Santa Cruz Biotechnology	Cat#sc-25351; RRID: AB_628286
Anti-rabbit IgG-HRP secondary antibody	Thermo Fisher Scientific	Cat#31460; RRID: AB_228341
Human IgG	Sigma-Aldrich	Cat#I4506; RRID: AB_1163606
InVivoMab anti-mouse IL-1R1 antibody	BioXCell	Cat#BE0256; RRID: AB_2661843
Mouse IgA	Thermo Fisher Scientific	Cat#14-4762-81; RRID: AB_470125
Mouse IgG	Sigma-Aldrich	Cat#I5381; RRID: AB_1163670
Rabbit anti-*Escherichia coli/Enterobactericeae* antibody	Abcam	Cat#ab137967
Goat anti-mouse IgG-HRP secondary antibody	Thermo Fisher Scientific	Cat#31430; RRID: AB_228307
Bacterial and Virus Strains		
L. johnsonii	Dr. S Clare	N/A
L. reuteri	Dr. S Clare	N/A
P. distasonis	Dr. S Clare	N/A
P. mirabilis	Dr. S Clare	N/A
Biological Samples		
Healthy adult peripheral blood mononuclear cells *FCGR2A* SNP study	National Blood Service	N/A
Healthy adult blood leukocyte cone	National Blood Service	Cat#NC24
Human UC and healthy control stool samples	Cambridge University Hospitals NHS Foundation Trust	N/A
Ileal tissue from healthy deceased donors	NHSBT	N/A
Chemicals, Peptides, and Recombinant Proteins		
ATP	Sigma-Aldrich	Cat#A2383-1G
MitoTEMPO	Sigma-Aldrich	Cat#SML0737-5MG
Recombinant mouse GM-CSF	Peprotech	Cat#315-03
Recombinant mouse M-CSF	Peprotech	Cat#315-02
Recombinant human M-CSF	Peprotech	Cat#300-25
Endograde ovalbumin	Hyglos	Cat#300029
Rabbit anti-chicken egg albumin antibody	Sigma-Aldrich	Cat#C6534-2ML
Brefeldin A	Thermo Fisher Scientific	Cat#00-4506-51
LIVE/DEAD Fixable Aqua Dead Cell Stain	Thermo Fisher Scientific	Cat#L34957
Flagellin from *S. typhimurium*	InvivoGen	Cat#tlrl-stfla
LPS from *E. coli* (O111:B4)	Sigma-Aldrich	Cat#L3024
Dextran sodium sulfate	MP Biomedicals	Cat#0216011080
Percoll GE Healtcare	Sigma-Aldrich	Cat#17-0891-01
Collagenase A	Sigma-Aldrich	Cat#10103578001
DNase I from bovine pancreas	Roche	Cat#10104159001
Liberase TL	Roche	Cat#5401020001
SYBR Green	Sigma-Aldrich	Cat#S9430
TMB peroxidase substrate	BD Biosciences	Cat#555214
Histopaque 1077	Sigma-Aldrich	Cat#10771-500ML
RIPA buffer	Sigma-Aldrich	Cat#R0278-500ML
Protease Inhibitor Cocktail	Roche	Cat#4693159001
Phalloidin dye	Thermo Fisher Scientific	Cat#A22287

(*Continued on next page*)

Continued

REAGENT or RESOURCE	SOURCE	IDENTIFIER
Critical Commercial Assays		
Intracellular Fixation and Permeabilization Buffer Set	Thermo Fisher Scientific	Cat#88-8824-00
BugBuster 10X Protein Extraction Reagent	Novagen	Cat#70921-10ML
High Capacity RNA-to-cDNA kit	Applied Biosystems	Cat#4387406
Mouse CXCL1 ELISA kit	R&D systems	Cat#DY453-05
Mouse CXCL2 ELISA kit	R&D systems	Cat#DY452-05
Mouse GM-CSF ELISA kit	R&D systems	Cat#DY415-05
Mouse IL-1β ELISA kit	R&D systems	Cat#DY401-05
Mouse IL-17 ELISA kit	R&D systems	Cat#DY421-05
Mouse IL-22 ELISA kit	R&D systems	Cat#DY582-05
Human IL-1β ELISA kit	R&D systems	Cat#DY201-05
Pierce BCA Protein Assay	Thermo Fisher Scientific	Cat#23227
Pierce Protein G Chromatography Cartridges	Thermo Fisher Scientific	Cat#89926
PureLink RNA Mini kit	Thermo Fisher Scientific	Cat#12183025
Quick-DNA Universal kit	Zymo Research	Cat#R4069
RNeasy Micro kit	QIAGEN	Cat#74004
ROX Low KAPPA Library Quantification kit	KAPPA Biosystems	Cat#KK4873
SMARTer stranded total RNA-Seq mammalian pico input kit	Takara	Cat#635007
SNP Genotyping Assay (*FCGR2A* rs1801274)	Thermo Fisher Scientific	C___9077561_20
TaqMan Genotyping Master Mix	Thermo Fisher Scientific	Cat#4371355
TaqMan Fast Advanced Master Mix	Thermo Fisher Scientific	Cat#4444557
TaqMan Gene Expression (*Ccl2*)	Thermo Fisher Scientific	Mm00441242_m1
TaqMan Gene Expression (*Csf2*)	Thermo Fisher Scientific	Mm01290062_m1
TaqMan Gene Expression (*Cxcl1*)	Thermo Fisher Scientific	Mm04207460_m1
TaqMan Gene Expression (*Cxcl2*)	Thermo Fisher Scientific	Mm00436450_m1
TaqMan Gene Expression (*Fcer1g*)	Thermo Fisher Scientific	Mm02343757_m1
TaqMan Gene Expression (*Fcgr1*)	Thermo Fisher Scientific	Mm00438874_m1
TaqMan Gene Expression (*Fcgr2b*)	Thermo Fisher Scientific	Mm00438875_m1
TaqMan Gene Expression (*Fcgr3*)	Thermo Fisher Scientific	Mm00438882_m1
TaqMan Gene Expression (*Fcgr4*)	Thermo Fisher Scientific	Mm00519988_m1
TaqMan Gene Expression (*Gapdh*)	Thermo Fisher Scientific	Mm99999915_g1
TaqMan Gene Expression (*Hprt*)	Thermo Fisher Scientific	Mm03024075_m1
TaqMan Gene Expression (*Ifng*)	Thermo Fisher Scientific	Mm01168134_m1
TaqMan Gene Expression (*Il1b*)	Thermo Fisher Scientific	Mm00434228_m1
TaqMan Gene Expression (*Il6*)	Thermo Fisher Scientific	Mm00446190_m1
TaqMan Gene Expression (*Il10*)	Thermo Fisher Scientific	Mm01288386_m1
TaqMan Gene Expression (*Il17a*)	Thermo Fisher Scientific	Mm00439618_m1
TaqMan Gene Expression (*Il22*)	Thermo Fisher Scientific	Mm01226722_g1
TaqMan Gene Expression (*Il23a*)	Thermo Fisher Scientific	Mm00518984_m1
TaqMan Gene Expression (*Tnf*)	Thermo Fisher Scientific	Mm00443258_m1
TaqMan Gene Expression (*Tnfsf15*)	Thermo Fisher Scientific	Mm00770031_m1
TaqMan Gene Expression (*CXCL8*)	Thermo Fisher Scientific	Hs00174103_m1
TaqMan Gene Expression (*FCER1G*)	Thermo Fisher Scientific	Hs00175408_m1
TaqMan Gene Expression (*FCGR1B*)	Thermo Fisher Scientific	Hs02341825_m1
TaqMan Gene Expression (*FCGR2A*)	Thermo Fisher Scientific	Hs01013401_g1
TaqMan Gene Expression (*FCGR2B*)	Thermo Fisher Scientific	Hs01634996_s1
TaqMan Gene Expression (*FCGR3A*)	Thermo Fisher Scientific	Hs02388314_m1
TaqMan Gene Expression (*FCGR3B*)	Thermo Fisher Scientific	Hs04334165_m1

(*Continued on next page*)

Continued

REAGENT or RESOURCE	SOURCE	IDENTIFIER
TaqMan Gene Expression (*GAPDH*)	Thermo Fisher Scientific	Hs02786624_g1
TaqMan Gene Expression (*HPRT1*)	Thermo Fisher Scientific	Hs02800695_m1
TaqMan Gene Expression (*IL1B*)	Thermo Fisher Scientific	Hs01555410_m1
Deposited Data		
RNA-seq data (colonic macrophages)	This paper	GEO: GSE109040
Single cell RNA-seq (healthy human colon)	Li et al., 2017	GEO: GSE81861
Single cell RNA-seq (healthy PBMC)	Zheng et al., 2017	https://support.10xgenomics.com/single-cell-gene-expression/datasets
Microarray data (human UC biopsies)	Vanhove et al., 2015	GEO: GSE59071
Microarray data (human UC biopsies)	Planell et al., 2013	GEO: GSE38713
Microarray data (human UC biopsies)	Olsen et al., 2009	GEO: GSE9452
Microarray data (human treatment-resistant UC biopsies)	Arijs et al., 2009a; Arijs et al., 2009b	GEO: GSE16879
Microarray data (*Citrobacter rodentium* infection)	Marchiando et al., 2013	GEO: GSE49109
Microarray data (DSS-induced colitis)	Breynaert et al., 2013	GEO: GSE42768
Experimental Models: Organisms/Strains		
Mouse: C57BL/6 (B6)	Jackson Laboratories	Stock No: 000664
Mouse: *Fcgr2b*$^{-/-}$: B6.*Fcgr2b*^{tm1Ttk}/J	Dr. J Ravetch, Dr. S Bolland	Bolland and Ravetch, 2000
Mouse: B6.M-TG	Prof. KGC Smith	Brownlie et al., 2008
Mouse: *Nlrp3*$^{-/-}$: B6.*Nlrp3*$^{-/-}$	Prof. C Bryant	N/A
Mouse: CD45.1^{+}: B6.SJL-*Ptprc*^{a} *Pepc*^{b}/BoyJ	Jackson Laboratories	Stock No: 002014
Mouse: *Rag2*$^{-/-}$: B6(Cg)-*Rag2*$^{tm1.Cgn}$/J	Jackson Laboratories	Stock No: 008449
Software and Algorithms		
FlowJo	Tree Star Inc.	https://www.flowjo.com/
Gene Set Enrichment Analysis	Broad Institute	http://software.broadinstitute.org/gsea/
GraphPad Prism 6	GraphPad Software	https://www.graphpad.com/
Imaris	Bitplane	http://www.bitplane.com/
Other		
123count eBeads	Thermo Fisher Scientific	Cat#01-1234-42
Neon Transfection System	Thermo Fisher Scientific	Cat#MPK5000
Neon Pipette Tip	Thermo Fisher Scientific	Cat#MPP100
OCT embedding medium	Thermo Fisher Scientific	Cat#LAMB/OCT

CONTACT FOR REAGENT AND RESOURCE SHARING

Further information and requests for resources and reagents should be directed to and will be fulfilled by the Lead Contact, Menna R. Clatworthy (mrc38@cam.ac.uk).

EXPERIMENTAL MODEL AND SUBJECT DETAILS

Mouse strains

All mouse lines used here are on a C57BL/6 background. *Fcgr2b*$^{-/-}$ mice were kindly provided by J. Ravetch (Rockefeller University) and S. Bolland (US National Institutes of Health, US National Institute of Allergy and Infectious Diseases (NIAID)) (Bolland and Ravetch, 2000). Macrophage transgenic (M-TG) and non-transgenic (N-TG) littermate controls were kindly provided by K.G.C. Smith. FcγRIIB overexpression was achieved using a construct in which FcγRIIB mRNA was placed under the control of the human CD68 promoter. NLRP3-deficient mice were kindly provided by Clare Bryant (University of Cambridge). C57BL/6, CD45.1^{+} C57BL/6 and *Rag2*$^{-/-}$ mice were obtained from Jackson Laboratories (Margate, UK) and maintained inhouse for several generations. For the generation of bone marrow chimeras, recipient CD45.1^{+} C57BL/6 mice were lethally irradiated (2 × 5.5 G) followed by immediate tail intravenous (i.v.) injection of 2 × 10^{6} bone marrow cells from CD45.2^{+} C57BL/6 or CD45.2^{+} *Fcgr2b*$^{-/-}$ mice. Recipient mice were checked for reconstitution after 8 weeks prior to commencement of colitis experiments. For all *in vivo* colitis experiments, 6 to 14-week old sex-matched mice were used and mice were co-housed throughout the duration of experiments. Both male and

female mice were used. All M-TG and N-TG experiments were performed on littermates. In the case of non-littermate controls, mice were co-housed for at least 3 weeks prior to the initiation of experiments. Mice were maintained in specific pathogen-free conditions at a Home Office-approved facility in the UK. All procedures were carried out in accordance with the United Kingdom Animals (Scientific Procedures) Act of 1986.

Human samples

Human ileal tissue was obtained from deceased donors with prior ethical approval (REC: 15/EE/0152). Samples were obtained from 2 male and 3 female donors aged 19-77. UC (n = 6) and healthy control (n = 6) stool samples were obtained locally (Addenbrooke's Hospital, Cambridge) with prior ethical approval (REC: 05/Q0108/355) from 6 male and 6 female donors and patient disease severity was scored using the Walmsley clinical activity index (CAI) (Table S1). Peripheral blood mononuclear cells (PBMCs) were collected from blood leukocyte cones or healthy volunteers (*FCGR2A* SNP study) with prior ethical approval from the local ethics committee (REC: 08/H0308/176). Blood was obtained from 6 male and 6 female donors aged 24 to 55.

Commensal strains

Commensal species were isolated from faeces of C57BL/6 mice and grown in LB medium at 37°C in a shaking incubator. *Parabacteroides distasonis* was grown in static culture in LB medium in an anaerobic cabinet.

METHOD DETAILS

DSS-induced experimental colitis

Colitis was induced the addition of 2% (*w/v*) 36,000-50,000MW DSS (MP Biomedicals) to drinking water for 6 days (acute DSS (aDSS)). Mice were culled at various time points up to day 28 following aDSS, as described in the text/figure legends. In some experiments, mice were subjected to a second 6-day course of 2% DSS after a two-week interval, termed chronic DSS (cDSS). Antibody was administered *in vivo* via i.p. injection (final volume, 200 μL sterile PBS). 1 mg InVivoMab anti-IL-1R1 IgG (JAMA-147; BioXCell) was given on day 0 and day 7 of a aDSS protocol. 0.5 mg anti-flagellin or control serum IgG was injected on day 0 of aDSS protocol. 0.5 mg rabbit anti-*Escherichia coli/Enterobactericeae* IgG (Abcam, ab137967) or PBS was injected on day 0 and day 3 of aDSS protocol. Colitis severity was monitored daily through changes in body weight, stool consistency, and intestinal hemorrhage. Moderate severity limits were imposed, with 20% weight loss or two moribund characteristics judged to be the severity threshold. At experimental endpoints, colon, spleen, MLN and blood were harvested and colitis severity further assessed through morphological changes in organs. The spleen, MLN, and colon were weighed, and colon length measured from cecum to rectum to determine length. The tissues were then processed for histology, RNA extraction, or flow cytometric analysis.

Flagellin immunization

Flagellin immunization was carried out as previously described (Kobayashi et al., 2009). Briefly, C57BL/6 mice were hyperimmunized by two intraperitoneal (i.p.) injections with 10 μg of flagellin from *Salmonella typhimurium* (InvivoGen) in Incomplete Freund's Adjuvant (IFA) (Sigma Aldrich) two weeks apart. Mice were culled at four weeks and total serum IgG, as well as control serum IgG from unimmunized mice, was purified using Pierce Protein G purification columns (Thermo Fisher Scientific), as per the manufacturer's instructions.

Murine primary cell isolation

Spleen, MLN, and colon were harvested and processed for single cell suspensions. Colons were dissociated from fat and luminal contents were gently removed. Tissues were opened longitudinally, cut into 0.5 cm pieces and washed by vortexing in ice-cold PBS with 10 mM HEPES. Tissue pieces were subsequently incubated with a stripping solution (RPMI-1640 medium containing 2% (*v/v*) FCS, 10 mM HEPES, 1 mM DTT, and 5 mM EDTA) at 37°C for two intervals of 20 min to remove epithelial cells, prior to enzymatic digestion in RPMI-1640 medium containing 1 mg/mL collagenase A (Sigma Aldrich) and 60 μg/mL DNase I (Roche). Tissue suspensions were mechanically dissociated and passed through a 70 μm cell strainer. Intestinal single cell suspensions were then harvested at the interface of a 40/80% (*v/v*) Percoll (Sigma-Aldrich) gradient and washed thoroughly in ice-cold PBS containing 3% (*v/v*) FCS before proceeding to further analysis. MLN and spleen suspensions were harvested by enzymatic digestion and mechanical tissue dissociation through a 70 μm filter. Splenic suspensions were subjected to red blood cell lysis (distilled H_2O containing 0.83% (*w/v*) NH_4Cl, 0.1% (*w/v*) $NaHCO_3$, 100 μM EDTA) prior to washing twice in ice-cold PBS for analysis.

Human primary cell isolation

Human LPMC isolation was carried out in a similar way to murine LPMC isolation. Tissue samples were opened longitudinally and cleaned of luminal contents. The mucosa was manually dissociated from the muscular layers, cut into small pieces and washed by vortexing in ice-cold PBS containing 10 mM HEPES. Epithelial cells and intraepithelial lymphocytes were removed by incubation in stripping buffer, as above, at 37°C for 1 h. The underlying tissues were then enzymatically digested for 1 h at 37°C in RPMI-1640 medium containing 0.42 mg/mL Liberase (Roche) or 1 mg/mL Collagenase A (Sigma-Aldrich), and 60 ug/mL DNase I (Roche), vortexed, and mechanically dissociated using a Gentle-MACS machine (Miltenyi Biotech). Dissociated tissue was then passed through a 70 μm cell strainer and centrifuged for 10 min at 1300 rpm. Intestinal single cell suspensions were harvested at the interface

of a 40/80% (*v/v*) Percoll gradient and washed twice in ice-cold PBS containing 3% (*v/v*) FCS before proceeding to downstream applications.

Flow cytometry

Single cell suspensions were blocked with 0.5% (*v/v*) heat-inactivated mouse serum followed by extracellular staining for 1 h at 4°C with a combination of the following antibodies. Murine antibodies: B220 (RA3-6B2, Thermo Fisher Scientific), CD3ε (145-2C11, Thermo Fisher Scientific), CD4 (GK1.5, Thermo Fisher Scientific), CD11b (M1/70, Thermo Fisher Scientific), CD11c (N418, Thermo Fisher Scientific), CD138 (DL-101, Thermo Fisher Scientific), CD19 (6D5, Biolegend), CD38 (90, Thermo Fisher Scientific), CD45.2 (104, Thermo Fisher Scientific), CX3CR1 (SA011F11, Biolegend), EpCAM (G8.8, Thermo Fisher Scientific), F4/80 (BM8, Thermo Fisher Scientific), FcγRI (X54-5/7, Biolegend), FcγRIIB (AT130-2, Thermo Fisher Scientific), FcγRIII (275003, R&D systems), FcγRIV (9E9, Biolegend), IgA (SouthernBiotech), IgD (11-26, Thermo Fisher Scientific), IgG (SouthernBiotech), IgG1 (A85-1, BD Biosciences), IgG2b (R12-3, BD Biosciences), IgG3 (R40-82, BD Biosciences), IgM (II/41, Thermo Fisher Scientific), Ly6C (HK1.4, Thermo Fisher Scientific), Ly6C/G (RB6-8C5, Thermo Fisher Scientific), MHC-II (M5/114.15.2, Thermo Fisher Scientific), TCR beta (H57-597, Thermo Fisher Scientific), and TCR gamma/delta (GL3, Biolegend). Human antibodies: CD14 (61D3, Thermo Fisher Scientific), CX3CR1 (2A9-1, Thermo Fisher Scientific), FcγRI (10.1, Thermo Fisher Scientific), FcγRIIA/B (CD32, Thermo Fisher Scientific), FcγRIIIA/B (eBioCB16, Thermo Fisher Scientific), IgA1/2 (G20-359, BD biosciences), and IgG (HP6017, Biolegend). Antibodies were used at a dilution of 1:200 in PBS. Viability staining was performed with LIVE/DEAD Fixable Aqua Dead Cell Stain kit (Thermo Fisher Scientific) for 20 min at room temperature. For biotinylated primary antibodies, secondary staining with streptavidin-conjugated PE (Thermo Fisher Scientific) or APC-eFluor780 (Thermo Fisher Scientific) was performed for 20 min at 4°C at a dilution of 1:300 in PBS. For intracellular cytokine staining, cells were incubated in RPMI-1640 medium containing 10% FCS, 1X penicillin-streptomycin (both Sigma-Aldrich), and 1X Brefeldin A (Thermo Fisher Scientific) solution for 3 h at 37°C, prior to fixation and permeabilization using the Intracellular Fixation and Permeabilization Buffer Set (Thermo Fisher Scientific) as per the manufacturer's instructions. Staining was carried out for 1 h at room temperature using a combination of the following antibodies: IL-17A (TC11-18H10.1, Biolegend), IL-22 (IL22JOP, Thermo Fisher Scientific), and pro-IL-1β (NJTEN3, Thermo Fisher Scientific). All antibodies were used at a 1:100 dilution. Cell counting was performed using 123count eBeads (Thermo Fisher Scientific). Flow cytometry data collection was performed on a Fortessa cytometer (BD biosciences) and data was analyzed using FlowJo software (Tree Star Inc.).

Flow sorting

Murine intestinal macrophages were flow-sorted as live CD11b$^+$ CX3CR1$^+$ Ly6C^{lo} MHC-II^{hi} cells. Human ileal macrophages were flow-sorted as SSC^{int} CX3CR1$^+$ CD14$^+$ cells. Cell sorting was performed on FACS Aria Fusion (BD biosciences), iCyt Synergy (Sony Biotechnology Inc.), and MoFlo (Beckman Coulter) cell sorters. Data were analyzed using FlowJo software (Tree Star Inc.).

Microbial flow cytometry

For murine stool samples, colonic fecal contents were homogenized in sterile PBS, briefly centrifuged at 1,000 rpm to remove large aggregates, and the resulting supernatant was washed twice in sterile PBS by centrifugation for 1 min at 8,000 rpm. At harvesting of commensal isolates, bacterial cultures were centrifuged for 10 min at 4000 rpm and used for downstream analyses. For all samples, bacterial pellets were resuspended in sterile PBS containing 1:50 dilution of mouse serum or PBS alone in 96-well v-bottom plates (Thermo Fisher Scientific) and incubated for 20 min at 4°C. For transgenic mouse serum anti-commensal IgG comparison, stool from *Rag2*-deficient mice was used, which lacks endogenous anti-commensal IgG. Human stool samples were processed as above, plated in 96-well v-bottom plates (Thermo Fisher Scientific) in sterile PBS, and centrifuged at 3,000 rpm for 10 min in a bench-top Sorvall centrifuge. For both human and murine samples, bacterial pellets or serum-opsonized bacteria were then resuspended in 50 μL sterile ice-cold PBS containing anti-mouse/human IgA-PE or anti-mouse/human IgG-Alexa 647 antibodies and stained for 20 min at 4°C. Cells were washed and resuspended in PBS or fixative containing 1:10,000 SYBR Green (Thermo Fisher Scientific) and analyzed by flow cytometry. Fecal and serum samples were paired from the same mouse, unless otherwise stated, and SYBR Green-high events were analyzed.

Serum anti-commensal IgG ELISA

For serum anti-commensal IgG analysis, colonic fecal contents were processed as described above. Bacteria were homogenized using BugBuster 10X protein extraction reagent (Novagen), centrifuged at 20,000 *g* for 10 min, and the supernatant recovered for a crude commensal bacterial antigen preparation. Protein concentration was determined using the Pierce BCA protein assay kit (Thermo Fisher Scientific). Subsequently, 96-well Nunc ELISA plates (Thermo Fisher Scientific) were coated with 5 μg/mL commensal antigen preparation overnight at 4°C, washed extensively, and murine sera incubated in doubling dilutions for 4 h at room temperature. For small volumes of sera, samples were incubated at a 1:150 dilution. In the case of serum anti-flagellin IgG detection, 96-well Nunc plates were coated overnight with 200 ng/mL flagellin purified from *Salmonella typhimurium* (InvivoGen). Commensal antigen-specific IgG was detected using a goat anti-mouse IgG-horseradish peroxidase (HRP) conjugated antibody (Thermo Fisher Scientific, 1:10000 dilution), and TMB peroxidase substrate (BD biosciences). After 15-20 min, the reaction was quenched with 1 M Na_2SO_4 and the optical densities measured at 450 nm using a CLARIOstar spectrophotometer (BMG Labtech).

Luminal IgG ELISA

Luminal contents were extruded using bicarbonate buffer (15 mM Na_2CO_3, 35 mM $NaHCO_3$) containing cOmplete protease inhibitor (Roche). 96-well Nunc ELISA plates (Thermo Fisher Scientific) were coated with primary goat anti-murine IgG or goat anti-murine IgA antibodies (catalog numbers 1037-01 and 1040-01, respectively; SouthernBiotech) for 16 h at 4°C. Plates were extensively washed and incubated with luminal suspensions in serial dilutions for 4 h at room temperature. Bound IgG and IgA were detected using secondary goat anti-murine IgG and goat anti-murine IgA antibodies conjugated to HRP (catalog numbers 1037-05 and 1040-05, respectively; SouthernBiotech) and TMB peroxidase substrate (BD biosciences). Ig concentration was determined using a standard curve of murine IgG (I5381-5mg; Sigma-Aldrich) or murine IgA (14-4762-81; Thermo Fisher Scientific). Ig levels were normalized to total luminal protein content, as determined using the Pierce BCA protein assay kit (Thermo Fisher Scientific).

Macrophage and intestinal immune cell culture

For murine BMDMs, bone marrow was flushed from the femur and tibia of mice using ice-cold sterile PBS and the subsequent cell suspension treated with red cell lysis buffer. Treated cells were then washed in ice-cold sterile PBS. BMDMs were generated by incubation of bone marrow cells in RPMI-1640 medium containing 10% FCS and 1X penicillin-streptomycin (both Sigma-Aldrich) (referred to as complete RPMI) supplemented with 100 ng/mL murine macrophage colony-stimulating factor (M-CSF; Peprotech). M-CSF-supplemented culture medium was replaced on day 3 and BMDMs were harvested on day 5-6. BMDMs were primed with 20 ng/mL murine granulocyte-macrophage colony-stimulating factor (GM-CSF; Peprotech) for 16 h prior to stimulation. For human MDM culture, diluted blood was overlaid on Histopaque 1077 (Sigma-Aldrich) in a 50 mL centrifuge tube and centrifuged for 20 min at 2,000 rpm at room temperature. The PBMC layer was removed and resuspended in cold complete RPMI. PBMCs were incubated in complete RPMI medium supplemented with 100 ng/mL human M-CSF (Peprotech). Additional M-CSF-supplemented media was added at day 3 and adherent macrophages were harvested at day 6.

Flow-sorted intestinal MNPs were plated in complete RPMI-1640 medium at a density of 0.5-1 × 10^5 cells per well in 96-well round-bottom plates (Sigma-Aldrich) prior to stimulation. LPMC suspensions were plated in complete RPMI-1640 medium at a density of 5x10^5-1x10^6 cells per well in 24-well plates (Sigma-Aldrich) prior to *ex vivo* stimulation with Ova-IC or Ova. Cells were then washed extensively, and analyzed by qPCR, ELISA, and flow cytometry.

IgG stimulation

For IgG IC stimulation, model Ova-IC was generated by opsonization of 40 μg/mL endotoxin-free ovalbumin (Ova; Hyglos) with 1.2-2 mg/mL polyclonal rabbit anti-Ova IgG antibody in serum (Sigma-Aldrich) at 37°C for 1h. Immune cells were stimulated in complete RPMI with Ova, anti-Ova serum, or Ova-IC at 37°C in a 5% CO_2 incubator for 4 h for RNA analysis, including RNA-seq and quantitative PCR (qPCR), or 16 h for cytokine and chemokine ELISAs. For BMDM IL-1β production, 5 × 10^5 murine BMDMs were stimulated with Ova or Ova-IC for 4 h at 37°C followed by 30 min stimulation with 2.5 mM ATP (Sigma-Aldrich).

For human c-IgG stimulation, 96-well high-affinity Nunc MaxiSorp plates (Thermo Fisher Scientific) were coated with 2 μg/mL human IgG (Sigma-Aldrich) overnight at room temperature. Plates were blocked with PBS containing 10% FCS for 1 h. Macrophages were plated at 0.5-1x10^5 cells per well in triplicate in complete RPMI and additionally stimulated with 100 ng/mL LPS from *E. coli* (Sigma-Aldrich) or fecal commensal extract from healthy human donors harvested as above and outlined previously (Seo et al., 2015). Supernatants were harvested after 24 h and analyzed by ELISA. In certain experiments, macrophages were pre-incubated for 30 min at 37°C with 20 μg/mL anti-FcγRIIA blocking antibody (R&D systems) and subsequently maintained at 5 μg/mL in culture with additional stimuli. Mitochondrial ROS were blocked using 0.5 mM MitoTEMPO (Sigma-Aldrich).

Trim-Away

Human MDMs were electroporated with PBS, mouse anti-GFP (9F9.F9; Abcam) or mouse anti-NLRP3 (Cryo-2; Adipogen) IgG antibodies. All antibodies used for electroporation were passed through Amicon Ultra-0.5 100 kDa centrifugal filter devices (Millipore) to remove traces of azide and replace buffer with PBS. All antibodies were diluted to 0.6 mg/mL in PBS prior to electroporation. Antibody electroporation was performed using the Neon Transfection System (Thermo Fisher Scientific). MDMs were washed with PBS and resuspended in Buffer R (Thermo Fisher Scientific) at a concentration of 1.4 × 10^8 cells/mL. For each electroporation reaction 1.4 × 10^6 cells (10 μl) were mixed with 2 μl of antibody or PBS. The mixture was taken up into a 10 μl Neon® Pipette Tip (Thermo Fisher Scientific) and electroporated using the following settings: 1400V, 20 ms, 2 pulses. Electroporated cells were transferred to growth medium without antibiotics.

Immunoblotting

MDMs were plated and stimulated as indicated previously in serum-free medium. 500 mL of supernatants was collected and precipitated using Methanol/Chloroform extraction. 500 mL MeOH and 125 mL Chloroform was added to 500 mL cell-free supernatant, vortexed briefly and spun at 13 000 g for 5 min. The upper layer was removed and a further 500 mL MeOH added to each sample, vortexed and spun at 13 000 g for 5 min. The supernatant was completely aspirated and the protein pellet was resuspended directly in NuPAGE LDS Sample buffer with 100 mM DTT and heated at 95°C for 10 min. MDMs were lysed in RIPA buffer (CST-9806) supplemented with a protease inhibitor cocktail (Roche), spun at 14000 g for 10 min and cleared lysates mixed with NuPAGE LDS Sample Buffer and heated at 95°C for 10 min. Samples were run on NuPAGE 4%–12% Bis-Tris gels (Thermo Fisher) and transferred onto nitrocellulose membrane. Antibody incubations were performed in PBS with 5% (*v/v*) milk and 0.1% (*v/v*) Tween-20.

The primary antibodies used were goat anti-IL-1β IgG antibody (BAF201, R&D Systems, 1:500), mouse anti-NLRP3 (Cryo-2; Adipogen; 1:500), rabbit anti-ASC (AL177; Adipogen; 1:500), mouse anti-TRIM21 (D-12; Santa Cruz Biotechnology; 1:500) and rabbit anti-COXIV (LI-COR; 1:5000). HRP-coupled secondary anti-goat (Santa Cruz, sc-2056), anti-mouse (Dako), anti-rabbit (Thermo Fisher Scientific), and anti-β-actin (Sant Cruz, sc-47778) antibodies were detected by enhanced chemiluminescence (Amersham, GE Healthcare) and X-ray films.

FCGR2A genotyping

DNA was extracted from whole blood of healthy volunteers using the Quick-DNA Universal kit (Zymo Research) as per the manufacturer's instructions. Genotyping was performed using the TaqMan Genotyping Master Mix (Thermo Fisher Scientific) and SNP Genotyping Assay probes for *FCGR2A* SNP rs1801274 (Thermo Fisher Scientific) as per the manufacturer's instructions by qPCR.

RNA extraction and reverse transcription

RNA extraction was carried out using commercially available kits as per the manufacturer's instructions. QIAGEN RNeasy micro kits were used for cell numbers below 5 × 10^5. The PureLink RNA mini kit (Thermo Fisher Scientific) was used for cell numbers over 5 × 10^5. For whole tissue RNA extraction, tissue pieces were first disrupted using a Precellys 24 Homogenizer (Bertin Instruments), before extraction using the PureLink RNA mini kit (Thermo Fisher Scientific). RNA concentration and purity were determined using a NanoDrop spectrophotometer (Thermo Scientific) prior to cDNA synthesis using a High-Capacity RNA-to-cDNA kit (Applied Biosystems).

Quantitative polymerase chain reaction

All qPCR was carried out in triplicate with Taqman reagents and the following pre-designed TaqMan Gene Expression Assay primers and probes (Thermo Fisher Scientific). Murine primers: *Ccl2* (Mm00441242_m1), *Csf2* (Mm01290062_m1), *Cxcl1* (Mm04207460_m1), *Cxcl2* (Mm00436450_m1), *Fcer1g* (Mm02343757_m1), *Fcgr1* (Mm00438874_m1), *Fcgr2b* (Mm00438875_m1), *Fcgr3* (Mm00438882_m1), *Fcgr4* (Mm00519988_m1), *Gapdh* (Mm99999915_g1), *Hprt* (Mm03024075_m1), *Ifng* (Mm01168134_m1), *Il1b* (Mm00434228_m1), *Il6* (Mm00446190_m1), *Il10* (Mm01288386_m1), *Il17a* (Mm00439618_m1), *Il22* (Mm01226722_g1), *Il23a* (Mm00518984_m1), *Tnf* (Mm00443258_m1), and *Tnfsf15* (Mm00770031_m1). Human primers: *CXCL8* (Hs00174103_m1), *FCER1G* (Hs00175408_m1), *FCGR1B* (Hs02341825_m1), *FCGR2A* (Hs01013401_g1), *FCGR2B* (Hs01634996_s1), *FCGR3A* (Hs02388314_m1), *FCGR3B* (Hs04334165_m1), *GAPDH* (Hs02786624_g1), *HPRT1* (Hs02800695_m1), and *IL1B* (Hs01555410_m1). qPCR was carried performed on the Viia 7 PCR machine (Life Technologies). Gene expression was normalized to *Gapdh* or *Hprt* using the $2^{-\Delta Ct}$. The $2^{-\Delta\Delta Ct}$ method was used for normalization between experimental conditions and genotypes.

Cytokine/chemokine ELISA

Quantification of human and murine cytokines and chemokines in culture supernatants was carried out using commercially available R&D systems Duoset ELISA kits, as per the manufacturer's instructions.

Immunofluorescence

Intestinal tissues were fixed with 1% (*w/v*) paraformaldehyde (Electron Microscopy Services) in PBS for 16 h, washed with PBS, and equilibrated in 30% (*w/v*) sucrose for a further 16 h. Tissues were then frozen at −80°C in Optimal Cutting Temperature (OCT) embedding medium (Thermo Fisher Scientific). Cryostat sections were cut at a thickness of 20-30 μm, air-dried for 1 h, then rehydrated for 10 min in PBS and blocked with a 0.1 M Tris solution containing 1% (*w/v*) mouse serum, 1% (*w/v*) bovine serum albumin (BSA), and 0.1% (*w/v*) Triton X-100 for 1 h at room temperature. Sections were stained overnight at 4°C with a combination of the following antibodies in blocking buffer at a 1:100 dilution: CD45.2 (104; Thermo Fisher Scientific), IgG (SouthernBiotech), and Ki67 (SolA15; Thermo Fisher Scientific). Additionally, actin was stained in certain experiments using Phalloidin dyes (Thermo Fisher Scientific) at a 1:200 dilution. Confocal imaging was carried out on a Leica SP8 confocal microscope. Images were analyzed using Imaris software (Bitplane).

RNA-seq sample preparation

Flow-sorted CX3CR1$^+$ CD11b$^+$ Ly6C^{lo} MHC-II$^+$ macrophages were plated at a density of 1 × 10^5 cells per well in 96-well round-bottom plates and stimulated with Ova or Ova-IC for 4 h at 37°C in a 5% CO_2 incubator. Following stimulation, cells were transferred into 750 μL RLT plus buffer (QIAGEN). Samples were immediately vortexed, snap frozen on dry ice and stored at −80°C. RNA was extracted from cell lysates using the RNeasy plus micro kit (QIAGEN) as per the manufacturer's instructions. Optimal DNA depletion columns (QIAGEN) were used to remove contaminating genomic DNA. Purified RNA was eluted in nuclease free water (Ambion) and stored at −80°C. Quality and concentration of the purified RNA was assessed using an RNA pico chip (Applied Biosystems) using a Bioanalyzer 2000 (Applied Biosystems) as per the manufacturer's instructions. For all RNA-seq experiments, samples had an RNA integrity number greater than 8, indicating minimal degradation of the RNA. For the preparation of libraries, SMARTer stranded total RNA-Seq mammalian pico input kit (Takara) was used as per the manufacturer's instructions. To produce the libraries, 1.5-3.55 ng of total RNA was used and libraries were amplified for 14 cycles of PCR. Library size was assessed using 1 μL of undiluted final libraries with a High Sensitivity DNA chip (Applied Biosystems) using a Bioanalyzer 2000 (Applied Biosystems) as per the

manufacturer's instructions. Library concentration was quantified by PCR using 1/10000 dilution of the library in nuclease free water (Ambion) with ROX low KAPPA library quantification kit (KAPPA Biosystems). Libraries were pooled at an equimolar concentration with up to 12 libraries per pool.

RNA sequencing and analysis

Sequencing of the libraries was carried out using a Hiseq 2500 (Illumina) on a 2x100bp sequencing run with 1 pool per flow cell lane. Sequencing was carried out at Genewiz (NJ, USA). Pooled libraries were de-multiplexed by Genewiz using Casava (Illumina) before transfer of the data to the University of Cambridge. Fastq files were trimmed of the first 3 nucleotides of the R1 strand and contaminating adaptor sequences and poor-quality bases removed (bases with a phred 33 score of < 30) using trimgalore! (Babraham bioinformatics) and quality of the resulting files was assessed using FastQC (Babraham bioinformatics). Fastq files were aligned to the mm10 genome (Downloaded from https://genome-euro.ucsc.edu/cgi-bin/hgGateway?db=mm10&redirect=manual&source=genome.ucsc.edu on 18th January 2016) using hisat2. All analysis was carried out using R version 3.2.4. Reads were counted and assigned to genes using the Featurecount function from the RSubread package. Differential expression analysis was carried out using DESeq2 using a linear model with an appropriate design matrix following the default workflow. Resulting figures were plotted using ggplot2 and heatmap.2 from the gplots package. GSEA was performed for RNA-seq data by first assigning a rank metric to each gene using the following formula:

$$\text{Rank metric} = 1\left(\text{P value} + 1\text{x}10^{-300}\right) * (|\text{LFC}|/\text{LFC})$$

GSEA was then run using GSEA 2.1 using the pre-ranked option with the classic setting against either gene sets from the molecular signature database or custom gene sets indicated in the text. FcγR gene lists were obtained from the Molecular Signature Database (MSigDB; http://software.broadinstitute.org/gsea/msigdb). Gene ontology analysis was carried out using TopGo. Selected populations were compared for enrichment to a gene universe which contained only genes which had a possibility of being present in both UC microarray and macrophage Ova-IC RNA-seq groups. Enriched GO terms were identified using a Fisher elim method.

Microarray

Publicly available microarray datasets were downloaded from GEO (https://www.ncbi.nlm.nih.gov/geo/) along with appropriate chip annotation data. All analyses were carried out using R. All datasets were downloaded as raw intensity matrices. Data was normalized using RMA and limma. Probes were reduced to one probe per gene by selecting the probe with the greatest variance across the samples using the gene filter package. Differential expression was varied out using limma with an appropriate design matrix. GEO: GSE59071 (Vanhove et al., 2015), GEO: GSE38713 (Planell et al., 2013) and GEO: GSE9452 (Olsen et al., 2009) datasets were used for human UC analysis, GEO: GSE16879 (Arijs et al., 2009a; Arijs et al., 2009b) for infliximab-resistant UC analysis (see also Table S2), GEO: GSE42768 (Breynaert et al., 2013) for analysis of murine DSS-induced colitis, and GEO: GSE49109 for *C. rodentium* infection analysis (Marchiando et al., 2013). The *IGH* expression score was calculated as a sum of the normalized log2-transformed expression values of all *IGH*-containing probes in each sample. AUROC analysis was performed on normalized log2-transformed expression values of *FCGR2A* or the *IGH* expression score using GraphPad Prism 6. GSEA was performed as for RNA-seq without pre-ranking against either gene sets from the molecular signature database or custom gene sets indicated in the text.

Hierarchical clustering

R and the base stats package were used for all calculations. Plots were generated using the package dendextend. For clustering analysis, the RMA normalized intensity for the genes of interest (all cytokines plus *FCGR2A*) were selected and a distance matrix calculated using Euclidean distance using the function "dist." The samples were subsequently hierarchically clustered using the function "hclust" using the complete method. To define clusters, the tree was cut into k clusters at a given height based on visual interpretation of the dendrogram (typically k = 4-5). The cluster containing *FCGR2A* was examined further.

Single cell RNA-seq

Publicly available normal colonic mucosa single cell RNA-seq raw count data (Li et al., 2017) was acquired from GEO (GSE81861). Data was analyzed in R using the Seurat package. Genes with very low expression were discarded (average counts < 0.4). Data was log-normalized using global scaling, and AUROC test was used to generate a ranked list of cell type-specific genes and associated scores. Peripheral blood mononuclear cell raw count data (Zheng et al., 2017) was acquired from the 10X genomics data portal (https://support.10xgenomics.com/single-cell-gene-expression/datasets) – Donor A 68K PBMC. Data was log-normalized using global scaling in the Seurat package. Thereafter we detected highly variable genes with average log expression values between 0.01 and8 using the FindVariableGenes function in Seurat. We extracted the top 20 cell loadings after principal components analysis on the expression matrix subsetted to variable genes and generated a UMAP embedding from these loadings. We clustered the resulting k-nearest neighbor graph using Louvain clustering with default settings and annotated clusters based on marker genes defined by the AUROC test. The top 50 marker genes for each annotated cell type were used to calculate a rank-based enrichment score for single cells in the gut dataset, using the AUCell package (Aibar et al., 2017).

QUANTIFICATION AND STATISTICAL ANALYSIS

Statistical analysis was performed using GraphPad Prism software or R. For *in vivo* colitis experiments, comparison between experimental groups was performed using a nonparametric Mann-Whitney-U test, unless otherwise stated, and medians are indicated. For *in vitro* stimulation experiments, mean ± standard error of mean (SEM) are shown and a parametric Student's two-tailed t test or two-way ANOVA with Tukey's multiple comparisons test was used, unless paired samples were used, where a ratio paired t test was used. For correlations of RNA expression levels, linear regression analysis was used. For RNA-seq bioinformatics analyses, *P value*s were calculated using the standard DESeq 2 method with multiple correction using BH. For microarray experiments, *P value*s were calculated using the limma package with multiple correction using BH. * $p < 0.05$; ** $p < 0.01$; *** $p < 0.001$; **** $p < 0.0001$. Sample sizes (*n*) for all shown data can be found in the figure legends. *In vitro* stimulations were performed in triplicate, unless stated, and sample sizes for *in vivo* experiments were determined based on initial experiments.

DATA AND SOFTWARE AVAILABILITY

The accession number for the data in this paper is GEO: GSE109040.

Immunity, Volume *50*

Supplemental Information

Anti-commensal IgG Drives Intestinal Inflammation and Type 17 Immunity in Ulcerative Colitis

Tomas Castro-Dopico, Thomas W. Dennison, John R. Ferdinand, Rebeccah J. Mathews, Aaron Fleming, Dean Clift, Benjamin J. Stewart, Chenzhi Jing, Konstantina Strongili, Larisa I. Labzin, Edward J.M. Monk, Kourosh Saeb-Parsy, Clare E. Bryant, Simon Clare, Miles Parkes, and Menna R. Clatworthy

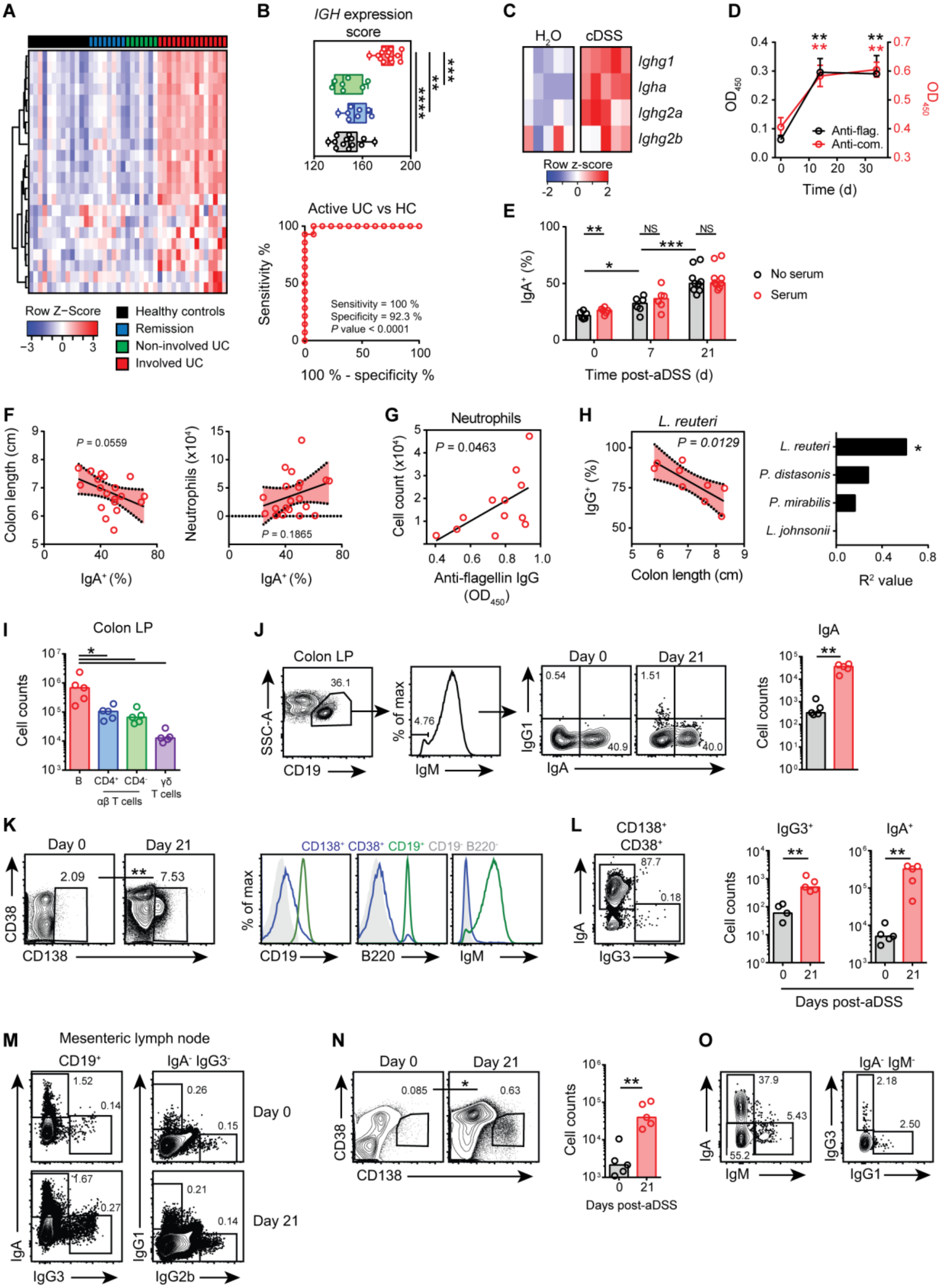

A
Row Z-Score
−3 0 3
Healthy controls
Remission
Non-involved UC
Involved UC
B
IGH expression score
120 160 200
****
**
***
Active UC vs HC
Sensitivity %
100 % - specificity %
Sensitivity = 100 %
Specificity = 92.3 %
P value < 0.0001
C
H2O
cDSS
Ighg1
Igha
Ighg2a
Ighg2b
Row z-score
-2 0 2
D
OD450
Time (d)
Anti-flag.
Anti-com.
E
IgA+ (%)
Time post-aDSS (d)
No serum
Serum
NS
F
Colon length (cm)
IgA+ (%)
P = 0.0559
Neutrophils (x10⁴)
P = 0.1865
G
Neutrophils
Cell count (x10⁴)
P = 0.0463
Anti-flagellin IgG (OD450)
H
L. reuteri
IgG+ (%)
P = 0.0129
Colon length (cm)
L. reuteri
P. distasonis
P. mirabilis
L. johnsonii
R² value
I
Colon LP
Cell counts
B
CD4+
CD4-
αβ T cells
γδ T cells
J
Colon LP
SSC-A
CD19
36.1
% of max
IgM
4.76
Day 0
Day 21
IgG1
IgA
0.54
40.9
1.51
40.0
IgA
Cell counts
K
Day 0
Day 21
CD38
CD138
2.09
7.53
CD138+ CD38+ CD19+ CD19- B220-
% of max
CD19
B220
IgM
L
CD138+ CD38+
IgA
IgG3
87.7
0.18
IgG3+
IgA+
Cell counts
Days post-aDSS
M
Mesenteric lymph node
CD19+
IgA- IgG3-
1.52
0.14
0.26
0.15
Day 0
1.67
0.27
0.21
0.14
Day 21
IgA
IgG3
IgG1
IgG2b
N
Day 0
Day 21
0.085
0.63
CD38
CD138
Cell counts
Days post-aDSS
O
IgA- IgM-
37.9
5.43
55.2
IgA
IgM
2.18
2.50
IgG3
IgG1

Figure S1. Anti-commensal Ig and B cell infiltration in intestinal inflammation, related to Figure 1. (A) Heatmap of *IGH* gene probes from transcriptomic analysis of inflamed active UC mucosal biopsies (n = 15) compared to mucosal biopsies from healthy controls (n = 13), remission (n = 8), and non-inflamed biopsies (n = 7). Data were derived from the GEO dataset GSE38713. (B) Cumulative expression score of *IGH* genes shown in A (top) and AUROC analysis of computed scores between inflamed UC biopsies and healthy control biopsies (bottom). Min. to max. box-and-whisker plots are shown for patient scoring. (C) Heatmap of *Igh* genes from transcriptomic analysis of a previously published dataset of murine DSS-inflamed colons versus healthy controls (n = 5 per group). Data were derived from the GEO dataset GSE42768. (D) Anti-mouse IgG ELISA showing *de novo* anti-commensal (red) and anti-flagellin (black) IgG generation post-aDSS (n = 5 per group). Means ± s.e.m. are indicated. Data are representative of three independent experiments. (E) Microbial flow cytometric quantification of IgA-bound SYBR green^{hi} luminal bacteria post-aDSS or in healthy controls, with (red) and without (black) paired serum pre-incubation (n = 6-9 per group). Medians are indicated. Data are pooled from two independent experiments. (F) Correlation analysis of luminal IgA-opsonized commensal microbes from the day 21 timepoint (no serum) with large intestine length (left) and neutrophil infiltration (right) (n = 20). Data are pooled from two independent experiments. (G) Correlation analysis of colonic neutrophil infiltration with systemic serum anti-flagellin IgG titers at day 28 post-aDSS (n = 12). Data are representative of two independent experiments. (H) Correlation of commensal-serum IgG opsonization levels with colon length, pooled from healthy controls and colitic mice at day 21 post-aDSS (n = 9). (I, J) Flow cytometric quantification of absolute counts of colonic lymphocyte subsets (I) and colonic IgM$^-$ class-switched CD19$^+$ B cell subsets (J) at day 21 post-aDSS administration or in healthy controls (n = 5 per group). Medians are indicated. Data are representative of two or three independent experiments. (K, L) Identification (K) and class-switch analysis (L) of colonic lamina propria CD138$^+$ CD38$^+$ plasma cell subsets in colitic mice at day 21 post-aDSS or healthy control mice (day 0) (n = 5 per group). Medians are indicated for the absolute counts of IgG3$^+$ and IgA$^+$ plasma cells in L. Data are representative of three independent experiments. (M) Profiling of MLN IgM$^-$ class-switched CD19$^+$ B cell subsets, as in J. (N, O) Identification (N) and class-switch analysis (O) of MLN CD138$^+$ CD38$^+$ plasma cells, as in K and L. Medians are indicated for absolute counts of MLN plasma cells in N. Data are representative of two or three independent experiments. P values were calculated using a Kruskal-Wallis test with Dunn's multiple comparisons test (B, top), AUROC analysis (B bottom), the nonparametric Mann-Whitney U test (D, E, J-O), linear regression analysis (F-H), or a one-way ANOVA with Tukey's multiple comparisons test (I). * P < 0.05; ** P < 0.01; *** P < 0.001; **** P < 0.0001.

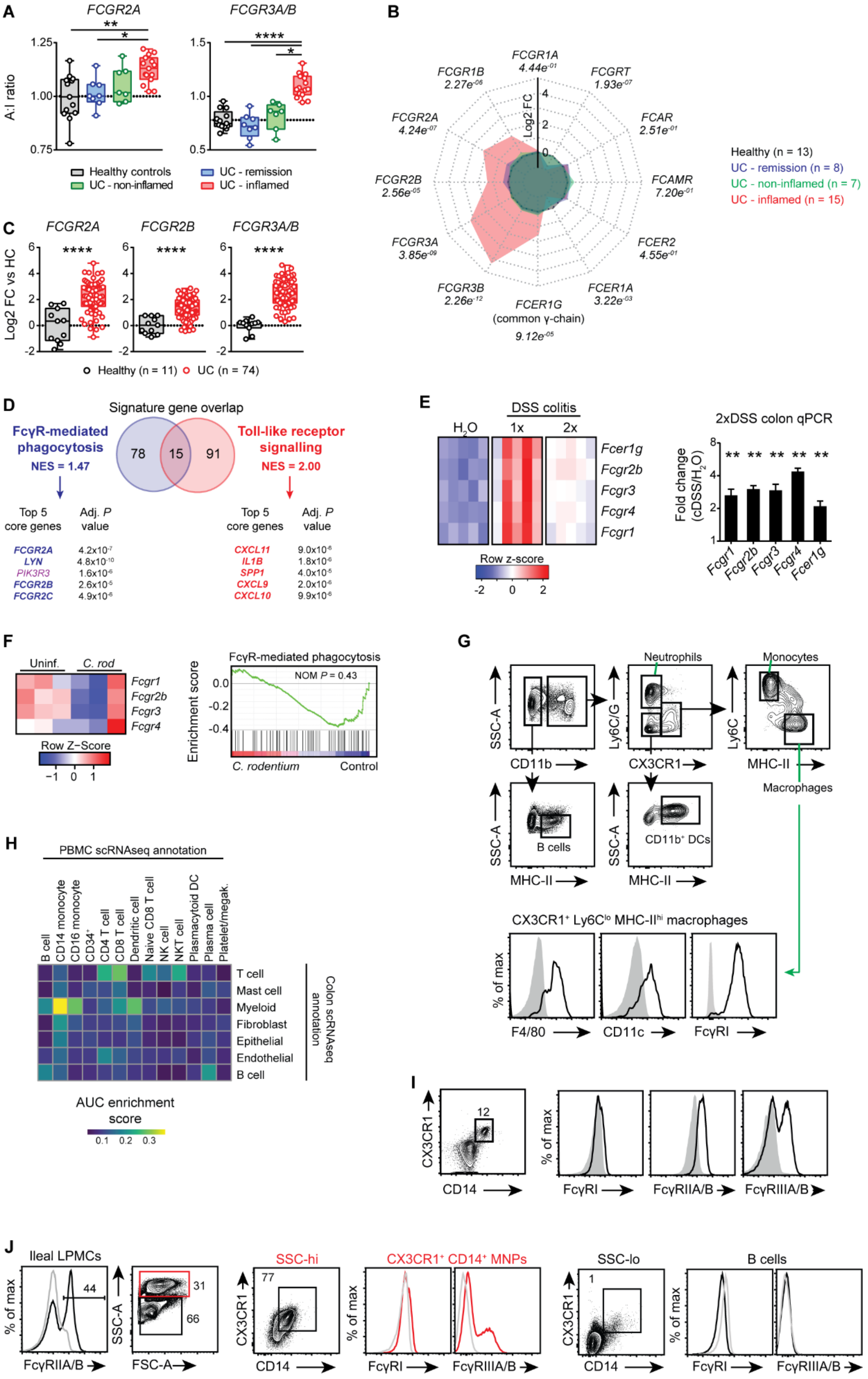
A
FCGR2A
FCGR3A/B
A:I ratio
Healthy controls
UC - remission
UC - non-inflamed
UC - inflamed
B
FCGR1A 4.44e-01
FCGRT 1.93e-07
FCAR 2.51e-01
FCAMR 7.20e-01
FCER2 4.55e-01
FCER1A 3.22e-03
FCER1G (common γ-chain) 9.12e-05
FCGR3B 2.26e-12
FCGR3A 3.85e-09
FCGR2B 2.56e-05
FCGR2A 4.24e-07
FCGR1B 2.27e-06
Log2 FC
Healthy (n = 13)
UC - remission (n = 8)
UC - non-inflamed (n = 7)
UC - inflamed (n = 15)
C
FCGR2A
FCGR2B
FCGR3A/B
Log2 FC vs HC
Healthy (n = 11)
UC (n = 74)
D
Signature gene overlap
FcγR-mediated phagocytosis
NES = 1.47
Toll-like receptor signalling
NES = 2.00
78 15 91
Top 5 core genes
Adj. P value
FCGR2A 4.2x10-7
LYN 4.8x10-10
PIK3R3 1.6x10-6
FCGR2B 2.6x10-5
FCGR2C 4.9x10-5
CXCL11 9.0x10-8
IL1B 1.8x10-6
SPP1 4.0x10-5
CXCL9 2.0x10-6
CXCL10 9.9x10-6
E
DSS colitis
H2O
1x
2x
Fcer1g
Fcgr2b
Fcgr3
Fcgr4
Fcgr1
Row z-score
2xDSS colon qPCR
Fold change (cDSS/H2O)
Fcgr1
Fcgr2b
Fcgr3
Fcgr4
Fcer1g
F
Uninf.
C. rod
Fcgr1
Fcgr2b
Fcgr3
Fcgr4
Row Z-Score
FcγR-mediated phagocytosis
NOM P = 0.43
Enrichment score
C. rodentium
Control
G
Neutrophils
Monocytes
SSC-A
CD11b
Ly6C/G
CX3CR1
Ly6C
MHC-II
Macrophages
B cells
CD11b+ DCs
CX3CR1+ Ly6Clo MHC-IIhi macrophages
% of max
F4/80
CD11c
FcγRI
H
PBMC scRNAseq annotation
B cell
CD14 monocyte
CD16 monocyte
CD34+
CD4 T cell
CD8 T cell
Dendritic cell
Naive CD8 T cell
NK cell
NKT cell
Plasmacytoid DC
Plasma cell
Platelet/megak.
T cell
Mast cell
Myeloid
Fibroblast
Epithelial
Endothelial
B cell
Colon scRNAseq annotation
AUC enrichment score
I
CX3CR1
CD14
12
% of max
FcγRI
FcγRIIA/B
FcγRIIIA/B
J
Ileal LPMCs
% of max
FcγRIIA/B
44
SSC-A
FSC-A
31
66
SSC-hi
CX3CR1+ CD14+ MNPs
SSC-lo
B cells
CX3CR1
CD14
77
1
FcγRI
FcγRIIIA/B

Figure S2. FcγR profiling in intestinal inflammation, related to Figure 2. (A) A:I ratio of *FCGR* genes in transcriptomics analysis of mucosal biopsies from inflamed active UC mucosal biopsies (n = 15) compared to mucosal biopsies from healthy controls (n = 13), remission (n = 8), and non-inflamed biopsies (n = 7). Data were derived from the GEO dataset GSE38713. Min. to max. box-and-whisker plots are shown. (B) Comparison of Fc receptor gene enrichment in IBD biopsies from dataset in A. *P* values listed below gene names. (C) Transcriptomics analysis of *FCGR* gene transcripts in intestinal mucosal biopsies from an independent previously published UC cohort. Data were derived from GEO dataset GSE59071 (UC = 74; HC = 11). Max. to min. boxplots are shown. (D) Overlap of KEGG *FcγR-mediated phagocytosis* and *Toll-like receptor signaling* pathway genes, with the top 5 core enrichment genes within inflamed mucosal biopsies from dataset in A. (E) Heatmap showing transcriptomic analysis of murine *Fcgr* genes in uninflamed (H_2O), aDSS, and cDSS colon tissue (left, n = 5 per group) and qPCR of *Fcgr* expression in the inflamed colons of mice exposed to cDSS over healthy colons by qPCR (right, n = 5 per group). Transcriptomics data was generated from the GEO dataset GSE42768. Mean ± s.e.m. are indicated. Data are representative of two independent experiments. (F) FcγR gene enrichment and GSEA of KEGG *FcγR-mediated phagocytosis* in murine colonic tissue following *C. rodentium* infection (n = 3 per group). Data was derived from the GEO dataset GSE49109. (G) Flow cytometric gating strategy for FcγR-expressing cells within the inflamed murine colonic lamina propria. Grey = isotype control. (H) Immune cell subset identification in colonic single cell RNAseq through alignment with reference PBMC single cell RNAseq dataset. Colonic data was derived from GSE81861 and PBMC data was acquired from the 10X genomics data portal. (I) Flow cytometry of FcγR expression by human ileal intestinal $CD14^+$ $CX3CR1^+$ macrophages from a healthy donor. Data are representative of two independent experiments. (J) Flow cytometric analysis of total FcγRII-expressing cells within the human ileum. Data are representative of two independent experiments. *P* values were calculated using a Kruskal-Wallis test with Dunn's multiple comparison test (A), limma with multiple correction using BH (B-D), or the non-parametric Mann-Whitney *U* test (E). * $P < 0.05$; ** $P < 0.01$; *** $P < 0.001$; **** $P < 0.0001$.

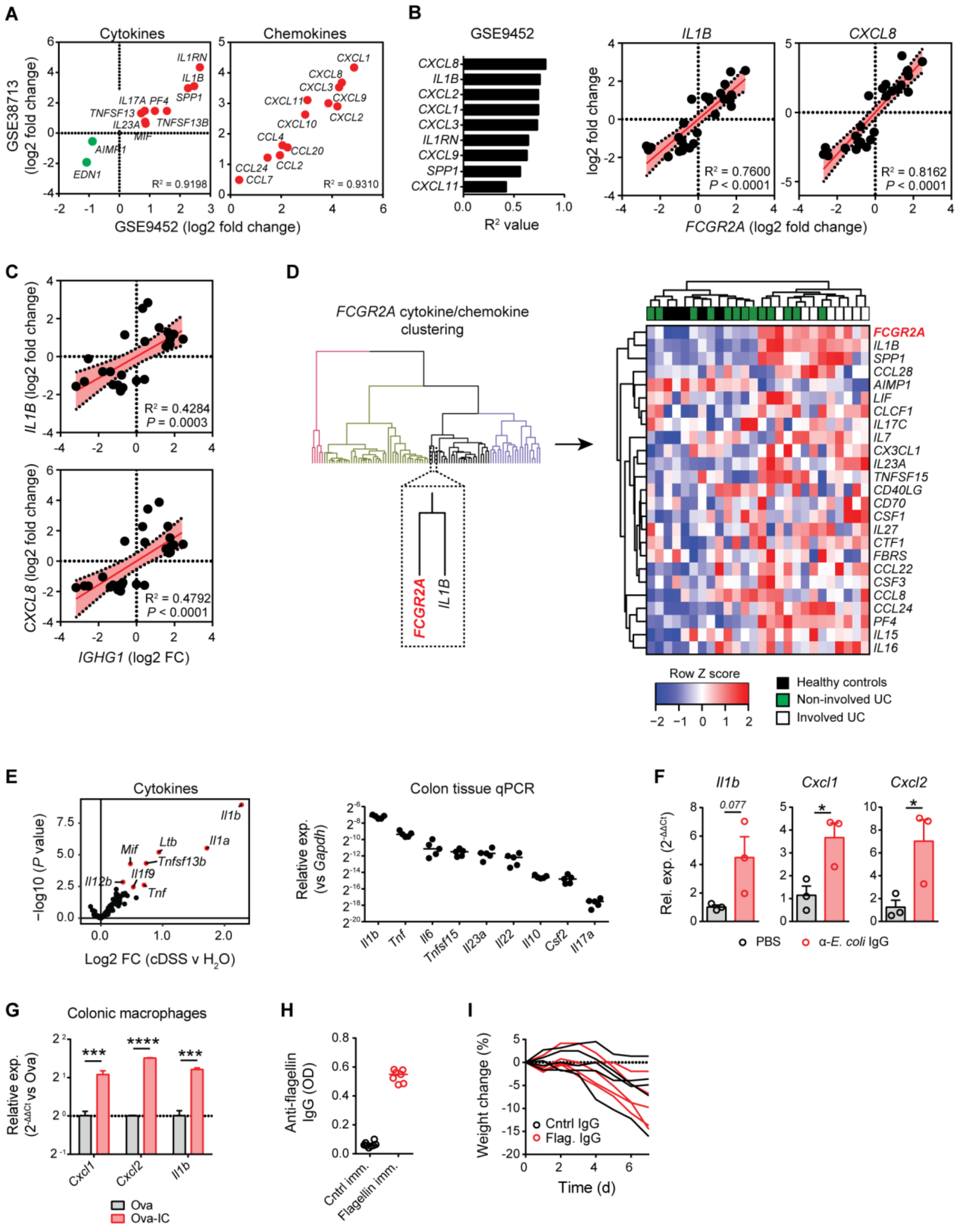

Figure S3. Mucosal FcγR expression analysis and murine DSS inflammatory networks, related to Figure 3. (A) Log2 FC comparison of significantly differentially expressed (adj. *P* val. < 0.05) cytokine and chemokine genes across two independent UC cohorts. Data derived from GEO datasets GSE38713 (UC = 15, HC = 13) and GSE9452 (UC = 8, HC = 5). (B) Correlation of *FCGR2A* expression with UC-associated cytokine/chemokine gene transcripts within

pooled mucosal biopsies from data derived from GEO dataset GSE9452. (C) Correlation of *IGHG1* expression with *IL1B* and *CXCL8* gene transcripts in colonic biopsies pooled from healthy controls (n = 5) and UC patients (n = 8). Data were derived from the GEO dataset GSE9452. (D) Hierarchical clustering of chemokine and cytokine genes with *FCGR2A* expression levels in a replication pooled dataset of healthy controls (n = 5), non-inflamed (n = 13) and inflamed UC patients (n = 8) (GEO dataset GSE9452). (E) Volcano plot showing enrichment of murine cytokine gene transcripts in inflamed colonic tissue from cDSS treated mice compared to control tissue (left, n = 5 per group) and qPCR of basal cytokine expression in cDSS colonic tissue (right, n = 5). Transcriptomics data was derived from the GEO dataset GSE42768. Mean ± s.e.m. are indicated. Data are representative of three independent experiments. (F) *Il1b*, *Cxcl1*, and *Cxcl2* expression levels in colonic tissue following 7-day aDSS and 0.5mg anti-*Escherichia coli/Enterobacteriaceae* IgG injection. Data are normalized to PBS control. Means ± s.e.m. are indicated. Data are representative of two independent experiments. (G) qPCR of flow-sorted colonic CX3CR1^{+} Ly6C^{lo} MHC-II^{+} macrophages stimulated for 4h with Ova or Ova-IC (n = 2 per condition). Means ± s.e.m. are indicated. Data are representative of three independent experiments. (H) Serum anti-flagellin IgG titers in mice hyperimmunized with flagellin or PBS in IFA (n = 7 per group). Means ± s.e.m. are indicated. (I) Weight change in *Rag2*$^{-/-}$ mice receiving 0.5 mg anti-flagellin IgG or control IgG and subjected to 7 days of aDSS (n = 5 per group). P values were calculated using limma with multiple correction using BH (A, E), linear regression analysis (B, C), parametric Student's t test (F), or two-way ANOVA with Tukey's multiple comparisons test (G). * $P < 0.05$; *** $P < 0.001$; **** $P < 0.0001$.

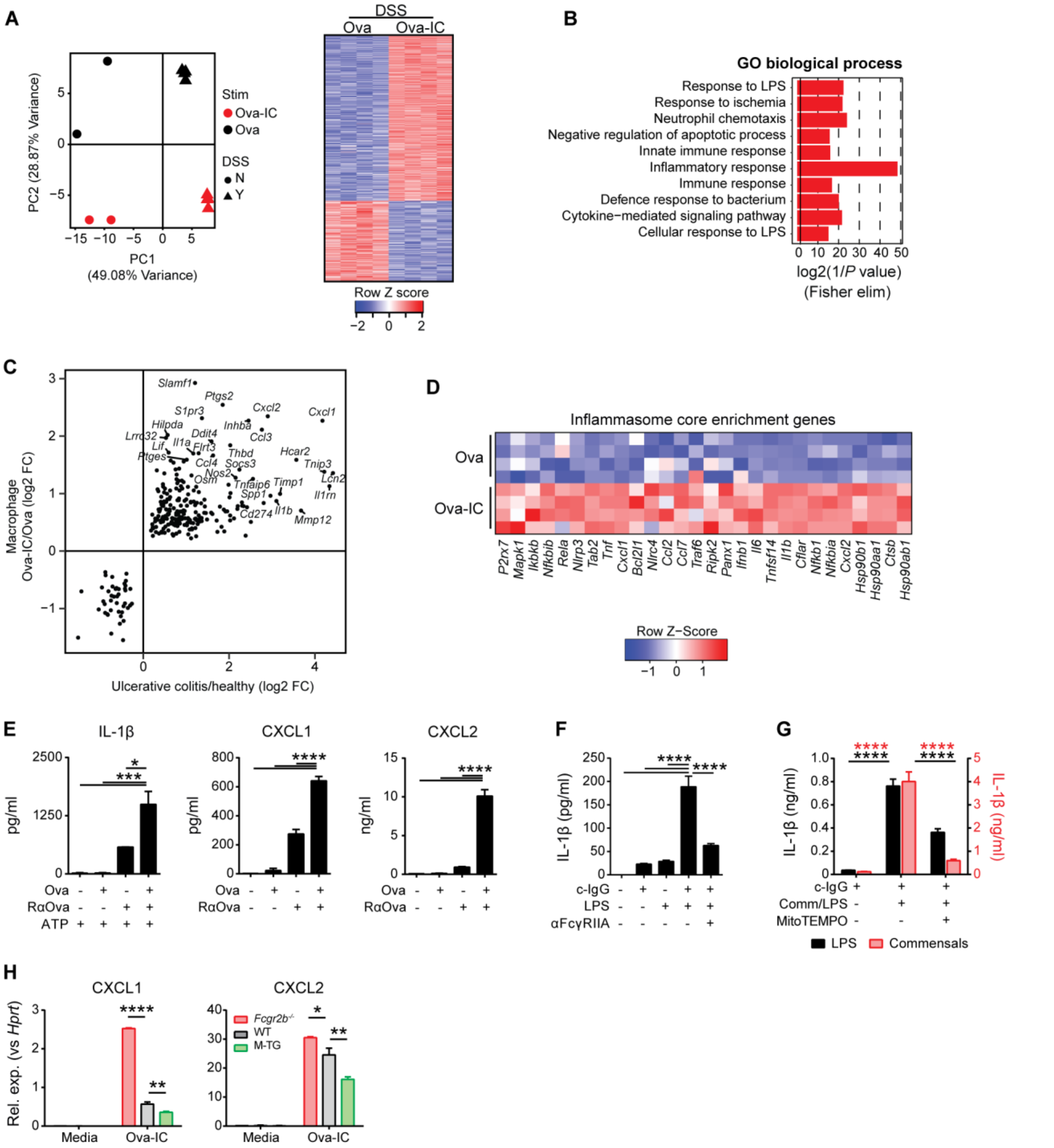

Figure S4. FcγR signaling analysis on intestinal macrophages, related to Figure 4. (A) Principal component plot of intestinal macrophages extracted from aDSS or healthy colons stimulated for Ova or Ova-IC for 4 h (left) and heatmap of differential gene expression in aDSS-macrophages (right) (n = 2-4 per condition). (B) Gene ontology analysis of co-regulated genes between UC and Ova-IC-stimulated macrophages using TopGo. (C) Plot of significant co-regulated genes between UC and Ova-IC-stimulated macrophages, as determined by sdef. (D) Core enrichment genes of inflammasome pathways in intestinal macrophages induced by Ova-IC stimulation (n = 4 per condition). (E) Cytokine induction by murine BMDMs stimulated with anti-Ova immune serum alone compared to Ova or Ova-IC for 4h (n = 3 per condition). Mean ± s.e.m. are indicated. Data are representative of three independent experiments. (F) IL-1β production by human MDMs stimulated with LPS and c-IgG for 18h ± anti-FcγRIIA IgG (n = 3 per condition). Mean ± s.e.m. are indicated. Data are representative of three independent experiments. (G) The effect of

mitochondrial ROS inhibitor mitoTEMPO on commensal- and LPS-induced IL-1β production on MDMs stimulated as in F. Mean ± s.e.m. are indicated. Data are representative of two independent experiments. (H) *Cxcl1* and *Cxcl2* induction in murine BMDMs stimulated for 4 h with Ova or Ova-IC (n = 3 per group). Data are representative of two independent experiments. P values were calculated using the standard DESeq 2 method with multiple correction using BH (A-D), one-way ANOVA with Tukey's multiple comparisons test (E-G), or a two-way ANOVA with Tukey's multiple comparisons test (H).

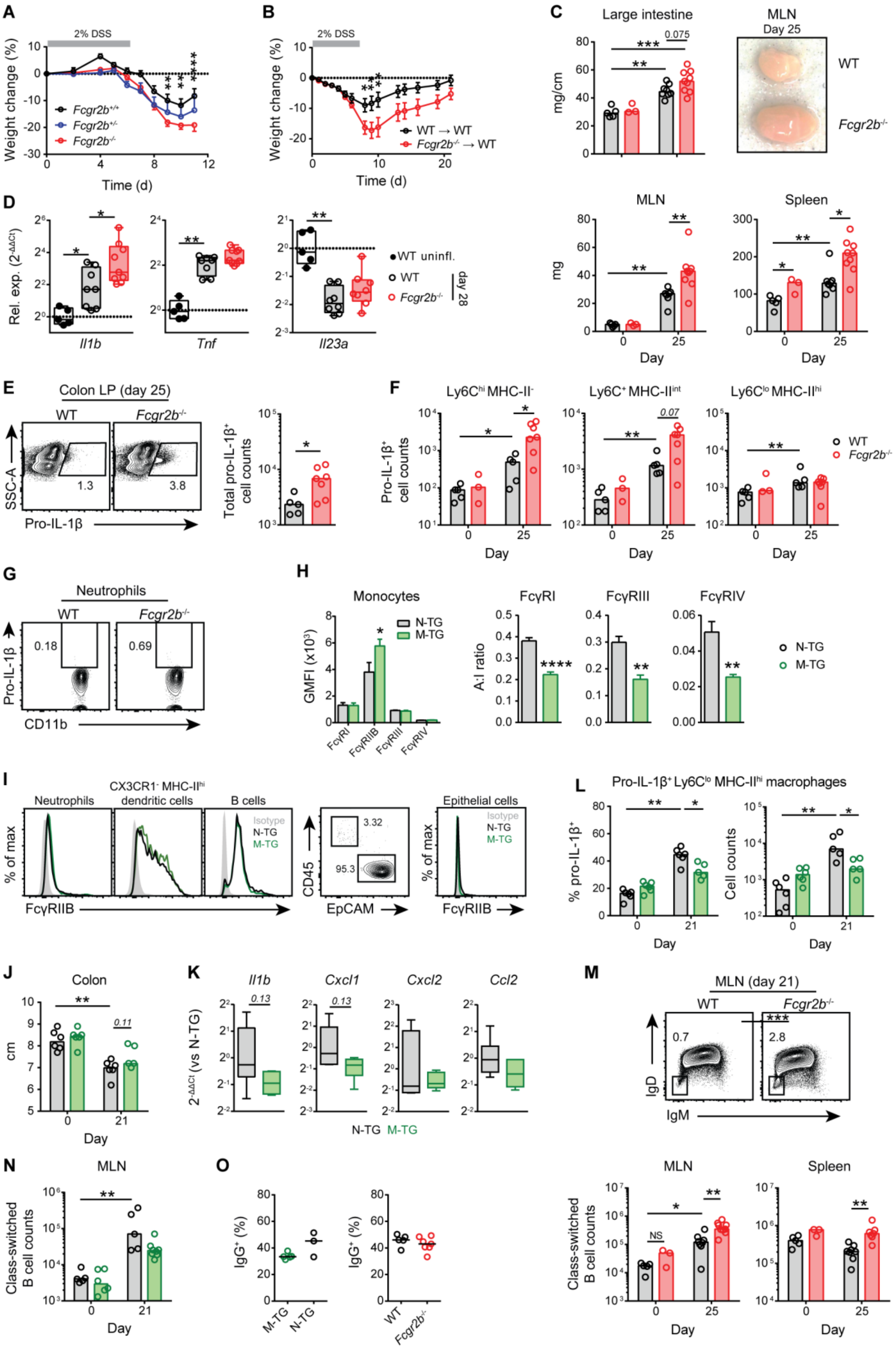

Figure S5. MNP FcγR A:I ratio modulates intestinal inflammation, related to Figure 5. (A, B) Weight loss in *Fcgr2b*$^{+/+}$, *Fcgr2b*$^{+/-}$, and *Fcgr2b*$^{-/-}$ littermate control mice (*n* = 4-6 per group) (A) and WT C57BL/6 mice reconstituted with WT or *Fcgr2b*$^{-/-}$ bone marrow (*n* = 8 per group) (B) following aDSS. Mean ± s.e.m. are indicated. (C) Colon and lymphoid organ weights in WT and *Fcgr2b*-deficient mice at day 25 post-aDSS (*n* = 5-7 per group) or healthy controls (day 0, *n* = 3-5 per group). Medians are indicated. Data are representative of three independent experiments. (D) qPCR of cytokines in whole colonic tissue of WT and *Fcgr2b*$^{-/-}$ mice at day 28 post-aDSS and healthy control colons (*n* = 5-9 per group). Data are normalized to WT uninflamed mRNA levels using the $2^{-\Delta\Delta Ct}$ method and *Gapdh*. Min. to max. box-and-whisker plots are indicated. Data are representative of two independent experiments. (E) Analysis of frequency (left) and absolute cell counts (right) of total pro-IL-1β-expressing cells in WT and *Fcgr2b*$^{-/-}$ mice treated with DSS as in C. For absolute cell counts, medians are indicated. Data are representative of three independent experiments. (F) Quantification of absolute cell counts of colonic pro-IL-1β-expressing waterfall MNP subsets in WT and *Fcgr2b*-deficient mice treated as in C. Medians are indicated. (G) Pro-IL-1β expression by colonic neutrophils in colitic mice treated as in C. (H) FcγR expression analysis and A:I ratio calculation for of colonic monocytes in M-TG and N-TG colons (*n* = 3 per group). Mean ± s.e.m. are indicated. Data are representative of two independent experiments. (I) FcγRIIB expression by colonic leukocytes and intestinal epithelial cells in M-TG and N-TG mice. Data are representative of three independent experiments. (J) Colon length in M-TG and N-TG at day 21 post-aDSS (*n* = 5-6 per group) or in healthy controls (day 0, *n* = 6 per group)). Medians are indicated. Data are representative of three independent experiments. (K) qPCR of cytokines and chemokines in whole colonic tissue from N-TG and M-TG mice treated as in J (*n* = 5 per group). Data are normalized to N-TG mRNA levels using the $2^{-\Delta\Delta Ct}$ method and *Gapdh*. Min. to max. box-and-whisker plots are shown. (L) Frequency of pro-IL-1β expression by colonic Ly6C^{lo} MHC-II^{hi} macrophages (left) and absolute counts of pro-IL-1β-expressing macrophages (right) in M-TG and N-TG mice treated as in J. Medians are indicated. (M) Analysis of the frequency (top) and absolute cell counts (bottom) of class-switched IgM^{-} IgD^{-} B cells in the MLN and spleen of WT and *Fcgr2b*$^{-/-}$ mice at day 21 post-aDSS. Medians are indicated. Data are representative of three independent experiments. (N) Absolute cell counts of class-switched B cells in the MLN of M-TG and N-TG mice treated as in J. Medians are indicated. Data are representative of two independent experiments. (O) IgG binding of day 21 aDSS serum from N-TG/M-TG (left, *n* = 3-5 per group) and WT/*Fcgr2b*$^{-/-}$ mice (right, *n* = 5-7 per group) to *Rag2*-deficient fecal commensals. Medians are indicated. Data are representative of two independent experiments. *P* values were calculated using a two-way ANOVA with Bonferroni's multiple comparisons test (A, B), the nonparametric Mann-Whitney *U* test (C-F, K-O), or Student's two-tailed *t* test (H). * $P < 0.05$; ** $P < 0.01$; *** $P < 0.001$; **** $P < 0.0001$.

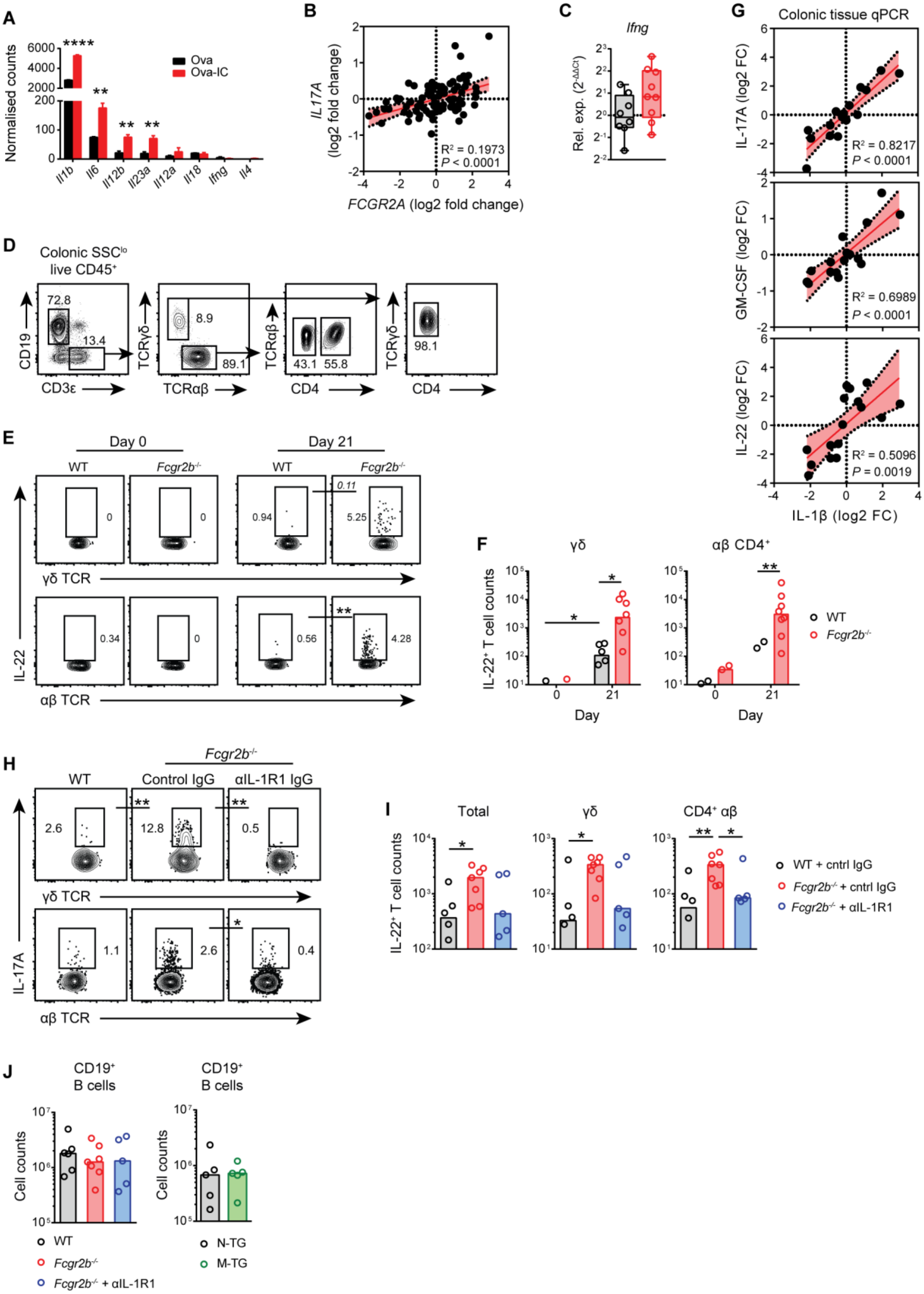

A
Normalised counts
Ova
Ova-IC
Il1b
Il6
Il12b
Il23a
Il12a
Il18
Ifng
Il4
B
IL17A (log2 fold change)
FCGR2A (log2 fold change)
R² = 0.1973
P < 0.0001
C
Ifng
Rel. exp. (2^-ΔΔCt)
G
Colonic tissue qPCR
IL-17A (log2 FC)
R² = 0.8217
P < 0.0001
GM-CSF (log2 FC)
R² = 0.6989
P < 0.0001
IL-22 (log2 FC)
R² = 0.5096
P = 0.0019
IL-1β (log2 FC)
D
Colonic SSClo live CD45+
CD19
CD3ε
72.8
13.4
TCRγδ
TCRαβ
8.9
89.1
TCRαβ
CD4
43.1
55.8
TCRγδ
CD4
98.1
E
Day 0
Day 21
WT
Fcgr2b-/-
0.11
0.94
5.25
0.34
0.56
4.28
γδ TCR
IL-22
αβ TCR
F
γδ
αβ CD4+
IL-22+ T cell counts
Day
WT
Fcgr2b-/-
H
WT
Fcgr2b-/-
Control IgG
αIL-1R1 IgG
2.6
12.8
0.5
1.1
2.6
0.4
γδ TCR
IL-17A
αβ TCR
I
Total
γδ
CD4+ αβ
IL-22+ T cell counts
WT + cntrl IgG
Fcgr2b-/- + cntrl IgG
Fcgr2b-/- + αIL-1R1
J
CD19+ B cells
Cell counts
WT
Fcgr2b-/-
Fcgr2b-/- + αIL-1R1
N-TG
M-TG

Figure S6. MNP FcγR A:I ratio modulates intestinal type 17 immunity, related to Figure 6. (A) Expression of T cell-inducing cytokines in flow-sorted colonic Ly6C^{lo} MHC-II^{hi} macrophages from colitic mice stimulated with Ova or Ova-IC for 4h (n = 4 per condition). Mean ± s.e.m. are indicated. Data are representative of three independent experiments. (B) Correlation of *FCGR2A* with *IL17A* gene transcripts in colonic biopsies pooled from healthy controls and UC patients (n = 85). Data were derived from GEO dataset GSE59071. (C) qPCR of *Ifng* in whole colonic tissue from WT and *Fcgr2b*$^{-/-}$ mice at day 28 post-aDSS (n = 8-9 per group). Medians are indicated. (D) Flow cytometry gating strategy for quantification of colonic lymphocytes (left). (E, F) Quantification of the frequency (E) and absolute cell counts (F) of colonic IL-22 T cell subsets in WT and *Fcgr2b*$^{-/-}$ mice at day 21 post-aDSS (n = 6-7 per group) or healthy controls (day 0, n = 3-5 per group). For absolute cell counts, medians are indicated. Data are representative of three independent experiments. (G) Correlation of type 17-associated cytokines and IL-1β mRNA levels in whole colonic tissue pooled from WT and *Fcgr2b*$^{-/-}$ colitic mice by qPCR (n = 16). Data are representative of two independent experiments. (H) Frequency of colonic IL-17A-producing T cell subsets in WT and *Fcgr2b*$^{-/-}$ mice at day 15 post-aDSS and treated with control IgG or anti-IL-1R1 IgG blocking antibody (n = 5-7 per group). Data are representative of two independent experiments. (I) Absolute counts of IL-22-producing T cell subsets in inflamed colons of mice treated as in H. (J) Colonic CD19^{+} B cell counts in WT and *Fcgr2b*$^{-/-}$ mice at day 15 post-aDSS plus anti-IL-1R1 or control IgG administration as in H (left, n = 5-7 per group) and N-TG and M-TG mice at day 21 post-aDSS (right, n = 5 per group). Medians are indicated. Data are representative of two or three independent experiments. P values were calculated using the standard DESeq 2 method with multiple correction using BH (A), linear regression analysis (B, G), or nonparametric Mann-Whitney U test (E-J). * $P < 0.05$; ** $P < 0.01$.

Table S1. Patient characteristics of Cambridge cohort used for microbial flow cytometry, related to Figure 1.

Pair #	Sex	Age	Status	Walmsley CAI score	CRP (mg/l)	Medication at sampling
1	F	28	UC	12	72	None
	M	28	HHC	-	-	-
2	M	42	UC	1	<4	Prednisolone<br>Salofalk
	F	37	HHC	-	-	-
3	M	20	UC	6	N/A	Inpatient<br>Co-trimoxazole<br>Cyclosporine<br>Hydrocortisone<br>Ciprofloxacin<br>Metronidazole
	F	55	HHC	-	-	-
4	M	33	UC	13	<4	Inpatient<br>Methylprednisolone<br>Hydrocortisone
	F	26	HHC	-	-	-
5	M	65	UC	5	N/A	Adcal<br>Asacol<br>Prednisolone
	F	51	HHC	-	-	-
6	F	28	UC	5	<4	Azathioprine<br>Methylprednisolone
	M	29	HHC	-	-	

UC = ulcerative colitis.
HHC = Household healthy control.

Table S2. Baseline characteristics of infliximab-refractory UC dataset (GSE16879) (Arijs et al., 2009a; Arijs et al., 2009b), related to Figure 2.

**Characteristic**	**Responders (*n* = 8)**	**Non-responders (*n* = 16)**
Male/female (%)	4/4 (50/50)	10/6 (62.5/37.5)
Median (IQR) age at first IFX (years)	28.4 (24.3-41.8)	45.8 (36.5-62.3)
Median (IQR) weight at first IFX (kg)	72 (57.8-78.5)	73.3 (68.5-80.3)
Median (IQR) duration of disease prior to first IFX (years)	10.3 (4.1-17.3)	7.3 (2.6-13.3)
Median (IQR) C-reactive protein at first IFX (mg/dl)	1.65 (1-9.6)	6.5 (2.9-19.1)
Median (IQR) Mayo score before first IFX	10 (8.8-10)	9.5 (8.8-10.3)
Concomitant medication at first IFX (%)		
5-Aminosalicylates	5 (62.5)	13 (81.3)
Corticosteroids	2 (25)	5 (31.3)
Azathioprine/6-mercaptopurine	7 (87.5)	8 (50)
Corticosteroids + immunosuppressants	2 (25)	1 (6.3)
Active smoking at first IFX (%)	1 (12.5)	1 (6.3)
